# Supplementary material for: Constructing multiple active sites in iron oxide catalysts for improving carbonylation reactions
Source: Nat Commun. 2023 Aug 17;14:4973. doi: 10.1038/s41467-023-40640-z (PMC10435489; doi:10.1038/s41467-023-40640-z)
Supplement: Supplementary file 1 — Supplementary Information [file 41467_2023_40640_MOESM1_ESM.pdf]

Supporting Information for

Constructing multiple active sites in iron oxide catalysts for improving carbonylation reactions

Shujuan Liu[1,†], Teng Li[1,†], Feng Shi[1], Haiying Ma[1,2], Bin Wang[1], Xingchao Dai[1] and Xinjiang Cui[1✉]

[1] State Key Laboratory for Oxo Synthesis and Selective Oxidation, Lanzhou Institute of Chemical Physics, Chinese Academy of Sciences, No. 18, Tianshui Middle Road, Lanzhou 730000, China.

[2] University of Chinese Academy of Sciences, No. 19A, Yuquan Road, Beijing 100049, China.

[†]These authors contributed equally: Shujuan Liu, Teng Li.

✉e-mail: xinjiangcui@licp.cas.cn

1. Synthesis of 1.0 Fe_2O_3-O_{vac} (Cu), 1.0 Fe_2O_3-O_{vac} (Ni), and 1.0 Fe_2O_3-O_{vac} (Pd).

500 mg anhydrous ferric chloride, 0.33 mg $CuCl_2$•$2H_2O$ and 2 g CTAB (cetyltrimethyl ammonium bromide) were dissolved in 60 mL deionized. After the mixture became clear solution, the above mixture was transferred into a 100 mL Teflon-lined stainless-steel autoclave and heated to 120 °C for 24 h and then cooled to room temperature naturally. The resulting product was collected by filtration, washed several times with deionized water and absolute ethanol, and then dispersed in absolute ethanol and dried at 80 °C in air overnight. The sample was labeled as Fe_2O_3(Cu). Fe_2O_3(Ni) and Fe_2O_3(Pd) were obtained with 0.50 mg $NiCl_2$•$6H_2O$ and 0.21 mg $PdCl_2$, respectively. The synthesis of 1.0 Fe_2O_3-O_{vac} (Cu), 1.0 Fe_2O_3-O_{vac} (Ni), and 1.0 Fe_2O_3-O_{vac} (Pd) were same with the 1.0 Fe_2O_3-O_{vac}.

2. Catalyst characterization

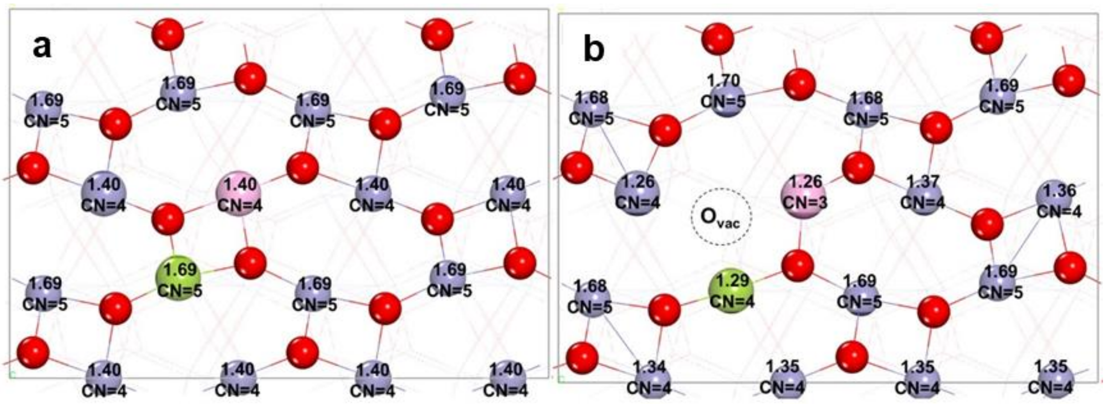

Supplementary Fig. 1 Bader charge and coordination number (CN) of iron atoms in Fe_2O_3(104) (**a**) and Fe_2O_3(104)-O_{vac} (**b**) surfaces.

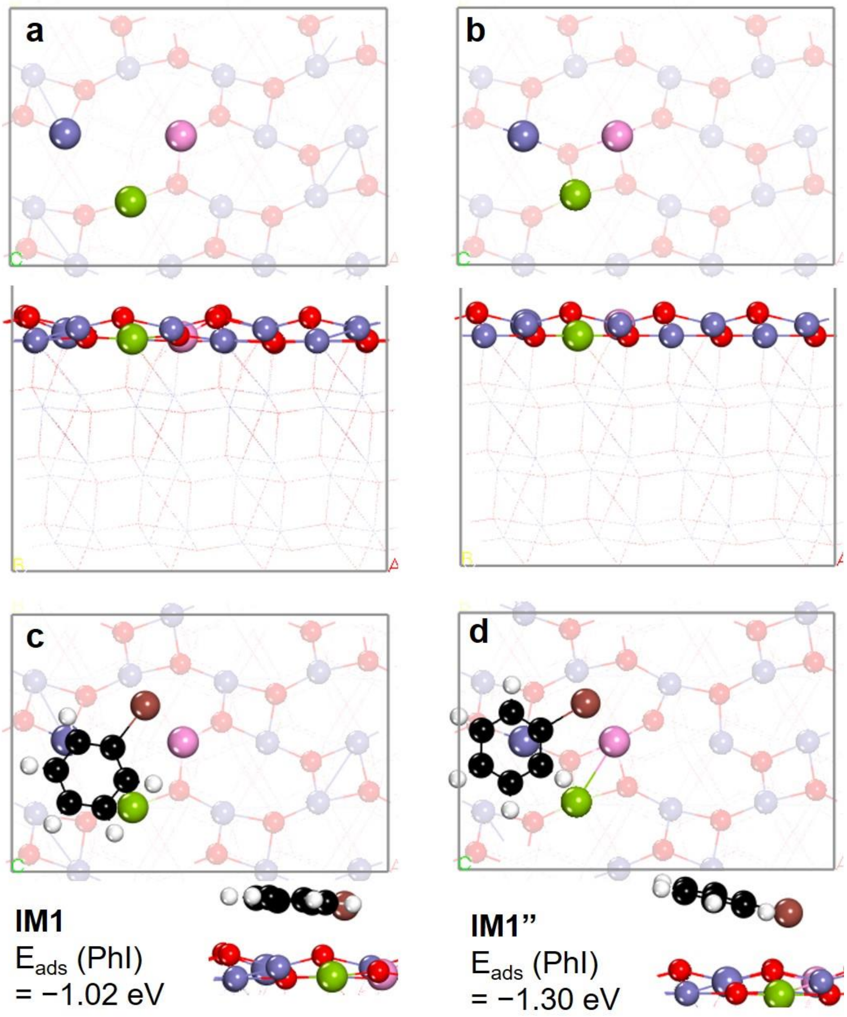

Supplementary Fig. 2 Structures of Fe_2O_3(104)-O_{vac} (**a**) and Fe_2O_3(104) (**b**) models, adsorption of PhI on Fe_2O_3(104)-O_{vac} (**c**) and Fe_2O_3(104) (**d**) surfaces.

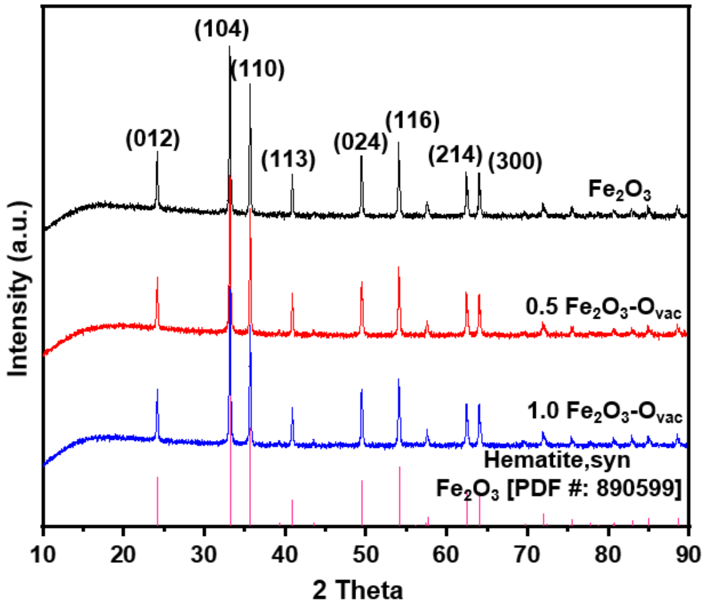

Supplementary Fig. 3 XRD spectra of Fe_2O_3, 0.5 Fe_2O_3-O_{vac} and 1.0 Fe_2O_3-O_{vac}.

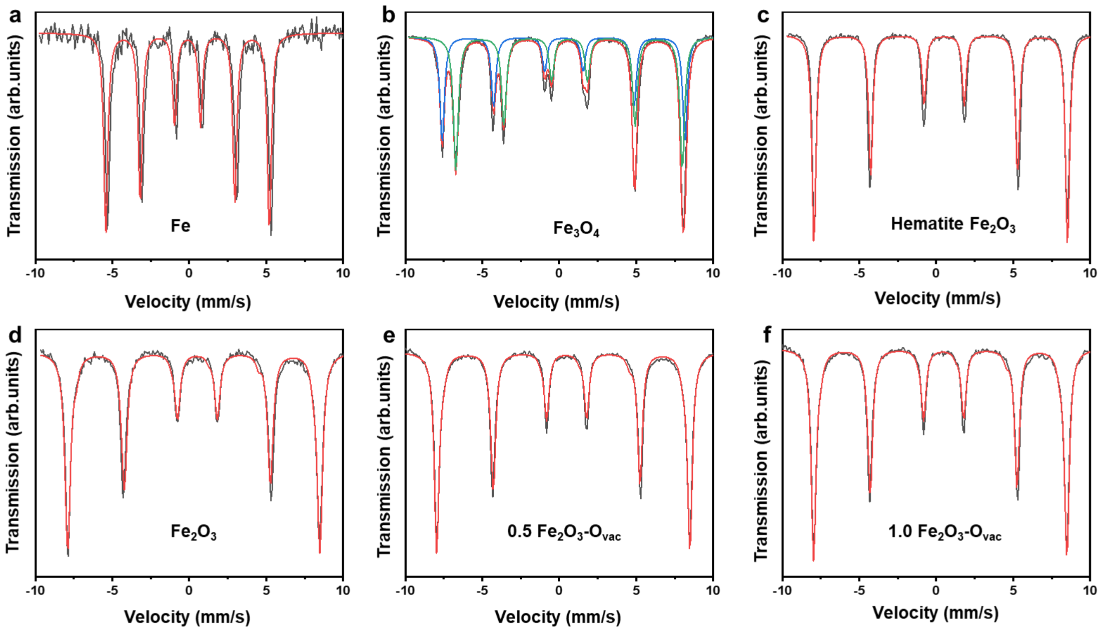

Supplementary Fig. 4 The [57]Fe mössbauer spectroscopy of Fe (**a**), Fe_3O_4 (**b**), Hematite Fe_2O_3 (**c**), Fe_2O_3 (**d**), 0.5 Fe_2O_3-O_{vac} (**e**) and 1.0 Fe_2O_3-O_{vac} (**f**).

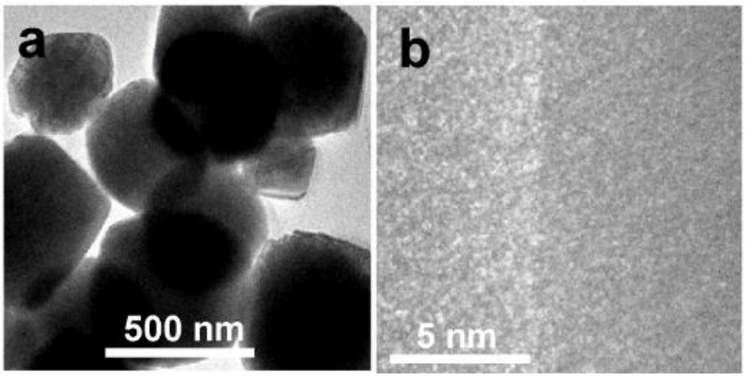

Supplementary Fig. 5 TEM (**a**) and HR-TEM images (**b**) of 2.0 Fe_2O_3-O_{vac}.

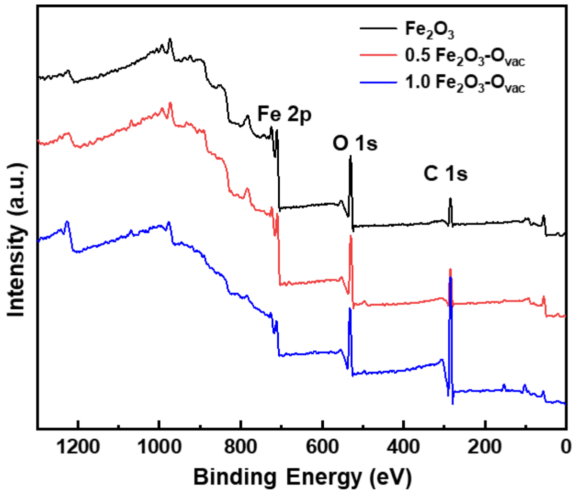

Supplementary Fig. 6 XPS survey spectra for Fe_2O_3, 0.5 Fe_2O_3-O_{vac}, and 1.0 Fe_2O_3-O_{vac}.

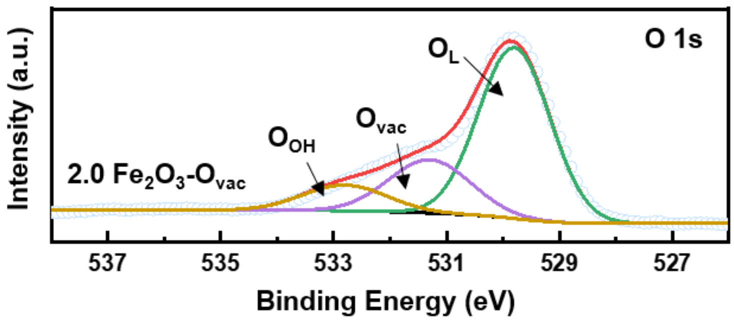

Supplementary Fig. 7 XPS of O 1s for 2.0 Fe_2O_3-O_{vac}.

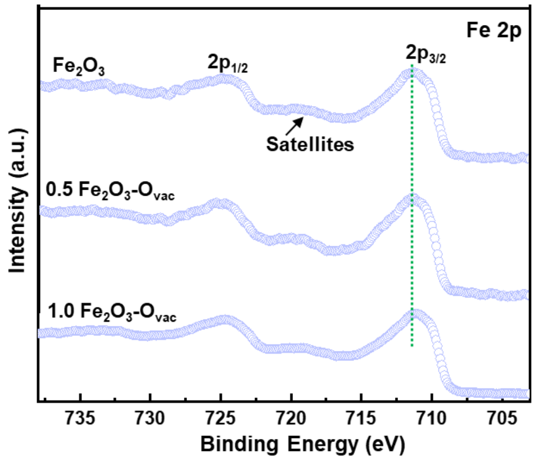

Supplementary Fig. 8 XPS spectra of Fe 2p for Fe_2O_3, 0.5 Fe_2O_3-O_{vac} and 1.0 Fe_2O_3-O_{vac}.

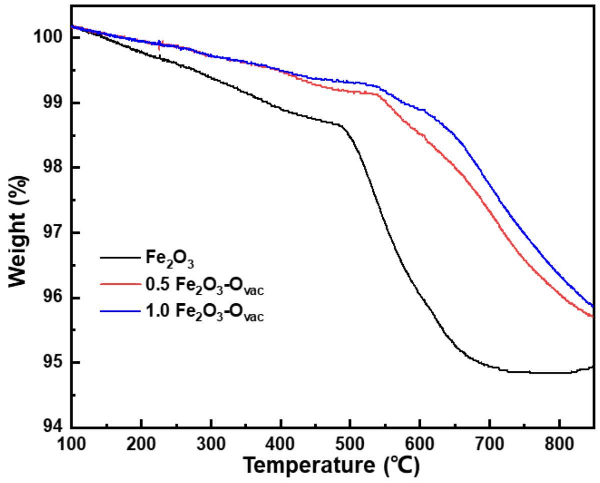

Supplementary Fig. 9 H_2-TGA for Fe_2O_3, 0.5 Fe_2O_3-O_{vac} and 1.0 Fe_2O_3-O_{vac}.

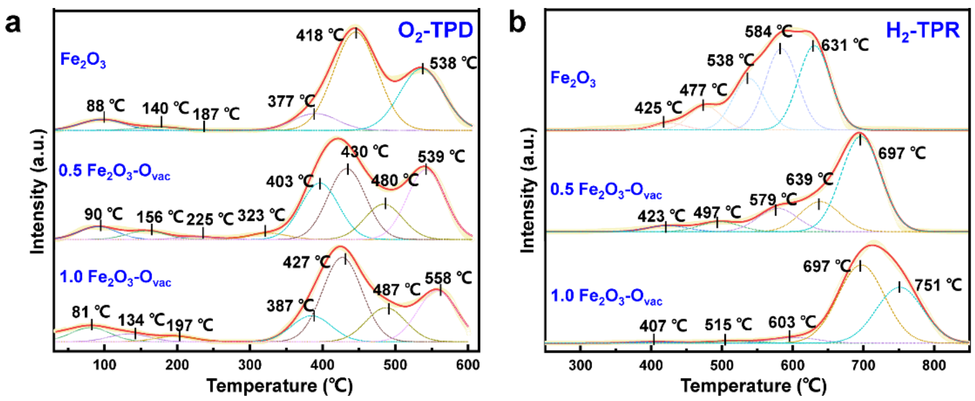

Supplementary Fig. 10 O_2-TPD (**a**) and H_2-TPR (**b**) of Fe 2p for Fe_2O_3, 0.5 Fe_2O_3-O_{vac} and 1.0 Fe_2O_3-O_{vac}.

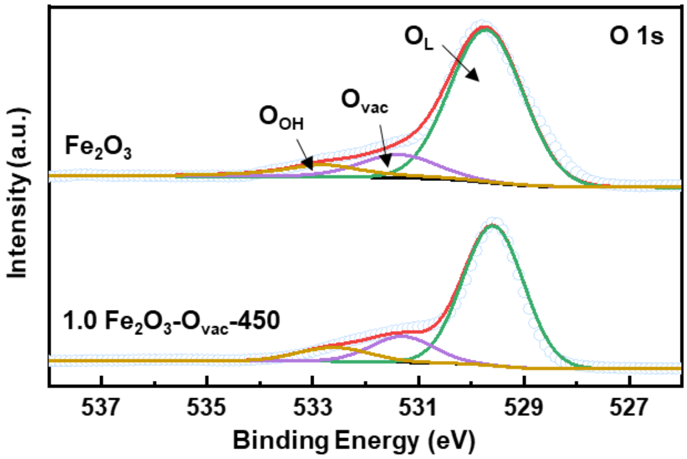

Supplementary Fig. 11 XPS of O 1s for Fe_2O_3 and 1.0 Fe_2O_3-O_{vac}-450.

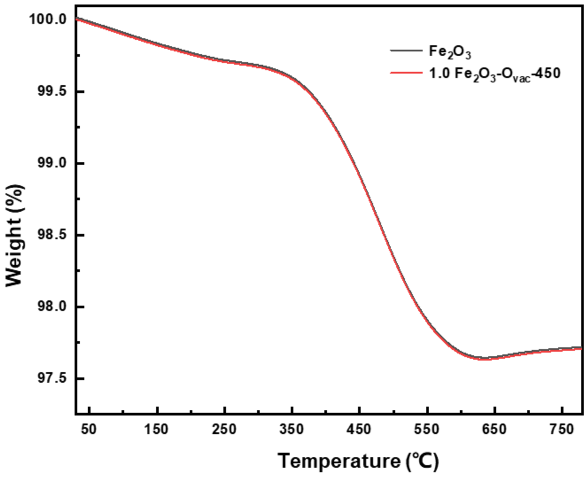

Supplementary Fig. 12 TGA of Fe_2O_3 and 1.0 Fe_2O_3-O_{vac}-450.

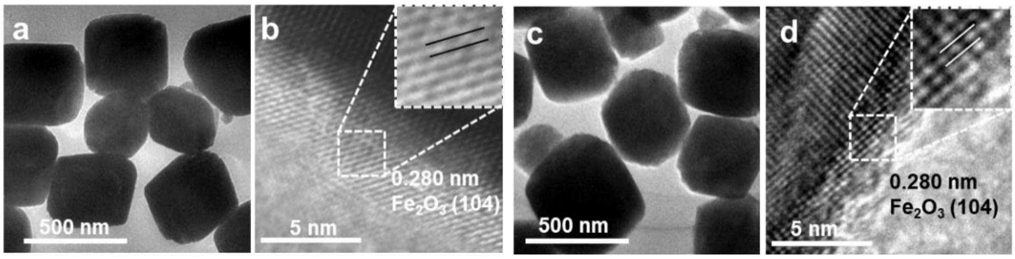

Supplementary Fig. 13 TEM images of 1.0 Fe_2O_3-O_{vac} (**a**) and 1.0 Fe_2O_3-O_{vac}-used (**c**); HR-TEM images of 1.0 Fe_2O_3-O_{vac} (**b**) and 1.0 Fe_2O_3-O_{vac}-used (**d**).

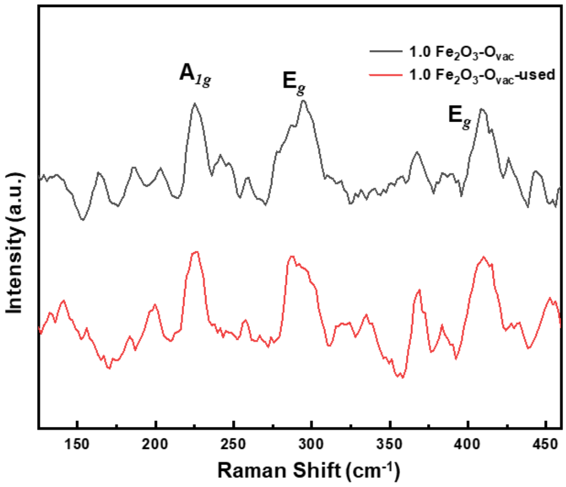

Supplementary Fig. 14 Raman image of 1.0 Fe_2O_3-O_{vac} and 1.0 Fe_2O_3-O_{vac}-used.

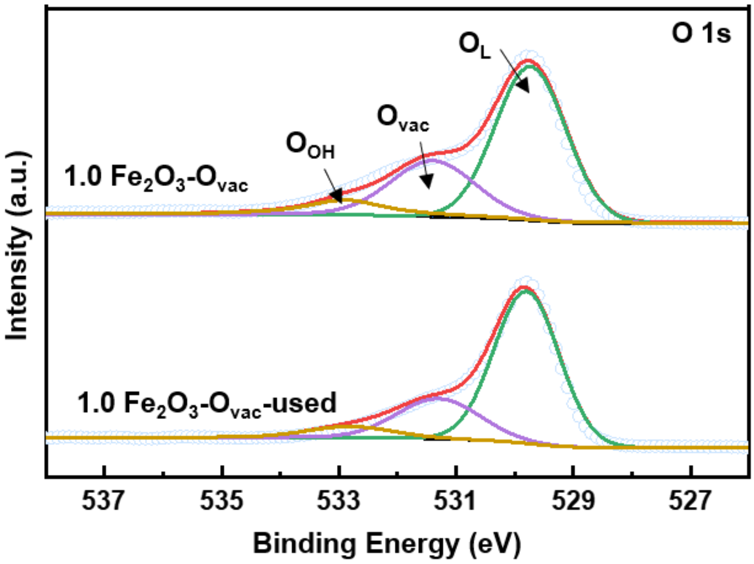

Supplementary Fig. 15 XPS of O 1s for 1.0 Fe_2O_3-O_{vac} and 1.0 Fe_2O_3-O_{vac}-used.

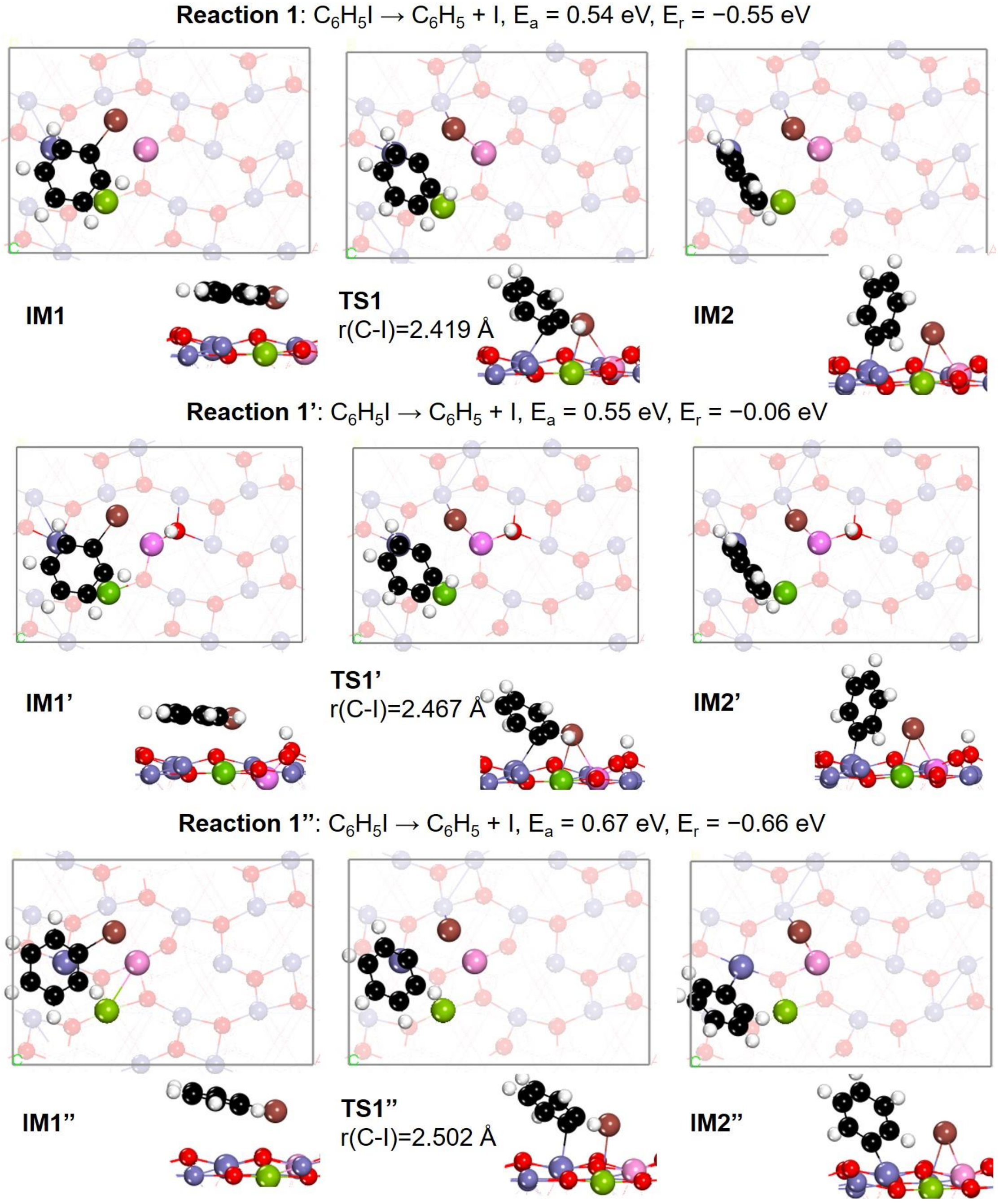

Supplementary Fig. 16 Structures of iodobenzene was adsorbed at Fe_2O_3(104)-O_{vac} surface with O_{vac} (**IM1**), Fe_2O_3(104)-O_{vac} with hydroxyl (**IM1’**), and normal Fe_2O_3(104) (**IM1”**). Structural evolution of PhI decomposition on Fe_2O_3(104)-O_{vac} without hydroxyl (**Reaction 1**), Fe_2O_3(104)-O_{vac} with hydroxyl (**Reaction 1’**), normal Fe_2O_3(104) (**Reaction 1”**).

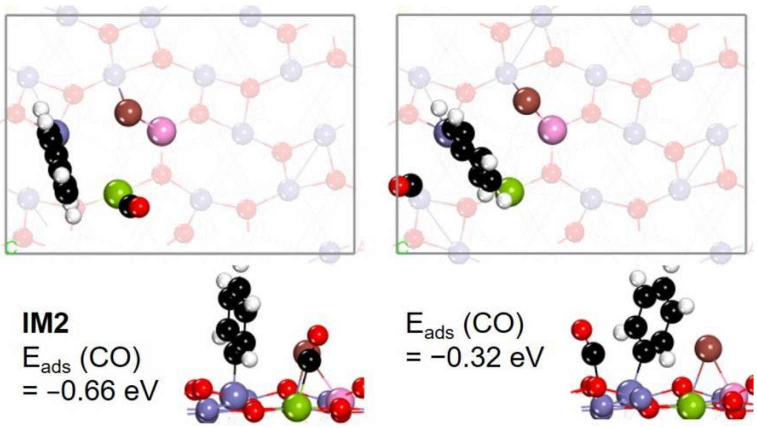

Supplementary Fig. 17 Adsorption structures of CO after PhI decomposition on Fe_2O_3(104)-$O_{vac.}$

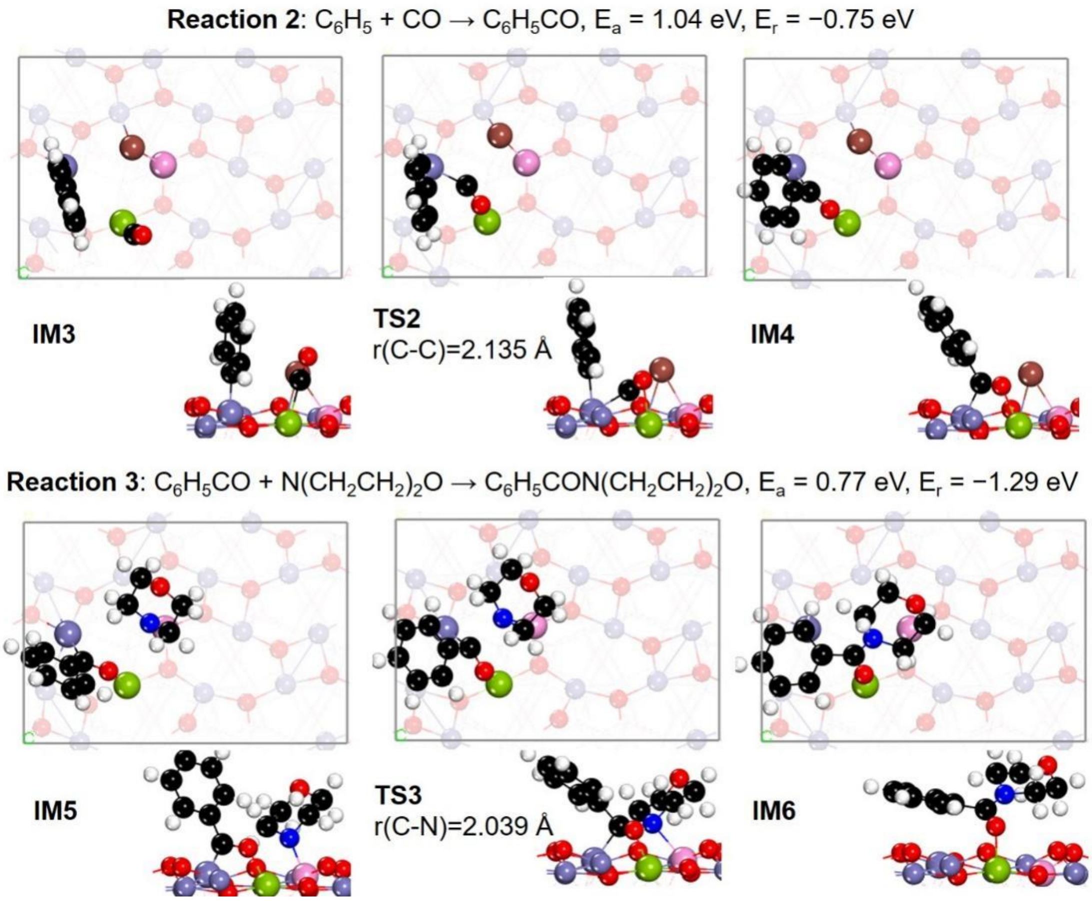

Supplementary Fig. 18 Structure evolution of phenyl carbonylation and C-N coupling on Fe_2O_3(104)-$O_{vac.}$

Supplementary Table 1. ICP-OES data of Fe, Cu, Ni and Pd for $FeCl_3$, Fe_2O_3, 0.5 Fe_2O_3-O_{vac} and 1.0 Fe_2O_3-O_{vac}.

**Cat.**	Fe (mg/L)	Cu (mg/L)	Ni (mg/L)	Pd (mg/L)
$FeCl_3$	64.59	-	-	-
Fe_2O_3	135.07	-	-	-
0.5 Fe_2O_3-O_{vac}	135.62	-	-	-
1.0 Fe_2O_3-O_{vac}	136.89	-	-	-

Supplementary Table 2. The effect of Cu, Ni and Pd for the carbonylation of iodobenzene and morpholine [a]

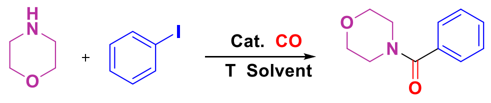

1a 2a 3a

**Entry**	**Catalyst**	**T/°C**	**CO/MPa**	**Solvent**	**Yield.[b]/%**
1	1.0 Fe_2O_3-O_{vac}	160	1	1,4-Dioxane	58
2	5 wt%Fe + 1.0 Fe_2O_3-O_{vac}	160	1	1,4-Dioxane	11
3[c]	Fe NPs	160	1	1,4-Dioxane	15
4[d]	Fe NPs	160	1	1,4-Dioxane	12
5[e]	1.0 Fe_2O_3-O_{vac}(Cu)	160	1	1,4-Dioxane	49
6[f]	1.0 Fe_2O_3-O_{vac}(Ni)	160	1	1,4-Dioxane	26
7[g]	1.0 Fe_2O_3-O_{vac}(Pd)	160	1	1,4-Dioxane	55

[a] Reaction conditions: **1a** (1.5 mmol), **2a** (1.0 mmol), Et_3N (2.0 mmol), catalyst (80 mg), solvent (2 mL), 160 °C (reaction temperature), 18 h; [b] Yields were determined by GC-MS; [c] 28 mg Fe NPs; [d] 80 mg Fe NPs; [e] 250 ppm Cu in 1.0 Fe_2O_3-O_{vac}; [f] 250 ppm Ni in 1.0 Fe_2O_3-O_{vac}; [g] 250 ppm Pd in 1.0 Fe_2O_3-O_{vac}.

Supplementary Table 3. Mössbauer parameters *IS*, (ε_Q), B_{hf} and relative area for Fe_2O_3, 0.5 Fe_2O_3-O_{vac} and 1.0 Fe_2O_3-O_{vac}.

**Cat.**	*IS* (mm/s)	ε_Q (mm/s)	B_{hf} (T)
Fe_2O_3	0.41	-0.233	50.9
0.5 Fe_2O_3-O_{vac}	0.37	-0.221	51.1
1.0 Fe_2O_3-O_{vac}	0.37	-0.221	51.1
hematite Fe_2O_3	0.40	-0.220	51.2
Fe_3O_4	0.29	-0.023	49.0
	0.65	-0.029	45.7

Supplementary Table 4. The relative content of surface lattice oxygen (O_L), oxygen vacancies (O_{vac}) and hydroxyl species of surface-adsorbed water molecules (O_{OH}).

Cat.	O 1s		
	O_L (%)	O_{vac} (%)	O_{OH} (%)
Fe_2O_3	77	13	10
0.5 Fe_2O_3-O_{vac}	75	18	7
1.0 Fe_2O_3-O_{vac}	64	26	10
2.0 Fe_2O_3-O_{vac}	66	23	11
1.0 Fe_2O_3-O_{vac}-used	67	26	7
1.0 Fe_2O_3-O_{vac}-450	76	14	10

Supplementary Table 5. H_2 consumption of H_2-TPR.

Cat.	H_2-TPR
	H_2 consumption(umol/g)
Fe_2O_3-O_{vac}	170
0.5 Fe_2O_3-O_{vac}	160
1.0 Fe_2O_3-O_{vac}	152

Supplementary Table 6. EXAFS data fitting results of Fe_2O_3, 0.5 Fe_2O_3-O_{vac} and 1.0 Fe_2O_3-O_{vac}.

Sample	Path	N	R(Å)	σ^2	E_0(eV)	R
Fe_2O_3	Fe-O	3.8	1.95	0.00570	-1.244	0.00690
	Fe-O	2.2	2.12	0.00570		
	Fe-Fe	4.4	2.96	0.00670		
	Fe-Fe	3.5	3.38	0.00670		
	Fe-Fe	4.4	3.69	0.00670		
0.5 Fe_2O_3-O_{vac}	Fe-O	3.5	1.94	0.00465	-1.913	0.00955
	Fe-O	2.3	2.11	0.00465		
	Fe-Fe	4.4	2.95	0.00658		
	Fe-Fe	3.5	3.38	0.00658		
	Fe-Fe	4.4	3.69	0.00658		
1.0 Fe_2O_3-O_{vac}	Fe-O	3.7	1.96	0.00731	-2.282	0.00682
	Fe-O	1.7	2.12	0.00731		
	Fe-Fe	3.8	2.95	0.00687		
	Fe-Fe	3.2	3.38	0.00687		
	Fe-Fe	3.9	3.69	0.00687		

Supplementary Table 7. Homogeneous and heterogeneous noble metal palladium catalyzed carbonylation of aryl halides using amines with CO.

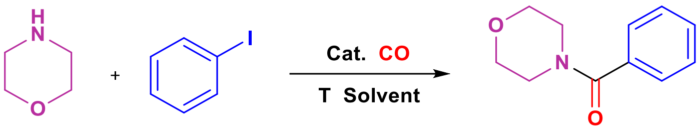

Entry	Catalyst	Base	T/°C	CO/MPa	Solvent	Yield/%	Ref.
1	1.4 mol% PS-Pd-NHC	Na_2CO_3	100	0.7	H_2O	79	1
2	5 mol% $PdCl_2$	--	80	0.1	PEG400/H_2O(7:1)	68	2
3	2 mol % $Pd(TMHD)_2$	Et_3N	100	1.4	Toluene	84	3
4	0.5 mol% $Pd(OAc)_2$	Et_3N	100	0.7	H_2O	82	4
5	1.4 mol % $Pd_2(dba)_3$/SILP	DBU	120	0.5	1,4-Dioxane	70	5
6	1 mol % Pd^0/Cyclic PLLA/clay	t-BuOK	120	0.8	Toluene	83	6
7	0.78 mol% Pd/CFP	Cs_2CO_3	120	4	Toluene	37	7
8	1 mol% nano Pd	K_2CO_3	130	1.2	[TBTdP][DBS]	74	8
9	$Fe_2(CO)_9$/ligands	Cs_2CO_3	90	0.6	Toluene	83	9
10	CuTc/ $Fe_3(CO)_{12}$/ligands	NaOMe	80	4.0	Toluene	81	10

Note: [TBTdP][DBS] = tri-n-butyltetradecylphosphonium dodecylbenzenesulfonate.

CuTc = copper(I)-thiophene-2-carboxylate.

Supplementary Table 8. The control experiments of mechanism for the carbonylation of iodobenzene and morpholine [a]

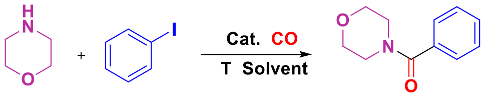

1a **2a** **3a**

**Entry**	**Catalyst**	**T/°C**	**CO/MPa**	**Solvent**	**Yield.[b]/%**
1	1.0 Fe_2O_3-O_{vac}	160	1	1,4-Dioxane	98
2[c]	1.0 Fe_2O_3-O_{vac}	160	1	1,4-Dioxane	99
3[d]	1.0 Fe_2O_3-O_{vac}	160	1	1,4-Dioxane	99

[a] Reaction conditions: **1a** (1.5 mmol), **2a** (1.0 mmol), Et_3N (2.0 mmol), CO (1.0 MPa), catalyst (80 mg), solvent (2 mL), 160 °C (reaction temperature), 24 h; [b] Yields were determined by GC-MS; [c] 1.5 mmol TEMPO; [d] **1a** (1 mmol).

3. Characterization data for products

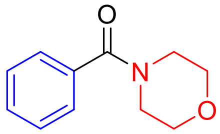

3a Morpholino(phenyl)methanone[11]: The title compound was prepared according to the general procedure and purified by column chromatography using petroleum ether/diethyl ether (5:1) to give the desired product; **^{1}H NMR** (400 MHz, $CDCl_3$) δ 7.43-7.29 (m, 5H), 3.77-3.45 (m, 8H); **^{13}C NMR** (100 MHz, $CDCl_3$) δ 170.3, 135.2, 129.8, 128.4, 127.0, 66.8, 48.1, 42.4.

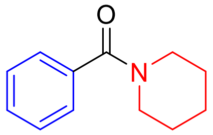

3b phenyl(piperidin-1-yl)methanone[12]: The title compound was prepared according to the general procedure and purified by column chromatography using petroleum ether/diethyl ether (5:1) to give the desired product; **^{1}H NMR** (400 MHz, $CDCl_3$) δ 7.40-7.36 (m, 5H), 3.70 (s, 2H), 3.33 (s, 2H), 1.68-1.66 (m, 4H), 1.51 (s, 2H); **^{13}C NMR** (100 MHz, $CDCl_3$) δ 170.3, 136.5, 129.3, 128.3, 126.7, 48.7, 43.1, 26.5, 25.6, 24.6.

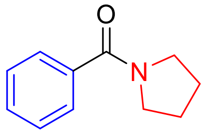

3c phenyl(pyrrolidin-1-yl)methanone[12]: The title compound was prepared according to the general procedure and purified by column chromatography using petroleum ether/diethyl ether (5:1) to give the desired product; **^{1}H NMR** (400 MHz, $CDCl_3$) δ 7.52-7.50 (m, 2H), 7.40-7.38 (m, 2H), 3.64 (t, J = 7.0 Hz, 1H), 3.42 (t, J = 6.5 Hz, 1H), 1.95 (p, J = 6.8 Hz, 1H), 1.86 (p, J = 6.7 Hz, 1H); **^{13}C NMR** (100 MHz, $CDCl_3$) δ 169.5, 137.0, 129.5, 128.0, 126.8, 49.4, 45.9, 26.2, 24.2.

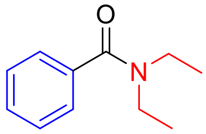

3d *N,N*-diethylbenzamide[13]: The title compound was prepared according to the general procedure and purified by column chromatography using petroleum ether/diethyl ether (5:1) to give the desired product; **^{1}H NMR** (400 MHz, $CDCl_3$) δ 7.47-7.27 (m, 5H), 3.55 (q, J = 7.4 Hz, 2H), 3.25 (q, J = 7.4 Hz, 2H), 1.25 (d, J = 7.4 Hz, 3H), 1.11 (d, J = 7.4 Hz, 3H); **^{13}C NMR** (100 MHz, $CDCl_3$) δ 171.2, 137.2, 129.0, 129.3, 126.2, 43.2, 39.1, 14.1, 12.8.

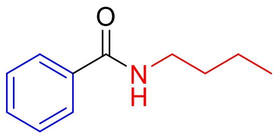

3e *N*-butylbenzamide[14]: The title compound was prepared according to the general procedure and purified by column chromatography using petroleum ether/diethyl ether (5:1) to give the desired product; **^{1}H NMR** (400 MHz, $CDCl_3$) δ 7.77-7.75 (m, 2H), 7.48-7.46 (m, 1H), 7.43-7.38 (m, 2H), 6.36 (brs, 1H), 3.47-3.42 (m, 2H), 1.63-1.56 (m, 2H), 1.43-1.35 (m, 2H), 0.95 (t, *J* = 7.3 Hz, 3H); **^{13}C NMR** (100 MHz, $CDCl_3$) δ 167.5, 134.8, 131.2, 129.4, 125.8, 39.8, 31.7, 20.1, 13.7.

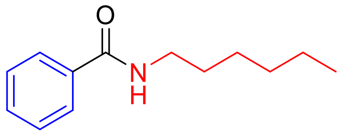

3f *N*-hexylbenzamide[11]: The title compound was prepared according to the general procedure and purified by column chromatography using petroleum ether/diethyl ether (5:1) to give the desired product; **^{1}H NMR** (400 MHz, $CDCl_3$) δ 7.79-7.77 (m, 2H), 7.48-7.44 (m, 1H), 7.40-7.36 (m, 2H), 6.65 (brs, 1H), 3.43-3.38 (m, 2H), 1.62-1.55 (m, 2H), 1.36-1.26 (m, 6H), 0.89 (t, *J* = 7.3 Hz, 3H); **^{13}C NMR** (100 MHz, $CDCl_3$) δ 167.5, 134.7, 131.0, 128.3, 126.8, 40.0, 31.4, 29.5, 26.6, 22.4, 13.9.

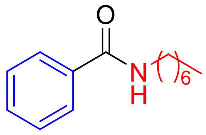

3g *N*-heptylbenzamide[15]: The title compound was prepared according to the general procedure and purified by column chromatography using petroleum ether/diethyl ether (5:1) to give the desired product; **^{1}H NMR** (400 MHz, $CDCl_3$) δ 7.78-7.75 (m, 2H), 7.51-7.46 (m, 1H), 7.44-7.39 (m, 2H), 6.28 (brs, 1H), 3.44 (dd, *J* = 12.9, 7.3 Hz, 2H), 1.65-1.56 (m, 2H), 1.36-1.26 (m, 8H), 0.87 (t, *J* = 6.8 Hz, 3H); **^{13}C NMR** (100 MHz, $CDCl_3$) δ 167.5, 134.8, 131.2, 129.5, 126.8, 40.1, 31.7, 29.6, 29.0, 26.9, 22.6, 14.0.

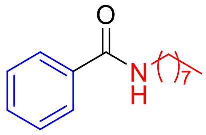

3h *N*-octylbenzamide[15]: The title compound was prepared according to the general procedure and purified by column chromatography using petroleum ether/diethyl ether (5:1) to give the desired product; **^{1}H NMR** (400 MHz, $CDCl_3$) δ 7.78-7.74 (m, 2H), 7.48 (d, *J* = 7.4 Hz, 1H), 7.42 (dd, *J* = 8.2, 6.7 Hz, 2H), 6.20 (brs, 1H), 3.66-3.23 (m, 2H), 1.69-1.49 (m, 2H), 1.43-1.14 (m, 10H), 0.90-0.86

(m, 3H); **^{13}C NMR** (100 MHz, $CDCl_3$) δ 167.5, 134.9, 131.3, 129.5, 126.8, 40.1, 31.8, 29.7, 29.3, 29.2, 26.9, 22.6, 14.0.

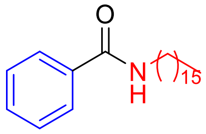

3i *N*-hexadecylbenzamide[16]: The title compound was prepared according to the general procedure and purified by column chromatography using petroleum ether/diethyl ether (5:1) to give the desired product; **^{1}H NMR** (400 MHz, $CDCl_3$) δ 7.80-7.76 (m, 2H), 7.54 – 7.48 (m, 1H), 7.48 – 7.41 (m, 2H), 6.17 (s, 1H), 3.47 (td, *J* = 7.3, 5.8 Hz, 2H), 1.60-1.69 (m, 6.5 Hz, 2H), 1.28-1.40 (m, 26H), 0.90 (t, *J* = 6.8 Hz, 3H); **^{13}C NMR** (100 MHz, $CDCl_3$) δ 167.5, 134.9, 131.3, 129.5, 126.8, 40.1, 31.9, 29.7, 29.7, 29.6, 29.6, 29.5, 29.4, 27.0, 22.7, 14.1.

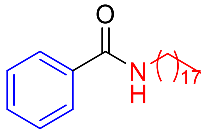

3j *N*-heptadecylbenzamide[17]: The title compound was prepared according to the general procedure and purified by column chromatography using petroleum ether/diethyl ether (5:1) to give the desired product; **^{1}H NMR** (400 MHz, $CDCl_3$) δ 7.81-7.70 (m, 2H), 7.52-7.43 (m, 1H), 7.43-7.38 (m, 2H), 6.14 (brs, 1H), 3.47-3.42 (m, 2H), 1.58-1.65 (m, 2H), 1.30-1.21 (m, 31H), 0.88 (t, *J* = 6.7 Hz, 3H); **^{13}C NMR** (100 MHz, $CDCl_3$) δ 167.5, 134.9, 131.3, 129.5, 126.8, 40.1, 31.9, 29.7, 29.7, 29.6, 29.6, 29.5, 29.3, 27.4, 27.0, 22.7, 14.1.

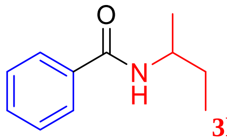

3k *N*-(sec-butyl)benzamide[13]: The title compound was prepared according to the general procedure and purified by column chromatography using petroleum ether/diethyl ether (5:1) to give the desired product; **^{1}H NMR** (400 MHz, $CDCl_3$) δ 7.80-7.72 (m, 2H), 7.54-7.33 (m, 3H), 5.91 (brs, 1H), 4.10-4.17 (m, 1H), 1.55-1.62 m, 2H), 1.23 (d, *J* = 6.6 Hz, 3H), 0.97 (t, *J* = 7.5 Hz, 3H); **^{13}C NMR** (100 MHz, $CDCl_3$) δ 166.9, 135.1, 131.2, 129.5, 126.8, 47.1, 29.8, 20.5, 10.4.

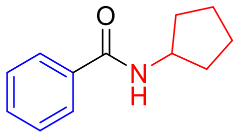

3l *N*-cyclopentylbenzamide[18]: The title compound was prepared according to the general procedure and purified by column chromatography using petroleum ether/diethyl ether (5:1)

to give the desired product; ^{1}**H NMR** (400 MHz, $CDCl_3$) δ 7.81-.66 (m, 2H), 7.55-.34 (m, 3H), 6.08 (brs, 1H), 4.45-4.37 (m, 1H), 2.18-1.99 (m, 2H), 1.80-1.58 (m, 4H), 1.50 (m, 2H); ^{13}**C NMR** (100 MHz, $CDCl_3$) δ 167.1, 134.9, 131.2, 128.5, 126.8, 51.7, 33.2, 23.8.

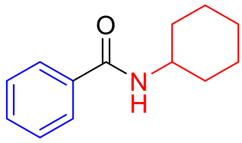

3m ***N*-cyclohexylbenzamide[19]:** The title compound was prepared according to the general procedure and purified by column chromatography using petroleum ether/diethyl ether (5:1) to give the desired product; ^{1}**H NMR** (400 MHz, $CDCl_3$) δ 7.89-.63 (m, 2H), 7.56-7.31 (m, 3H), 6.01 (brs, 1H), 4.00-3.95 (m, 1H), 2.06-2.00 (m, 2H), 1.79-1.63 (m, 3H), 1.48-1.37 (m, 2H), 1.29-1.29 (m, 3H); ^{13}**C NMR** (100 MHz, $CDCl_3$) δ 166.6, 135.1, 131.2, 128.5, 126.8, 48.6, 33.2, 25.6, 24.9.

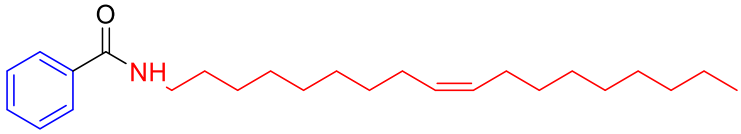

3n ***(Z)*-*N*-(octadec-9-en-1-yl)benzamide[17]:** The title compound was prepared according to the general procedure and purified by column chromatography using petroleum ether/diethyl ether (5:1) to give the desired product; ^{1}**H NMR** (400 MHz, $CDCl_3$) δ 7.82-7.69 (m, 2H), 7.55-7.35 (m, 3H), 6.16 (brs, 1H), 5.46-5.21 (m, 2H), 3.45 (q, *J* = 6.8 Hz, 2H), 2.06-1.95 (m, 3H), 1.65-1.57 (m, 2H), 1.41-1.16 (m, 22H), 0.88 (t, *J* = 6.7 Hz, 3H); ^{13}**C NMR** (100 MHz, $CDCl_3$) δ 167.7, 134.9, 131.3, 130.0, 129.8, 128.5, 126.8, 40.1, 31.9, 29.8, 29.7, 29.7, 29.5, 29.4, 29.3, 29.2, 27.2, 27.1, 27.0, 22.7, 14.0.

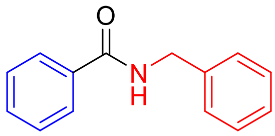

3o ***N*-benzylbenzamide[19]:** The title compound was prepared according to the general procedure and purified by column chromatography using petroleum ether/diethyl ether (5:1) to give the desired product; ^{1}**H NMR** (400 MHz, $CDCl_3$) δ 7.84-7.74 (m, 2H), 7.53-7.45 (m, 1H), 7.42 (m, 2H), 7.36-7.29 (m, 5H), 6.49 (brs, 1H), 4.64 (d, *J* = 5.7 Hz, 2H); ^{13}**C NMR** (100 MHz, $CDCl_3$) δ 167.3, 138.2, 134.3, 131.5, 128.7, 128.5, 127.8, 127.5, 126.9, 44.1.

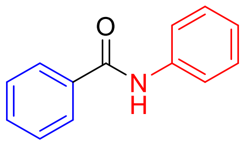

3p ***N*-phenylbenzamide[19]:** The title compound was prepared according to the general procedure and purified by column chromatography using petroleum ether/diethyl ether (5:1) to give the desired product; **^{1}H NMR** (400 MHz, $CDCl_3$) δ 7.90-7.81 (m, 2H), 7.68-7.62 (m, 2H), 7.59-7.52 (m, 1H), 7.52-7.44 (m, 2H), 7.40-7.36 (m, 2H), 7.18-7.14 (m, 1H); **^{13}C NMR** (100 MHz, $CDCl_3$) δ 165.7, 137.9, 134.9, 131.8, 129.1, 128.8, 127.0, 124.5, 130.2.

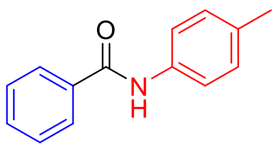

3q ***N*-(*p*-tolyl)benzamide[17]:** The title compound was prepared according to the general procedure and purified by column chromatography using petroleum ether/diethyl ether (5:1) to give the desired product; **^{1}H NMR** (400 MHz, $CDCl_3$) δ 7.88-.78 (m, 2H), 7.57-7.47 (m, 3H), 7.46-7.38 (m, 2H), 7.16-7.14 (m, 2H), 2.33 (s, 3H); **^{13}C NMR** (100 MHz, $CDCl_3$) δ 165.7, 135.3, 135.0, 134.2, 131.7, 129.5, 128.7, 127.0, 120.3, 20.9.

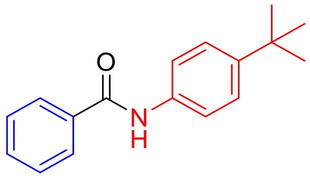

3r ***N*-(4-(*tert*-butyl)phenyl)benzamide[19]:** The title compound was prepared according to the general procedure and purified by column chromatography using petroleum ether/diethyl ether (5:1) to give the desired product; **^{1}H NMR** (400 MHz, $CDCl_3$) δ 7.90-7.83 (m, 2H), 7.59-7.51 (m, 3H), 7.49-7.45 (m, 2H), 7.39-7.37 (m, 2H), 1.32 (s, 9H); **^{13}C NMR** (100 MHz, $CDCl_3$) δ 165.7, 147.6, 135.3, 135.1, 131.7, 128.8, 127.0, 125.9, 120.0, 34.4, 31.4.

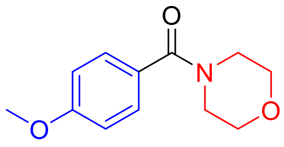

3s **(4-methoxyphenyl)(morpholino)methanone[11]:** The title compound was prepared according to the general procedure and purified by column chromatography using petroleum ether/diethyl ether (5:1) to give the desired product; **^{1}H NMR** (400 MHz, $CDCl_3$) δ 7.40-7.30 (m, 2H), 6.93-6.91 (m, 2H), 3.83 (s, 3H), 3.69-3.62 (m, 8H); **^{13}C NMR** (100 MHz, $CDCl_3$) δ 170.2, 160.7, 129.0, 127.2, 113.6, 66.8, 55.2.

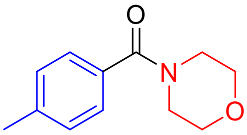 **3t morpholino(*p*-tolyl)methanone[11]:** The title compound was prepared according to the general procedure and purified by column chromatography using petroleum ether/diethyl ether (5:1) to give the desired product; **^{1}H NMR** (400 MHz, $CDCl_3$) δ 7.30-7.27 (m, 2H), 7.21-7.19 (m, 2H), 3.69-3.50 (m, 8H), 2.36 (s, 3H); **^{13}C NMR** (100 MHz, $CDCl_3$) δ 170.5, 140.0, 132.2, 129.0, 127.1, 66.8, 48.2, 42.6, 21.3.

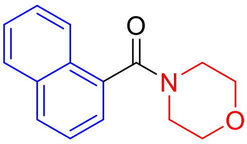 **3u morpholino(naphthalen-1-yl)methanone[20]:** The title compound was prepared according to the general procedure and purified by column chromatography using petroleum ether/diethyl ether (5:1) to give the desired product; **^{1}H NMR** (400 MHz, $CDCl_3$) δ 7.91-7.84 (m, 3H), 7.59-7.39 (m, 4H), 4.03-3.76 (m, 4H), 3.58-3.41 (m, 2H), 3.20-3.16 (m, 2H); **^{13}C NMR** (100 MHz, $CDCl_3$) δ 169.2, 133.5, 133.3, 129.3, 129.1, 128.3, 126.9, 126.3, 125.0, 124.4, 123.7, 47.4, 42.0.

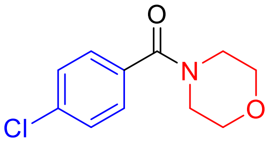 **3w (4-chlorophenyl)(morpholino)methanone[11]:** The title compound was prepared according to the general procedure and purified by column chromatography using petroleum ether/diethyl ether (5:1) to give the desired product; **^{1}H NMR** (400 MHz, $CDCl_3$) δ 7.41-7.41 (m, 4H), 3.72-3.45 (m, 8H); **^{13}C NMR** (100 MHz, $CDCl_3$) δ 170.3, 135.2, 129.8, 128.4, 127.0, 66.8, 48.1, 42.4.

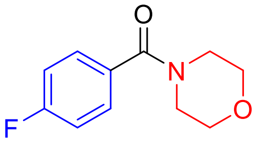 **3x (4-fluorophenyl)(morpholino)methanone[11]:** The title compound was prepared according to the general procedure and purified by column chromatography using petroleum ether/diethyl ether (5:1) to give the desired product; **^{1}H NMR** (400 MHz, $CDCl_3$) δ 7.44-7.41 (m, 2H), 7.13-7.08 (m, 2H), 3.70-3.47 (m, 8H); **^{13}C NMR** (100 MHz, $CDCl_3$) δ 169.4, 163.4 (d, J = 250.1 Hz), 131.22 (d, J = 3.5 Hz), 129.4 (d, J = 8.5 Hz), 115.6 (d, J = 21.9 Hz), 48.2, 42.7.

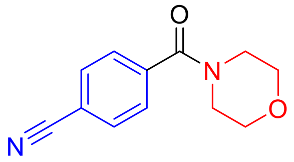

3y 4-(morpholine-4-carbonyl)benzonitrile[20]: The title compound was prepared according to the general procedure and purified by column chromatography using petroleum ether/diethyl ether (5:1) to give the desired product; **^{1}H NMR** (400 MHz, $CDCl_3$) δ 7.62-7.44 (m, 2H), 6.92-6.80 (m, 2H), 3.91-3.78 (m, 4H), 3.29-3.27 (m, 4H); **^{13}C NMR** (100 MHz, $CDCl_3$) δ 153.5, 133.5, 128.5, 127.0, 119.8, 114.0, 100.9, 66.4, 47.3.

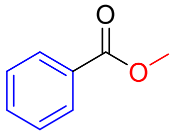

5a methyl benzoate[19]: The title compound was prepared according to the general procedure and purified by column chromatography using petroleum ether/diethyl ether (50:1) to give the desired product; **^{1}H NMR** (400 MHz, $CDCl_3$) δ 8.18-7.90 (m, 2H), 7.61-7.49 (m, 1H), 7.45-7.42 (m, 2H), 3.92 (s, 3H); **^{13}C NMR** (100 MHz, $CDCl_3$) δ 167.1, 132.9, 130.2, 129.5, 128.3, 52.0.

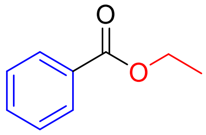

5b ethyl benzoate[19]: The title compound was prepared according to the general procedure and purified by column chromatography using petroleum ether/diethyl ether (50:1) to give the desired product; **^{1}H NMR** (400 MHz, $CDCl_3$) δ 8.10-8.01 (m, 2H), 7.60-7.49 (m, 1H), 7.49-7.38 (m, 2H), 4.38 (q, J = 7.1 Hz, 2H), 1.40 (t, J = 7.1 Hz, 3H); **^{13}C NMR** (100 MHz, $CDCl_3$) δ 166.6, 132.8, 130.5, 129.5, 128.3, 60.9, 14.3.

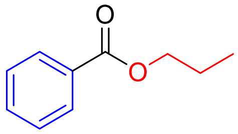

5c propyl benzoate[21]: The title compound was prepared according to the general procedure and purified by column chromatography using petroleum ether/diethyl ether (50:1) to give the desired product; **^{1}H NMR** (400 MHz, $CDCl_3$) δ 8.10-8.00 (m, 2H), 7.60-7.51 (m, 1H), 7.49- 7.38 (m, 2H), 4.29 (t, J = 6.7 Hz, 2H), 1.80 (dtd, J = 14.0, 7.4, 6.6 Hz, 2H), 1.04 (t, J = 7.4 Hz, 3H); **^{13}C NMR** (100 MHz, $CDCl_3$) δ 166.7, 132.8, 130.5, 129.5, 128.3, 66.5, 22.1, 10.5.

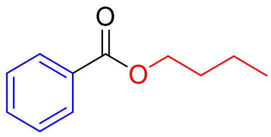

5d butyl benzoate[21]**:** The title compound was prepared according to the general procedure and purified by column chromatography using petroleum ether/diethyl ether (20:1 to 2:1) to give the desired product which was a white solid; mp: 68-70°C; **^{1}H NMR** (400 MHz, $CDCl_3$) δ 8.07-8.02 (m, 2H), 7.53 (t, *J* = 7.4 Hz, 1H), 7.42 (t, *J* = 7.6 Hz, 2H), 4.32 (t, *J* = 6.6 Hz, 2H), 1.82-1.68 (m, 2H), 1.48 (h, *J* = 7.4 Hz, 2H), 0.98 (t, *J* = 7.4 Hz, 3H); **^{13}C NMR** (100 MHz, $CDCl_3$) δ 166.7, 132.8, 130.5, 129.5, 128.3, 66.5, 22.1, 10.5.

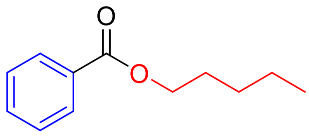

5e pentyl benzoate[22]**:** The title compound was prepared according to the general procedure and yield was determined by ^{1}HNMR with triphenylmethane as internal standard; **^{1}H NMR** (400 MHz, $CDCl_3$) δ 5.56 (s, 0.5H), 4.32 (t, *J* = 6.7 Hz, 1.7H).

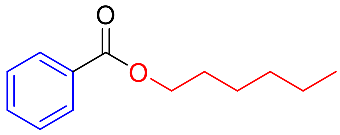

5f hexyl benzoate[22]**:** The title compound was prepared according to the general procedure and yield was determined by ^{1}HNMR with triphenylmethane as internal standard; **^{1}H NMR** (400 MHz, $CDCl_3$) δ 5.55 (s, 0.5H), 4.32 (t, *J* = 6.7 Hz, 1.8H).

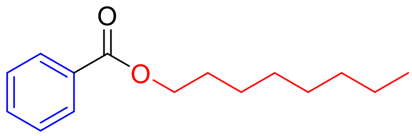

5g octyl benzoate[22]**:** The title compound was prepared according to the general procedure and yield was determined by ^{1}HNMR with triphenylmethane as internal standard; **^{1}H NMR** (400 MHz, $CDCl_3$) δ 5.54 (s, 0.5H), 4.31 (t, *J* = 6.7 Hz, 1.6H).

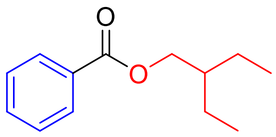

5i 2-ethylbutyl benzoate[23]**:** The title compound was prepared according to the general procedure and purified by column chromatography using petroleum ether/diethyl ether (50:1) to give the desired product; **^{1}H NMR** (400 MHz, $CDCl_3$) δ 8.05-8.04 (m, 2H), 7.55-7.40 (m, 3H), 4.26

(d, J = 5.7 Hz, 2H), 1.66 (p, J = 6.2 Hz, 1H), 1.52-1.38 (m, 4H), 0.95 (t, J = 7.5 Hz, 6H); **^{13}C NMR** (100 MHz, $CDCl_3$) δ 166.6, 132.7, 130.5, 129.4, 128.2, 66.8, 40.4, 23.4, 11.0.

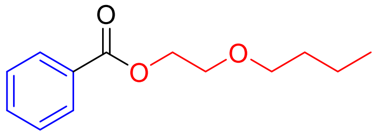

5j 2-butoxyethyl benzoate[24]: The title compound was prepared according to the general procedure and purified by column chromatography using petroleum ether/diethyl ether (50:1) to give the desired product; **^{1}H NMR** (400 MHz, $CDCl_3$) δ 8.05-8.04 (m, 2H), 7.55-7.40 (m, 3H), 4.26 (d, J = 5.7 Hz, 2H), 1.66 (p, J = 6.2 Hz, 1H), 1.52-1.38 (m, 4H), 0.95 (t, J = 7.5 Hz, 6H); **^{13}C NMR** (100 MHz, $CDCl_3$) δ 166.6, 132.9, 130.1, 129.6, 128.3, 71.2, 68.6, 64.2, 31.7, 19.2, 13.9.

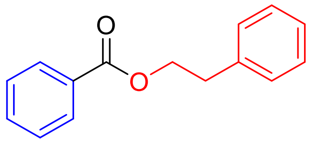

5k phenethyl benzoate[21]: The title compound was prepared according to the general procedure and purified by column chromatography using petroleum ether/diethyl ether (50:1) to give the desired product; **^{1}H NMR** (400 MHz, $CDCl_3$) δ 8.02-8.01 (m, 2H), 7.57-7.53 (m, 1H), 7.45-7.41 (m, 2H), 7.37 – 7.19 (m, 5H), 4.53 (t, J = 7.0 Hz, 2H), 3.08 (t, J = 7.0 Hz, 2H); **^{13}C NMR** (100 MHz, $CDCl_3$) δ 166.5, 137.9, 132.9, 130.3, 129.5, 128.9, 128.5, 128.3, 126.6, 65.5, 35.2.

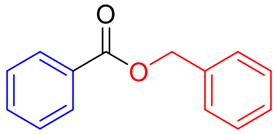

5l benzyl benzoate[21]: The title compound was prepared according to the general procedure and yield was determined by ^{1}HNMR with triphenylmethane as internal standard; **^{1}H NMR** (400 MHz, $CDCl_3$) δ 5.52 (s, 0.5H), 5.32 (s, 1.41H).

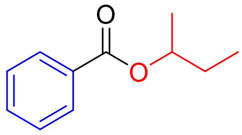

5m *sec*-butyl benzoate[23]: The title compound was prepared according to the general procedure and purified by column chromatography using petroleum ether/diethyl ether (50:1) to give the desired product; **^{1}H NMR** (400 MHz, $CDCl_3$) δ 8.06-8.04 (m, 2H), 7.56-7.41 (m, 3H), 5.10 (h, J = 6.3 Hz, 1H), 1.86-1.57 (m, 2H), 1.34 (d, J = 6.2 Hz, 3H), 0.98 (t, J = 7.4 Hz, 3H); **^{13}C NMR** (100 MHz, $CDCl_3$) δ 166.2, 132.6, 130.9, 129.5, 128.2, 28.9, 19.5, 9.7.

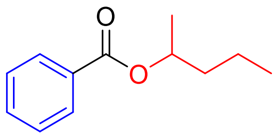

5n pentan-2-yl benzoate[23]: The title compound was prepared according to the general procedure and purified by column chromatography using petroleum ether/diethyl ether (50:1) to give the desired product; **^{1}H NMR** (400 MHz, $CDCl_3$) δ 8.05-8.04 (m, 2H), 7.55-7.41 (m, 3H), 5.34-4.94 (m, 1H), 1.75-1.71 (m, 2H), 1.69-1.43 (m, 2H), 1.34 (d, *J* = 6.3 Hz, 3H), 0.94 (t, *J* = 7.3 Hz, 3H); **^{13}C NMR** (100 MHz, $CDCl_3$) δ 166.2, 132.6, 130.9, 129.5, 128.2, 71.4, 38.2, 20.0, 18.7, 13.9.

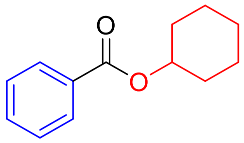

5o cyclohexyl benzoate[21]: The title compound was prepared according to the general procedure and yield was determined by ^{1}HNMR with triphenylmethane as internal standard; **^{1}H NMR** (400 MHz, $CDCl_3$) δ 5.55 (s, 0.5H), 5.03 (tt, *J* = 8.6, 3.8 Hz, 0.93H).

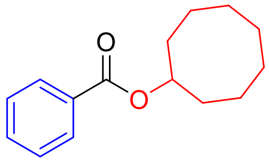

5p cyclooctyl benzoate[25]: The title compound was prepared according to the general procedure and purified by column chromatography using petroleum ether/diethyl ether (50:1) to give the desired product; **^{1}H NMR** (400 MHz, $CDCl_3$) δ 8.04-8.03 (m, 2H), 7.54-7.52 (m, 1H), 7.50-7.39 (m, 2H), 5.20 (dt, *J* = 8.2, 4.0 Hz, 1H), 1.98-1.83 (m, 2H), 1.81-1.72 (m, 2H), 1.69-1.46 (m, 10H); **^{13}C NMR** (100 MHz, $CDCl_3$) δ 165.8, 132.5, 131.0, 129.4, 128.2, 75.5, 63.2, 31.7, 31.4, 27.1, 25.3, 23.2, 22.9.

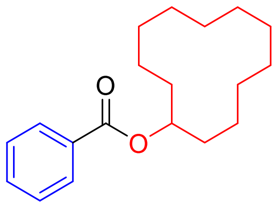

5q cyclododecyl benzoate[25]: The title compound was prepared according to the general procedure and purified by column chromatography using petroleum ether/diethyl ether (50:1) to give the desired product; **^{1}H NMR** (400 MHz, $CDCl_3$) δ 8.09-8.00 (m, 2H), 7.54-7.51 (m, 1H), 7.44-7.40 (m, 2H), 5.26 (tt, *J* = 7.2, 4.6 Hz, 1H), 3.53-3.32 (m, 1H), 1.86-1.79 (m, 2H), 1.70-1.59 (m, 2H),

1.53-1.26 (m, 17H); **^{13}C NMR** (100 MHz, $CDCl_3$) δ 166.1, 132.6, 130.9, 129.4, 128.2, 72.8, 63.5, 29.1, 28.9, 24.6, 24.0, 23.9, 23.3, 23.1, 20.8, 20.7, 15.7.

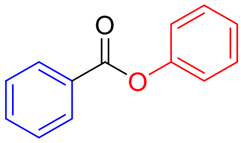

5r phenyl benzoate[19]: The title compound was prepared according to the general procedure and purified by column chromatography using petroleum ether/diethyl ether (50:1) to give the desired product; **^{1}H NMR** (400 MHz, $CDCl_3$) δ 8.23-8.17 (m, 2H), 7.66-7.59 (m, 1H), 7.50 (t, J = 7.6 Hz, 2H), 7.42 (t, J = 7.8 Hz, 2H), 7.29-7.16 (m, 3H); **^{13}C NMR** (100 MHz, $CDCl_3$) δ 165.2, 150.1, 133.5, 130.2, 129.6, 129.5, 129.5, 128.6, 125.9, 121.7.

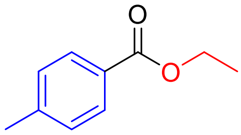

5s ethyl 4-methylbenzoate[26]: The title compound was prepared according to the general procedure and purified by column chromatography using petroleum ether/diethyl ether (50:1) to give the desired product; **^{1}H NMR** (400 MHz, $CDCl_3$) δ 7.93 (d, J = 8.3 Hz, 2H), 7.22 (d, J = 7.9 Hz, 2H), 4.36 (q, J = 7.1 Hz, 2H), 2.40 (s, 3H), 1.38 (t, J = 7.2 Hz, 3H); **^{13}C NMR** (100 MHz, $CDCl_3$) δ 166.6, 143.3, 129.5, 129.0, 127.7, 60.7, 21.6, 14.3.

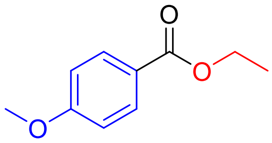

5t ethyl 4-methoxybenzoate[26]: The title compound was prepared according to the general procedure and purified by column chromatography using petroleum ether/diethyl ether (50:1) to give the desired product; **^{1}H NMR** (400 MHz, $CDCl_3$) δ 8.05-7.94 (m, 2H), 6.96-6.85 (m, 2H), 4.34 (q, J = 7.1 Hz, 2H), 3.84 (s, 3H), 1.37 (t, J = 7.1 Hz, 3H); **^{13}C NMR** (100 MHz, $CDCl_3$) δ 166.3, 163.2, 131.4, 122.7, 113.5, 60.5, 55.3, 14.3.

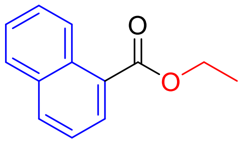

5u ethyl 1-naphthoate[27]: The title compound was prepared according to the general procedure and purified by column chromatography using petroleum ether/diethyl ether (50:1) to give the desired product; **^{1}H NMR** (400 MHz, $CDCl_3$) δ 8.92 (d, J = 8.5 Hz, 1H), 8.16 (dd, J = 7.2, 1.4 Hz, 1H), 7.97 (d, J = 8.2 Hz, 1H), 7.84 (d, J = 8.1 Hz, 1H), 7.59 (t, J = 7.5 Hz, 1H), 7.52-7.35 (m, 2H),

4.46 (q, J = 7.1 Hz, 2H), 1.43 (t, J = 7.1 Hz, 3H); **^{13}C NMR** (100 MHz, $CDCl_3$) δ 167.5, 133.8, 133.1, 131.3, 130.0, 128.4, 127.6, 127.4, 126.1, 125.7, 124.4, 61.0, 14.3.

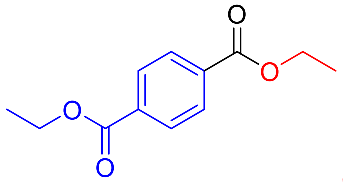

5w diethyl terephthalate[21]: The title compound was prepared according to the general procedure and purified by column chromatography using petroleum ether/diethyl ether (50:1) to give the desired product; **^{1}H NMR** (400 MHz, $CDCl_3$) δ 8.10 (s, 4H), 4.41 (q, J = 7.1 Hz, 4H), 1.41 (t, J = 7.1 Hz, 5H); **^{13}C NMR** (100 MHz, $CDCl_3$) δ 165.8, 134.1, 129.4, 61.3, 14.2.

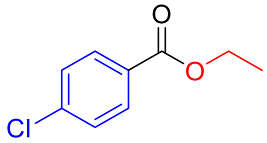

5x ethyl 4-chlorobenzoate[21]: The title compound was prepared according to the general procedure and purified by column chromatography using petroleum ether/diethyl ether (50:1) to give the desired product; **^{1}H NMR** (400 MHz, $CDCl_3$) δ 7.98 (d, J = 8.5 Hz, 2H), 7.49-7.31 (m, 2H), 4.37 (q, J = 7.1 Hz, 2H), 1.39 (t, J = 7.1 Hz, 3H); **^{13}C NMR** (100 MHz, $CDCl_3$) δ 166.7, 139.2, 132.7, 130.9, 129.5, 128.6, 61.1, 14.2.

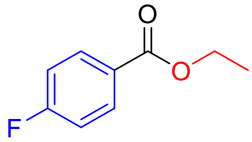

5y ethyl 4-fluorobenzoate[21]: The title compound was prepared according to the general procedure and purified by column chromatography using petroleum ether/diethyl ether (50:1) to give the desired product; **^{1}H NMR** (400 MHz, $CDCl_3$) δ 8.08-8.04 (m, 2H), 7.27-7.08 (m, 2H), 4.37 (q, J = 7.1 Hz, 2H), 1.39 (t, J = 7.1 Hz, 3H); **^{13}C NMR** (100 MHz, $CDCl_3$) δ 166.9, 164.98 (d, J = 121.7 Hz), 132.00 (d, J = 9.3 Hz), 129.5, 129.0, 126.70 (d, J = 3.0 Hz), 115.36 (d, J = 21.9 Hz), 61.0, 14.2.

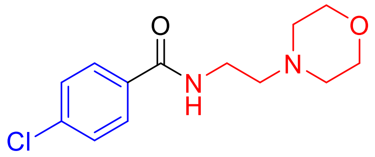

6a Moclobemide[11]: The title compound was prepared according to the general procedure and purified by column chromatography using petroleum ether/diethyl ether (50:1) to give the desired product; **^{1}H NMR** (400 MHz, $CDCl_3$) δ 7.70 (d, J = 8.3 Hz, 2H), 7.39 (d, J = 8.1 Hz,

2H), 6.82 (brs, 1H), 3.70 (t, *J* = 4.6 Hz, 4H), 3.52 (q, *J* = 5.6 Hz, 2H), 2.58 (t, *J* = 6.0 Hz, 2H), 2.49 (d, *J* = 4.8 Hz, 4H); **^{13}C NMR** (100 MHz, $CDCl_3$) δ 166.3, 137.5, 132.7, 128.7, 128.3, 66.9, 56.8, 53.3, 36.0.

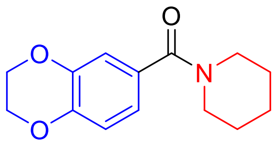

6b CX-546[11]: The title compound was prepared according to the general procedure and purified by column chromatography using petroleum ether/diethyl ether (50:1) to give the desired product; **^{1}H NMR** (400 MHz, $CDCl_3$) δ 7.06-6.71 (m, 3H), 4.26 (s, 4H), 3.61-3.62 (m, 4H), 1.85-1.16 (m, 6H); **^{13}C NMR** (100 MHz, $CDCl_3$) δ 169.7, 144.6, 143.2, 129.6, 120.4, 117.1, 115.4, 64.4, 64.3, 24.6.

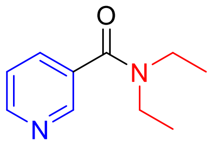

6c Nikethamide[11]: The title compound was prepared according to the general procedure and purified by column chromatography using petroleum ether/diethyl ether (50:1) to give the desired product; **^{1}H NMR** (400 MHz, $CDCl_3$) δ 8.66 (s, 2H), 7.73 (d, *J* = 7.7 Hz, 1H), 7.40-7.23 (m, 1H), 3.57 (d, *J* = 7.9 Hz, 2H), 3.28 (d, *J* = 7.9 Hz, 2H), 1.25 (t, *J* = 7.1 Hz, 3H), 1.15 (t, *J* = 7.1 Hz, 3H); **^{13}C NMR** (100 MHz, $CDCl_3$) δ 166.3, 137.5, 132.7, 128.7, 128.3, 66.9, 56.8, 53.3, 36.0.

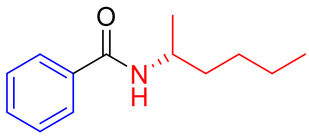

6d *(R)*-N-(hexan-2-yl)benzamide[28]: The title compound was prepared according to the general procedure and purified by column chromatography using petroleum ether/diethyl ether (5:1) to give the desired product; **^{1}H NMR** (400 MHz, $CDCl_3$) δ 7.83-7.69 (m, 2H), 7.51-7.45 (m, 1H), 7.43-7.39 (m, 2H), 5.91 (s, 1H), 4.18 (dq, *J* = 8.4, 6.5 Hz, 1H), 1.55-1.51 (m, 2H), 1.37-1.31 (m, 4H), 1.23 (d, *J* = 6.6 Hz, 3H), 0.90 (t, *J* = 7.3 Hz, 3H); **^{13}C NMR** (100 MHz, $CDCl_3$) δ 166.8, 135.1, 131.2, 128.5, 126.8, 44.7, 36.8, 28.2, 22.6, 21.0, 14.0.

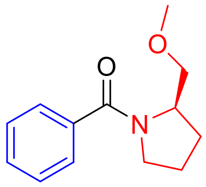

6e *(R)*-(2-(methoxymethyl)pyrrolidin-1-yl)(phenyl)methanone[29]: The title compound was prepared according to the general procedure and purified by column chromatography

using petroleum ether/diethyl ether (5:1) to give the desired product; **^{1}H NMR** (400 MHz, $CDCl_3$) δ 7.72-7.30 (m, 5H), 4.44 (s, 1H), 3.75-3.54 (m, 2H), 3.40 (s, 3H), 2.08-1.90 (m, 4H), 1.76-1.70 (m, 2H); **^{13}C NMR** (100 MHz, $CDCl_3$) δ 170.0, 129.8, 128.2, 127.3, 59.2, 56.7, 50.6, 27.7, 25.1.

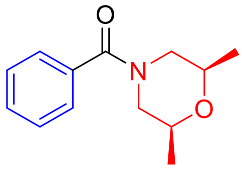

6f ***((2S,6R)*-2,6-dimethylmorpholino)(phenyl)methanone[30]:** The title compound was prepared according to the general procedure and purified by column chromatography using petroleum ether/diethyl ether (5:1) to give the desired product; **^{1}H NMR** (400 MHz, $CDCl_3$) δ 7.41-7.37 (m, 5H), 4.54 (s, 1H), 3.55 (t, J = 23.3 Hz, 3H), 2.78 (s, 1H), 2.53 (s, 1H), 1.23 (s, 3H), 1.08 (s, 3H); **^{13}C NMR** (100 MHz, $CDCl_3$) δ 170.0, 135.5, 129.7, 128.5, 127.0, 71.9, 53.2, 47.5, 18.6.

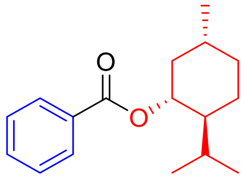

6g ***(1R,2S,5R)*-2-isopropyl-5-methylcyclohexyl benzoate[31]:** The title compound was prepared according to the general procedure and purified by column chromatography using petroleum ether/diethyl ether (5:1) to give the desired product; **^{1}H NMR** (400 MHz, $CDCl_3$) δ 8.05 (d, J = 7.7 Hz, 2H), 7.55 (t, J = 7.4 Hz, 1H), 7.44 (t, J = 7.6 Hz, 2H), 4.94 (td, J = 10.9, 4.4 Hz, 1H), 2.20-2.07 (m, 1H), 2.01-1.93 (m, 1H), 1.76-1.70 (m, 2H), 1.59-1.52 (m, 2H), 1.32-1.01 (m, 3H), 0.93 (dd, J = 6.8, 4.4 Hz, 6H), 0.80 (d, J = 6.9 Hz, 3H); **^{13}C NMR** (100 MHz, $CDCl_3$) δ 166.1, 132.7, 130.9, 129.5, 128.3, 47.3, 41.0, 34.3, 31.4, 26.5, 23.6, 22.0, 20.1, 16.5.

4. NMR spectra

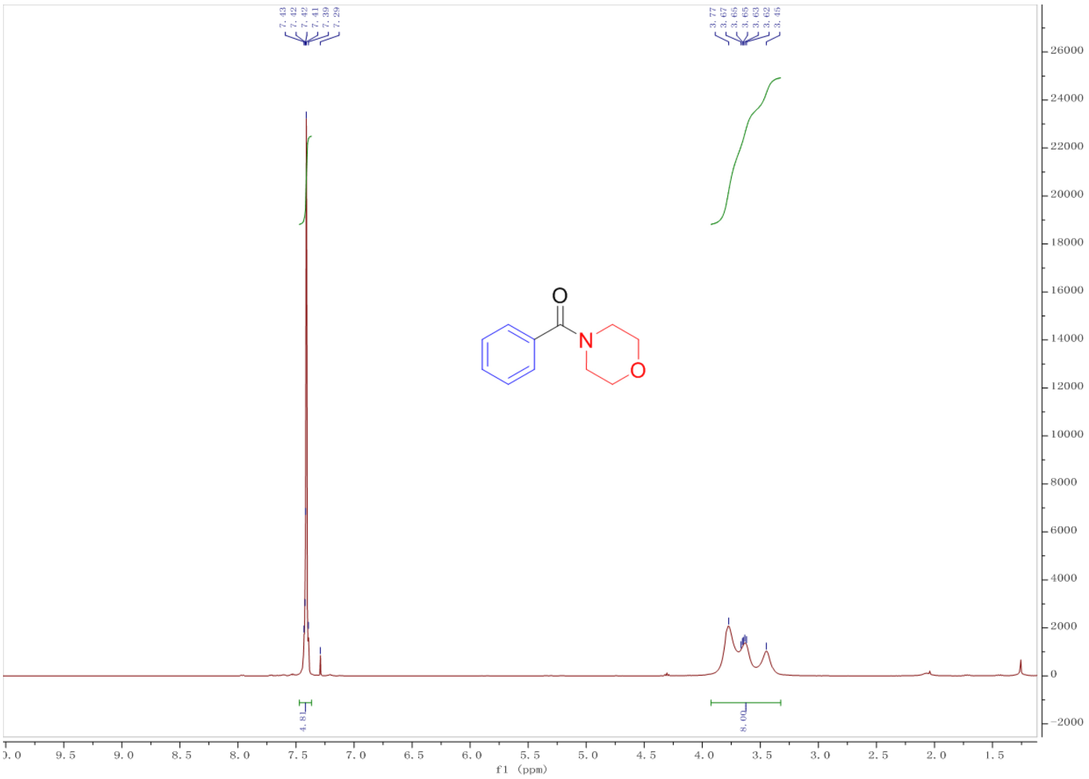

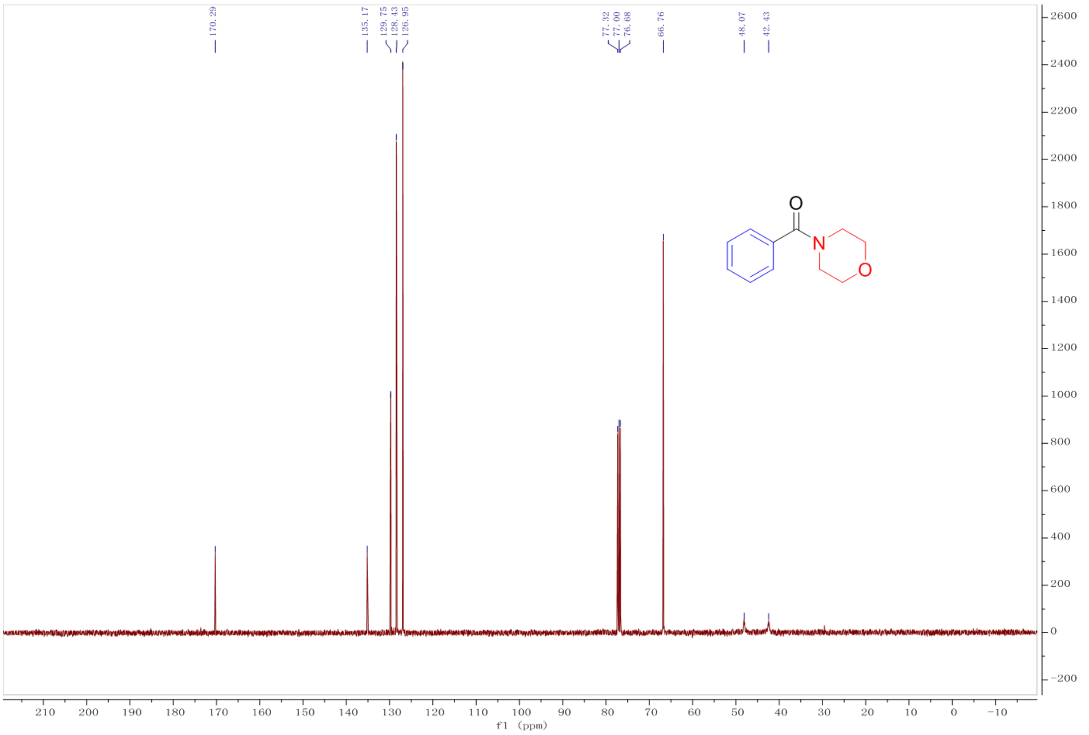

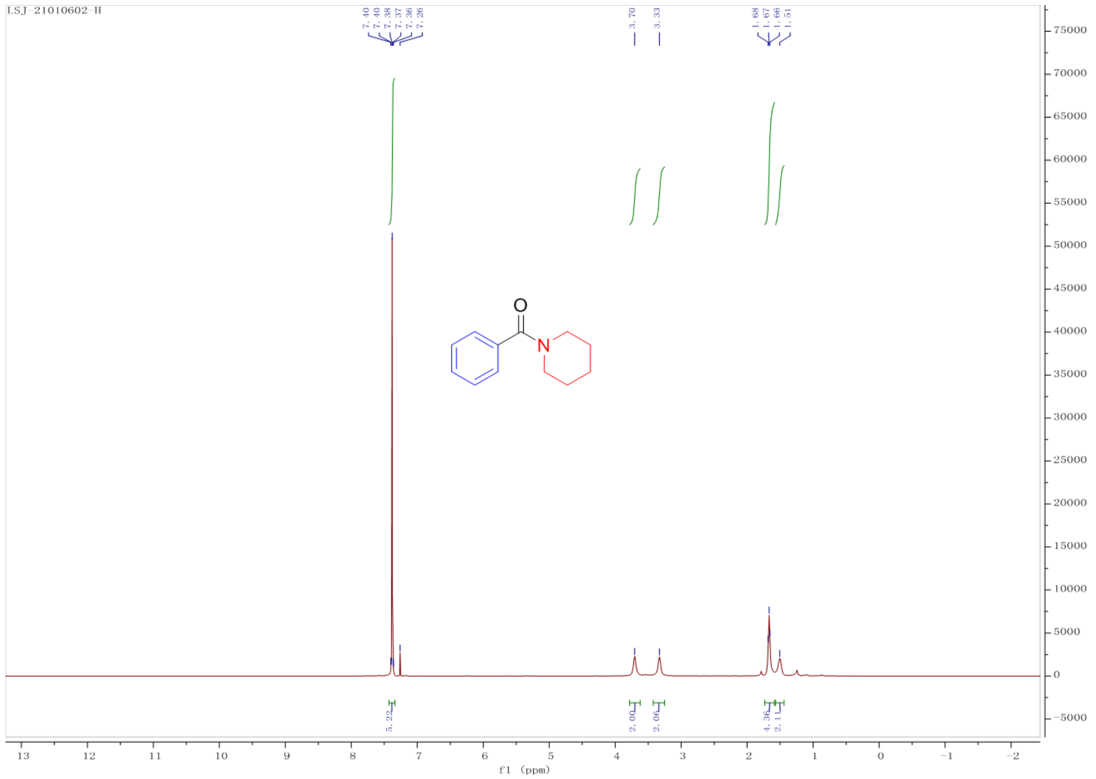
LSJ-21010602-H
7.40
7.40
7.38
7.37
7.36
7.26
3.70
3.33
1.68
1.67
1.66
1.51
5.22
2.00
2.06
4.36
2.1
f1 (ppm)

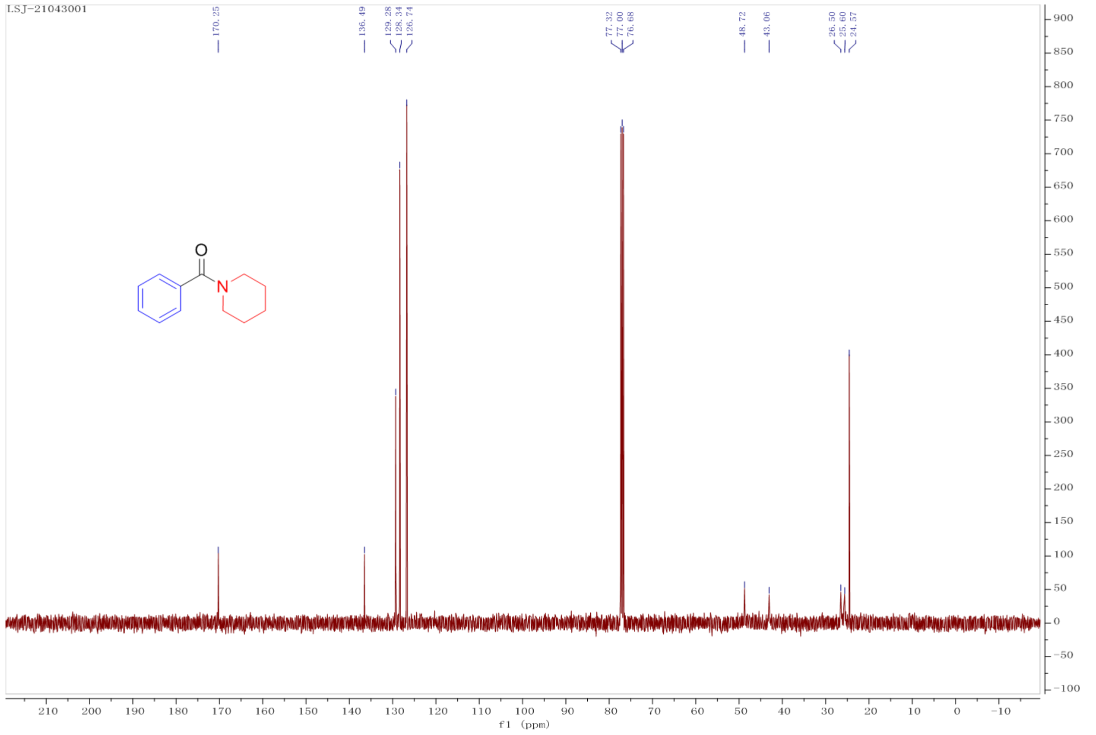
LSJ-21043001
170.25
136.49
129.28
128.34
126.74
77.32
77.00
76.68
48.72
43.06
26.50
25.60
24.57
f1 (ppm)

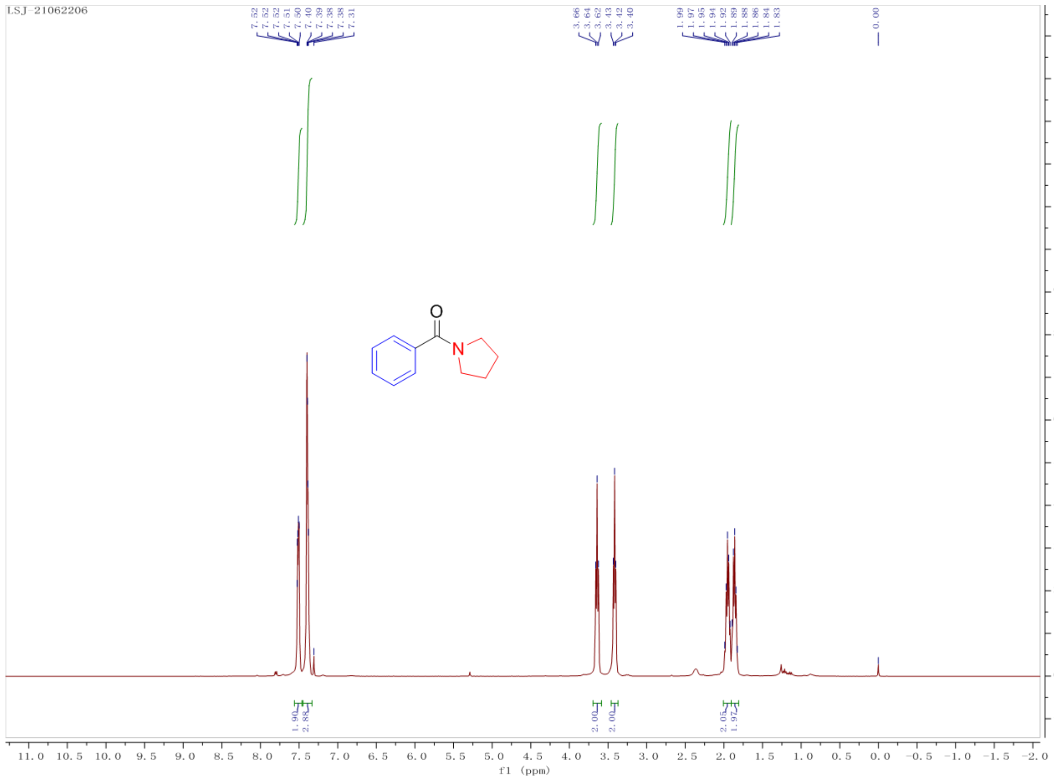
LSJ 21062206
f1 (ppm)

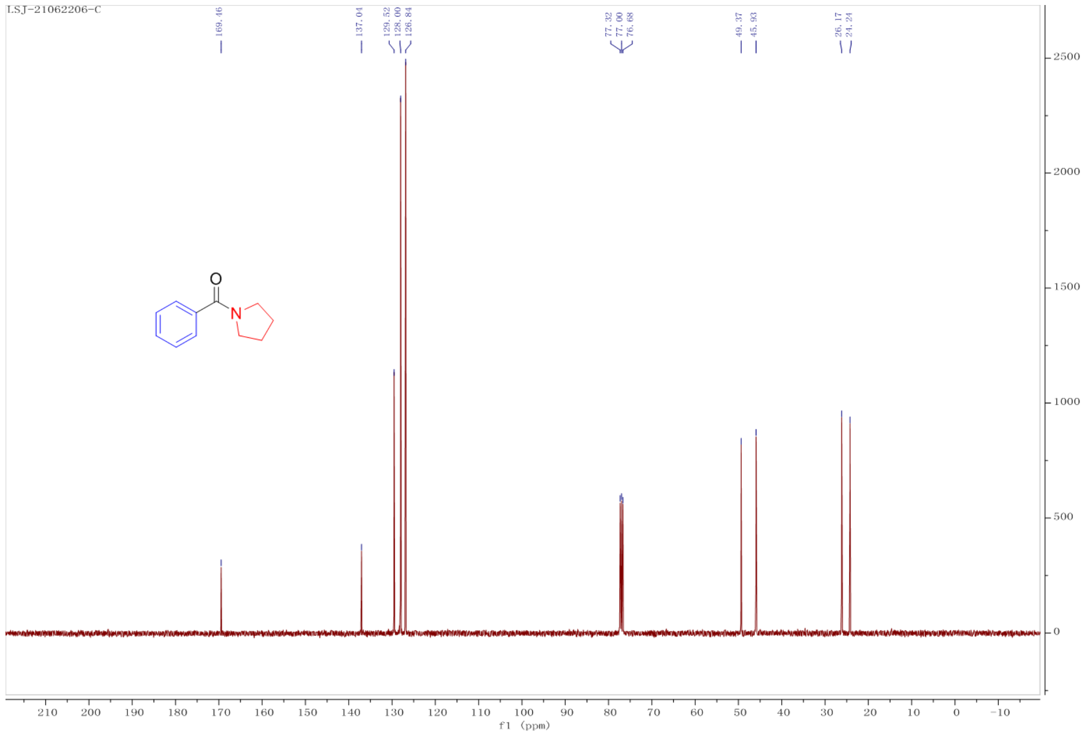
LSJ-21062206-C
f1 (ppm)

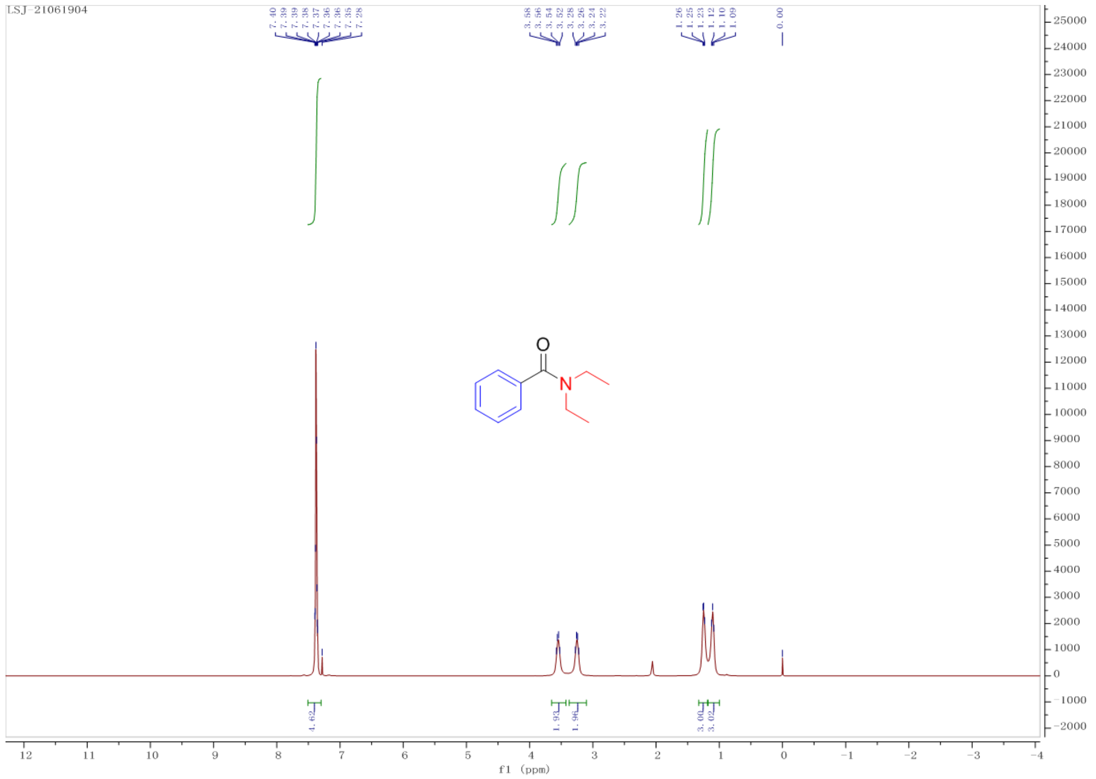
LSJ-21061904
7.40
7.39
7.39
7.38
7.37
7.36
7.36
7.35
7.28
3.58
3.56
3.54
3.52
3.28
3.26
3.24
3.22
1.26
1.25
1.23
1.12
1.10
1.09
0.00
4.62
1.93
1.96
3.00
3.02
O
N
f1 (ppm)

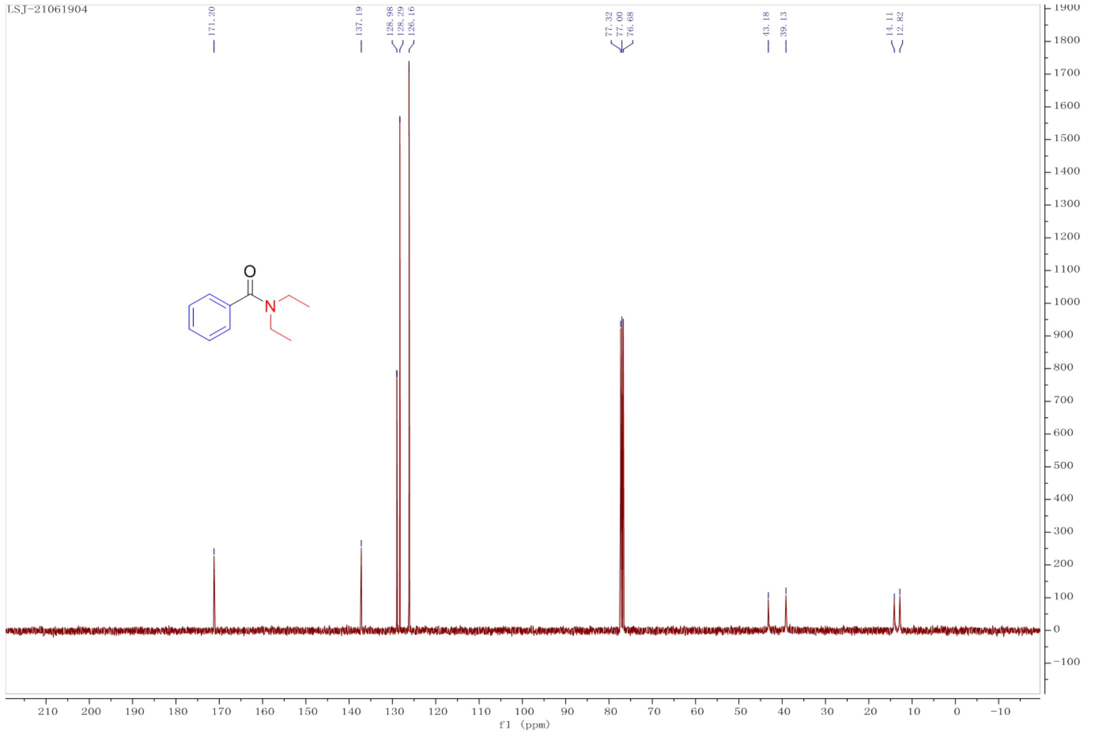
LSJ-21061904
171.20
137.19
128.98
128.29
126.16
77.32
77.00
76.68
43.18
39.13
14.11
12.82
O
N
f1 (ppm)

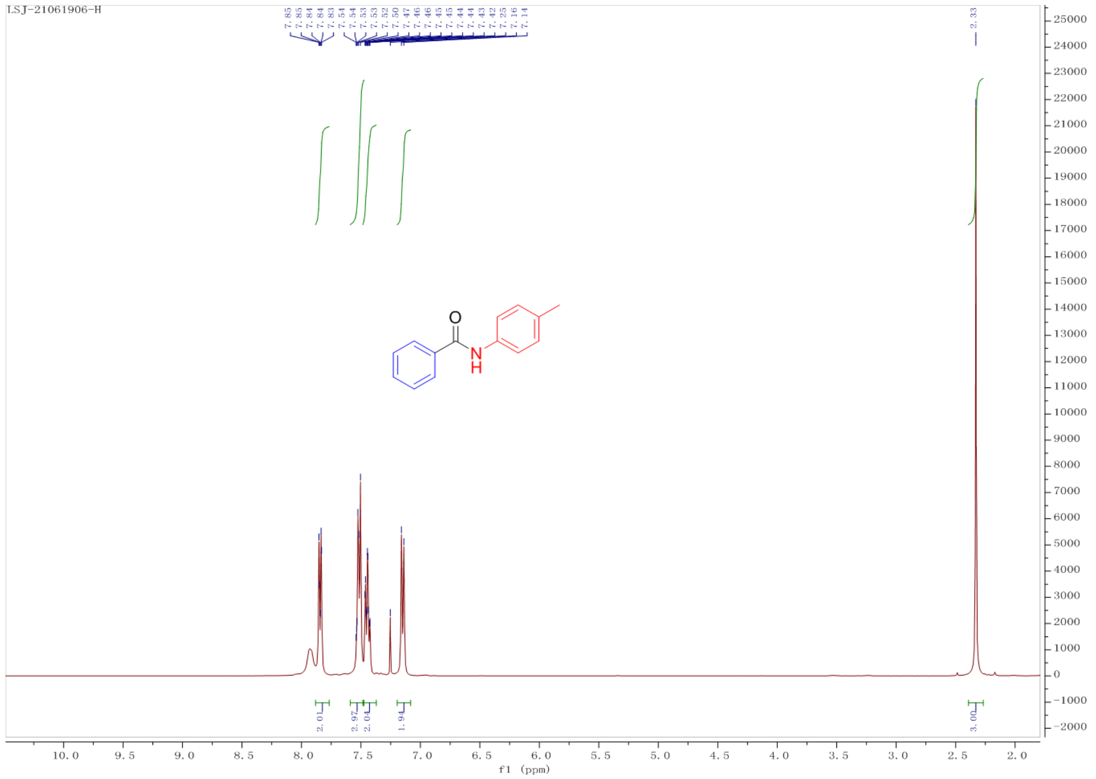
LSJ-21061906-H
7.85
7.85
7.84
7.84
7.83
7.54
7.54
7.53
7.53
7.52
7.50
7.47
7.46
7.46
7.45
7.45
7.44
7.44
7.43
7.42
7.25
7.16
7.14
2.33
O
N
H
2.01
2.97
2.01
1.94
3.00
f1 (ppm)

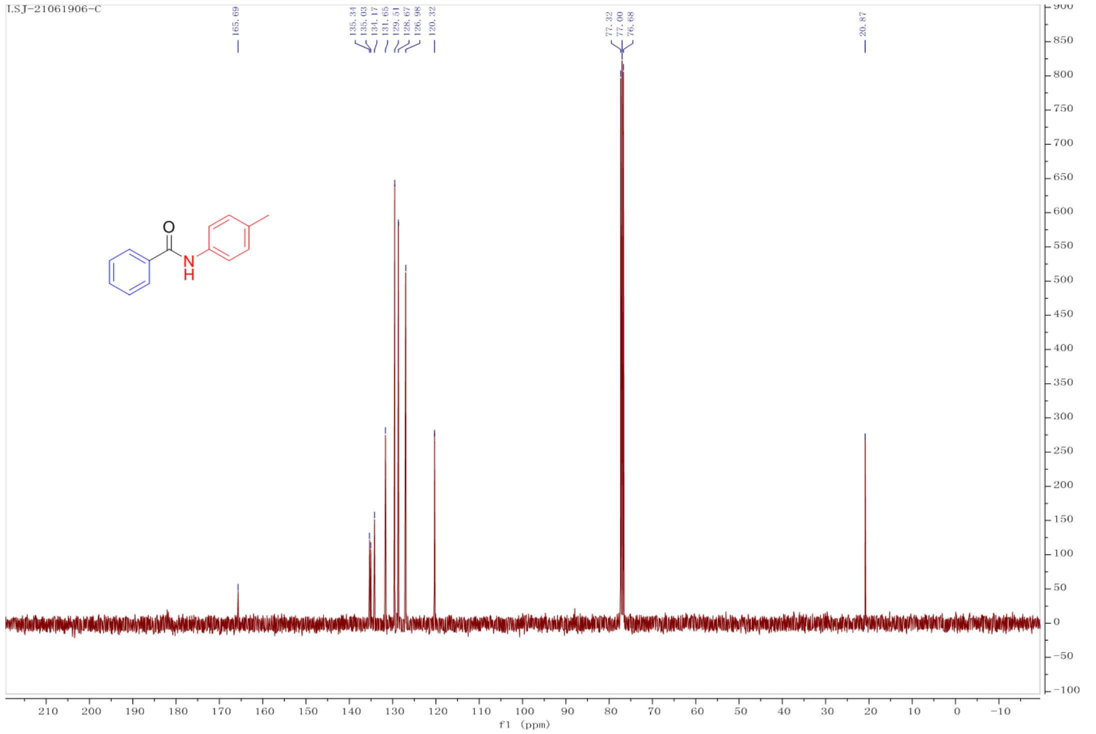
LSJ-21061906-C
165.69
135.34
135.03
134.17
131.65
129.51
128.67
126.98
120.32
77.32
77.00
76.68
20.87
O
N
H
f1 (ppm)

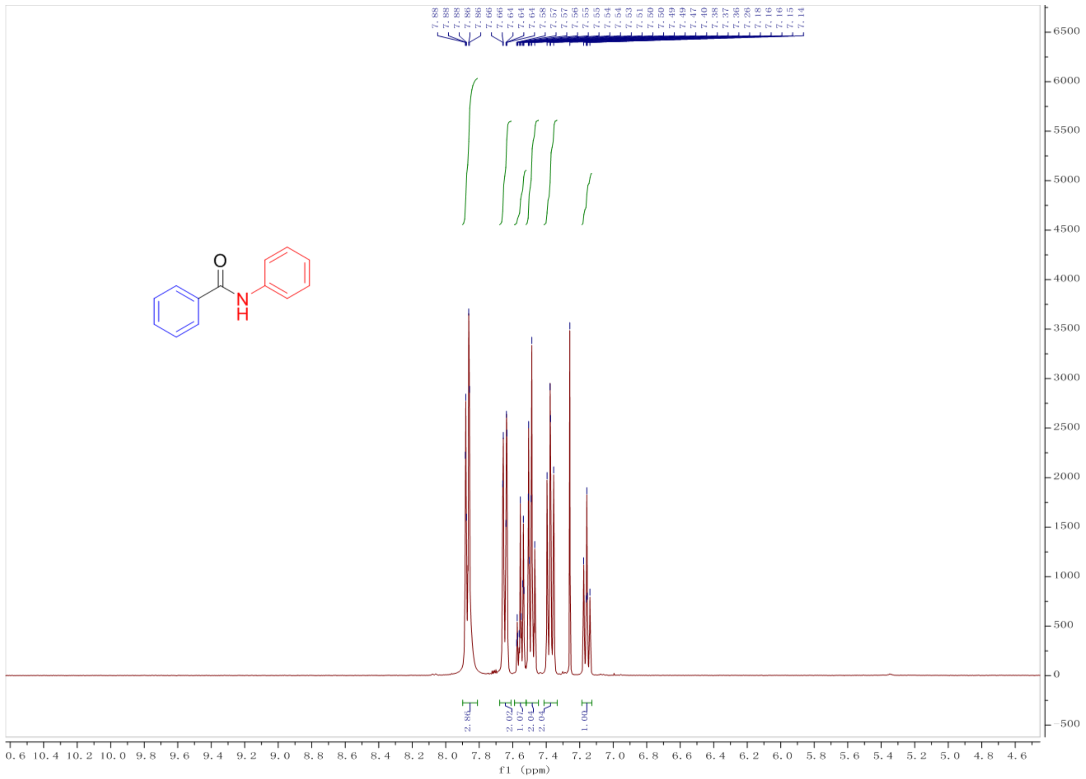
7.88
7.88
7.86
7.86
7.66
7.64
7.64
7.64
7.58
7.57
7.56
7.55
7.55
7.54
7.54
7.53
7.51
7.50
7.50
7.49
7.49
7.47
7.40
7.38
7.37
7.36
7.26
7.18
7.16
7.16
7.15
7.14
2.86
2.02
1.02
2.01
2.04
1.00
f1 (ppm)

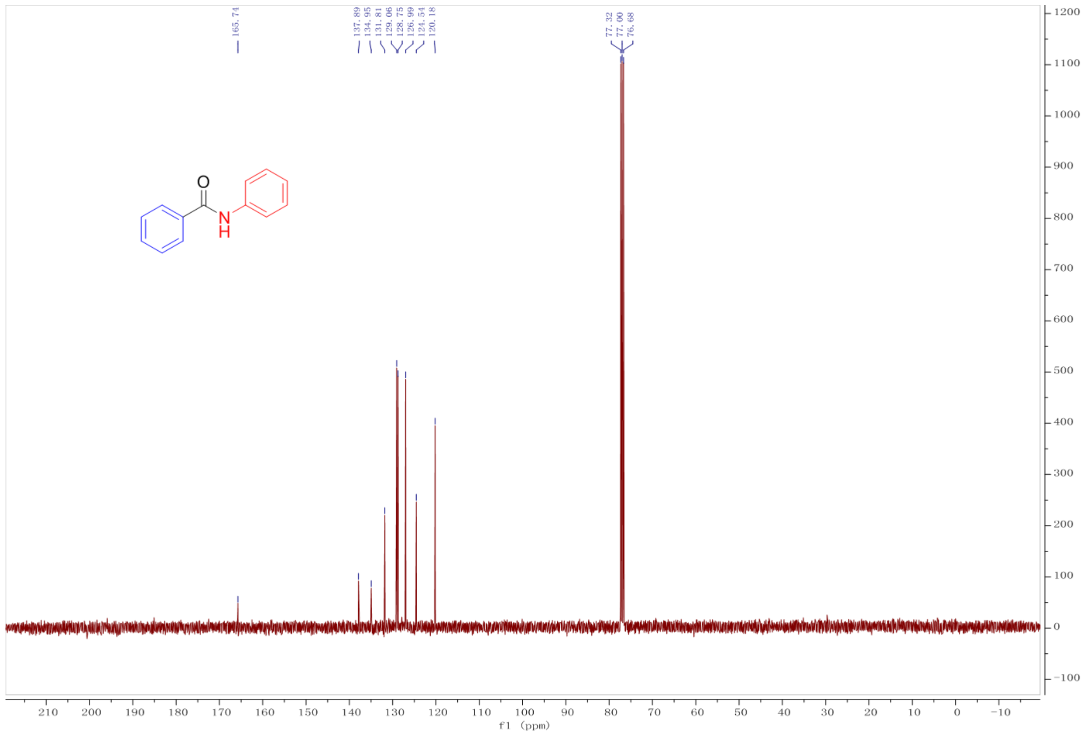
165.74
137.89
134.95
131.81
129.06
128.75
126.99
124.51
120.18
77.32
77.00
76.68
f1 (ppm)

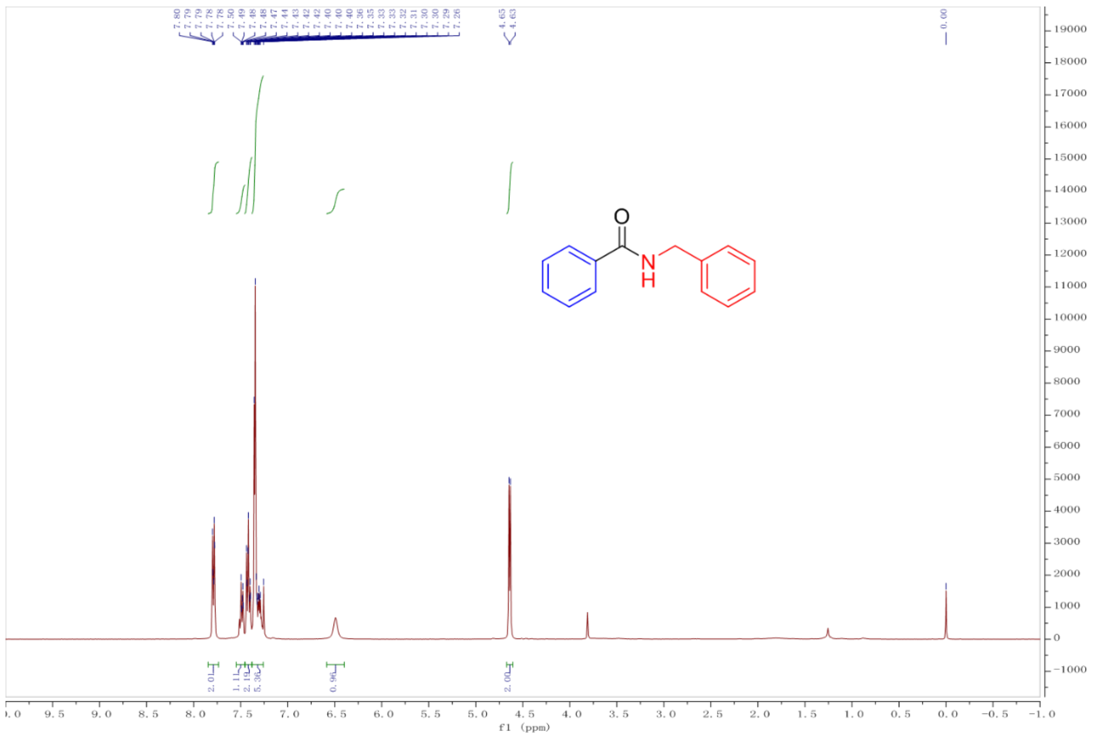

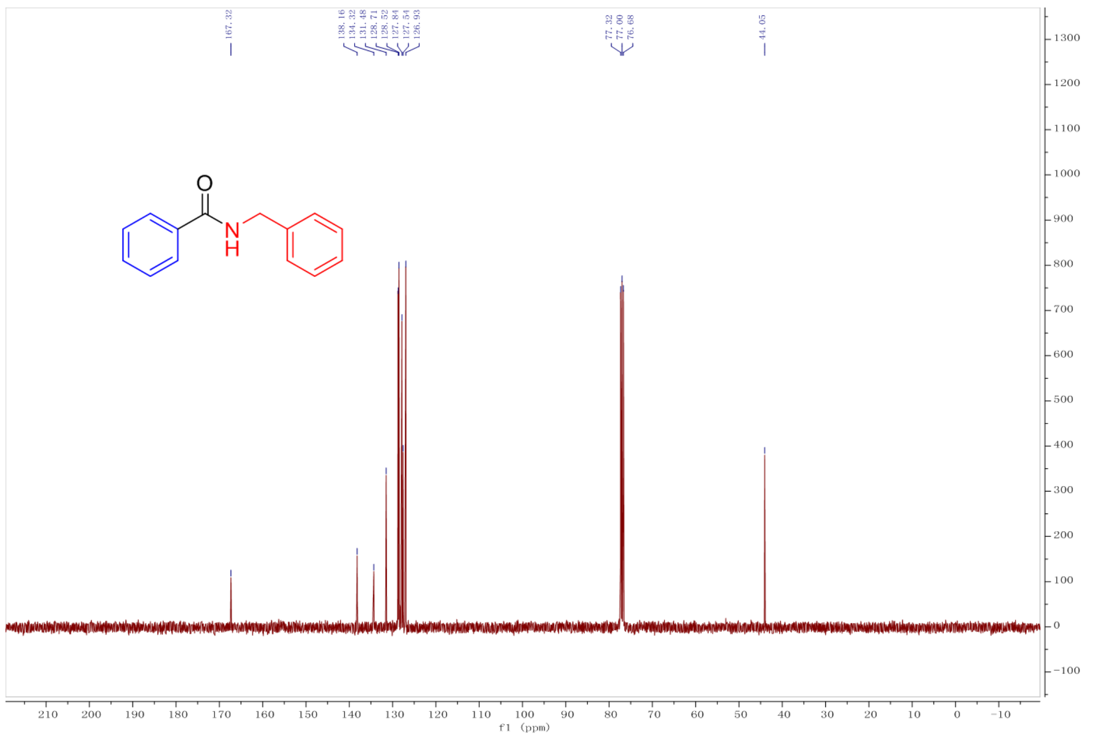

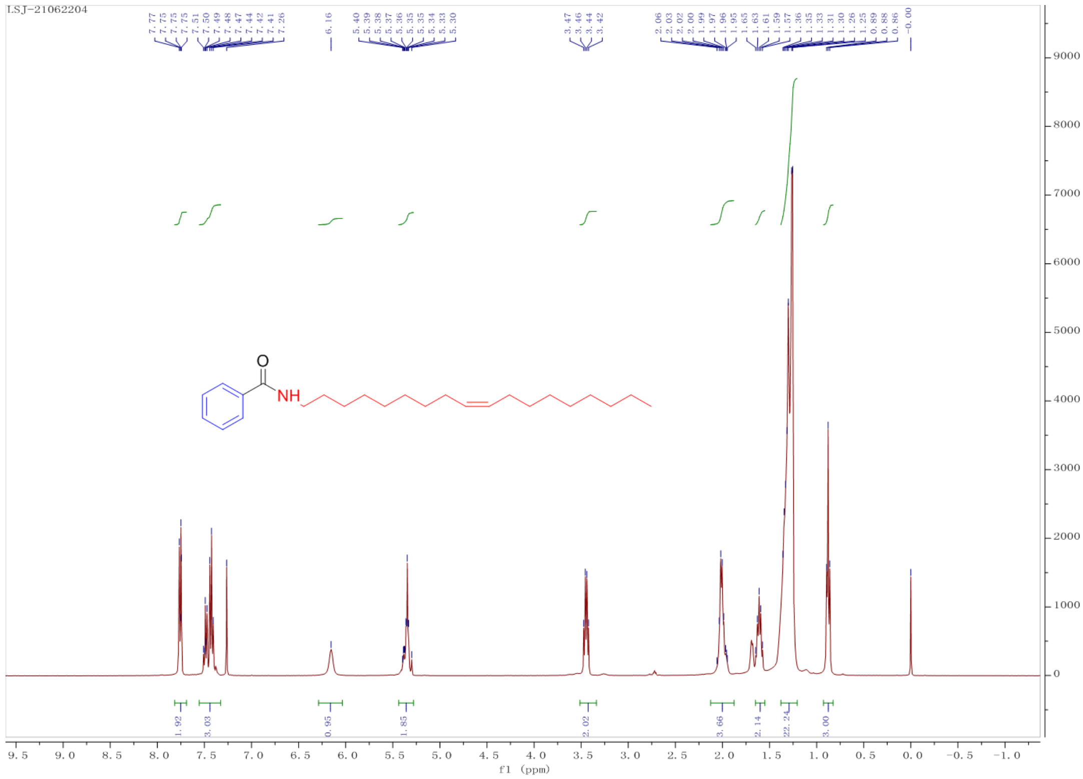

LSJ-21062204
f1 (ppm)

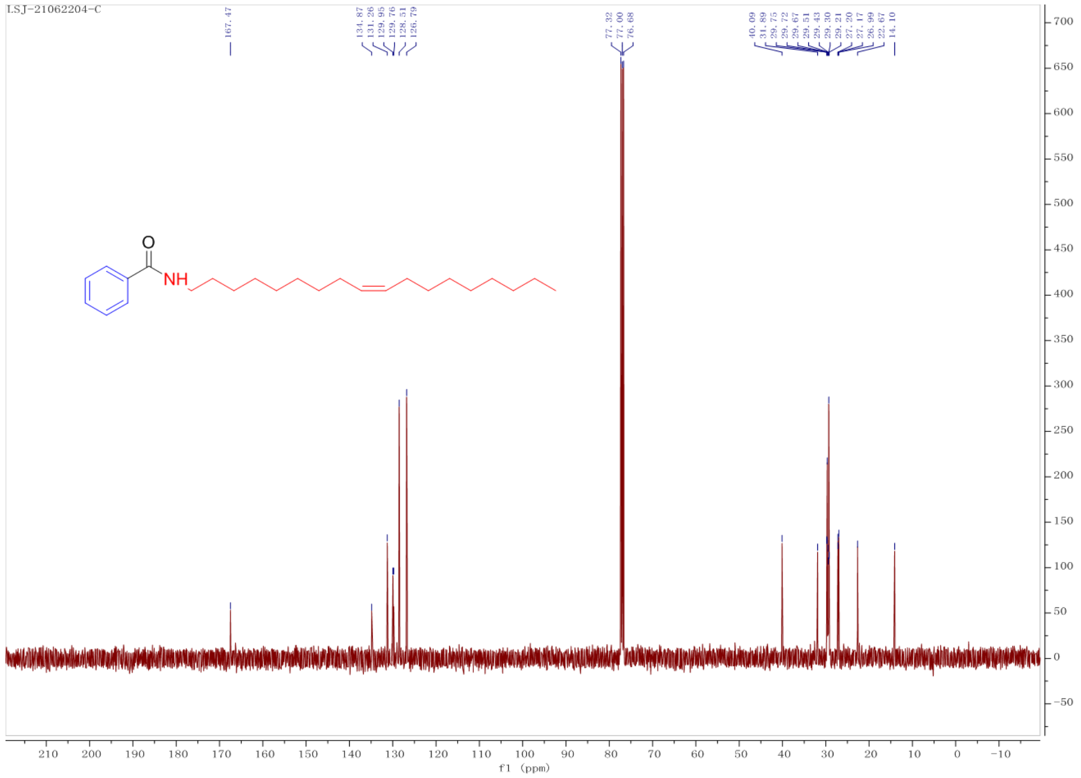

LSJ-21062204-C
f1 (ppm)

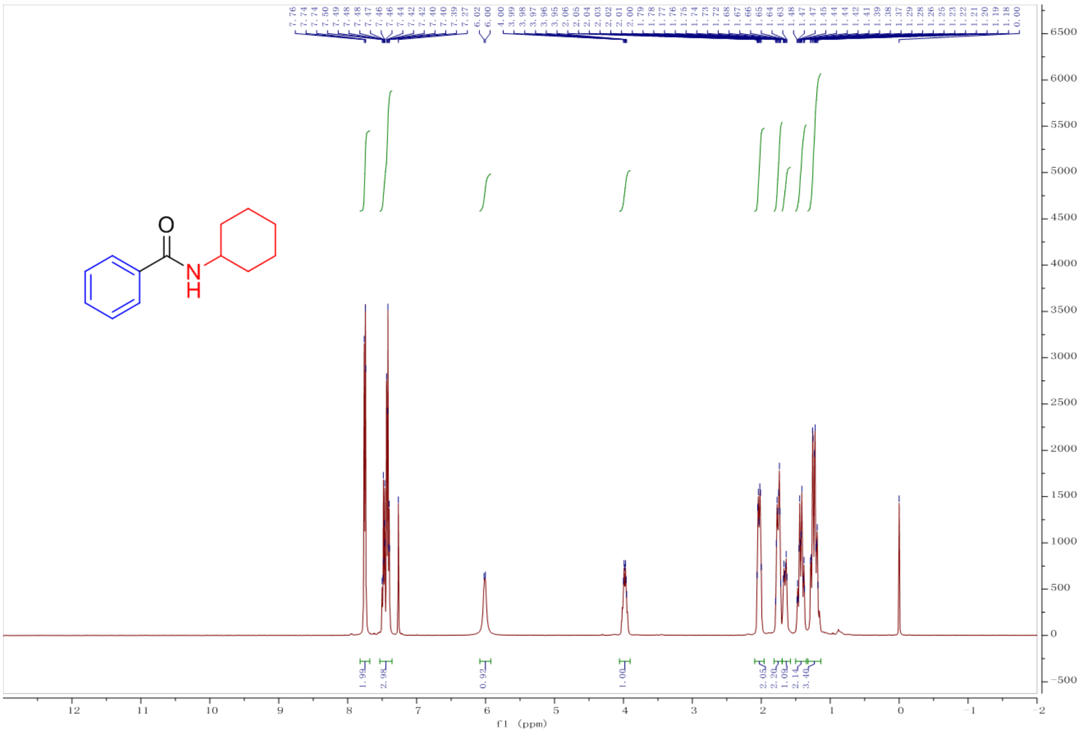

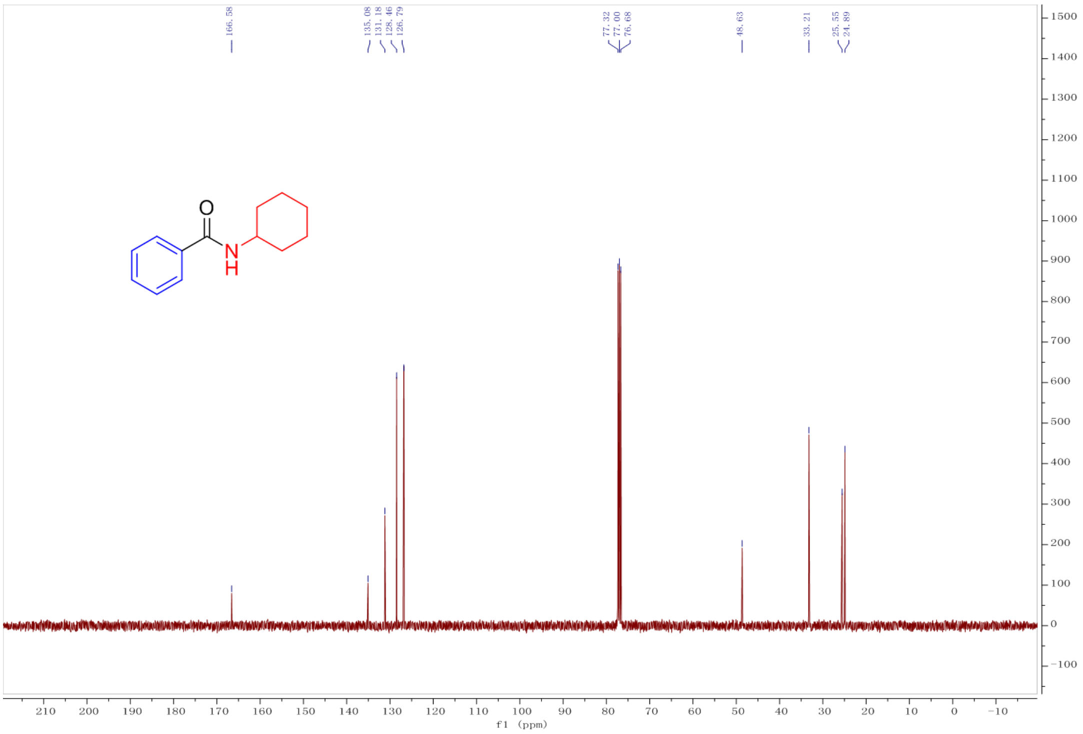

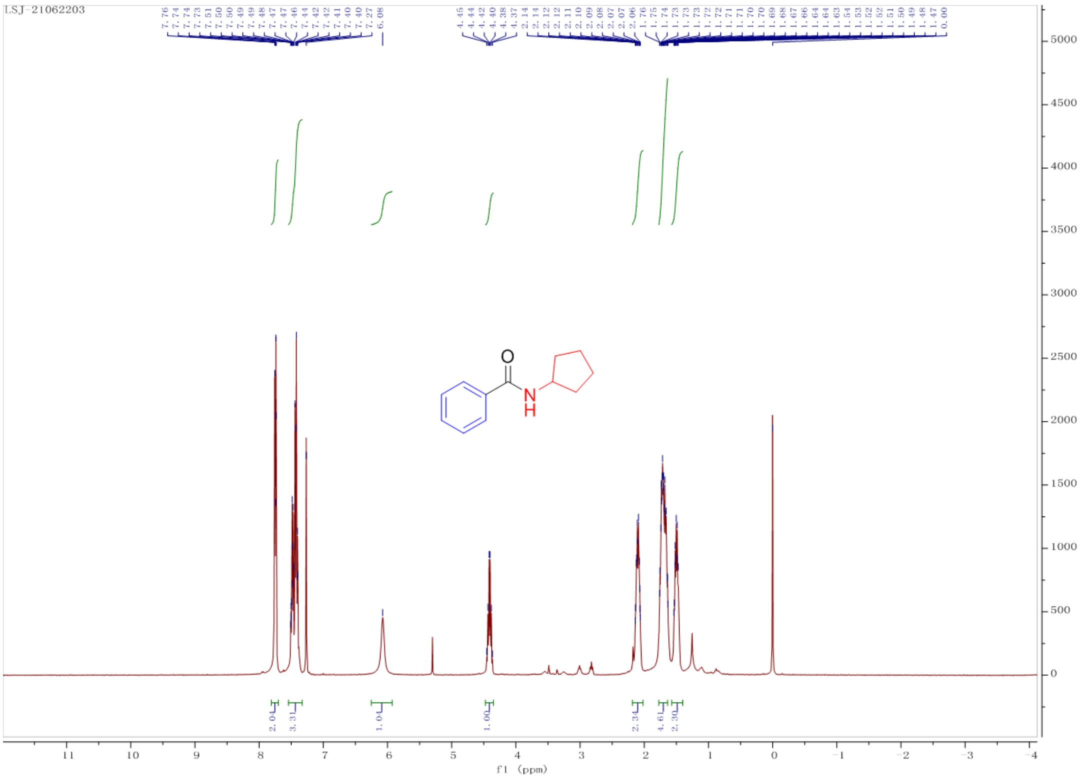
LSJ-21062203
f1 (ppm)

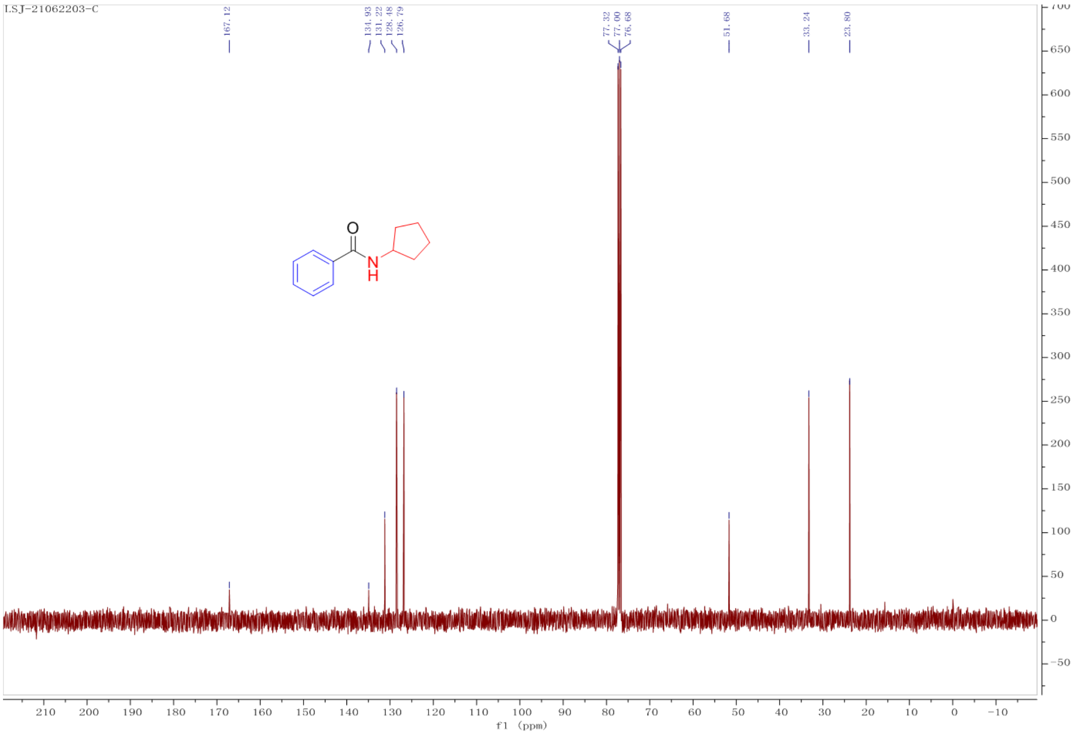
LSJ-21062203-C
167.12
134.93
131.22
128.48
126.79
77.32
77.00
76.68
51.68
33.24
23.80
f1 (ppm)

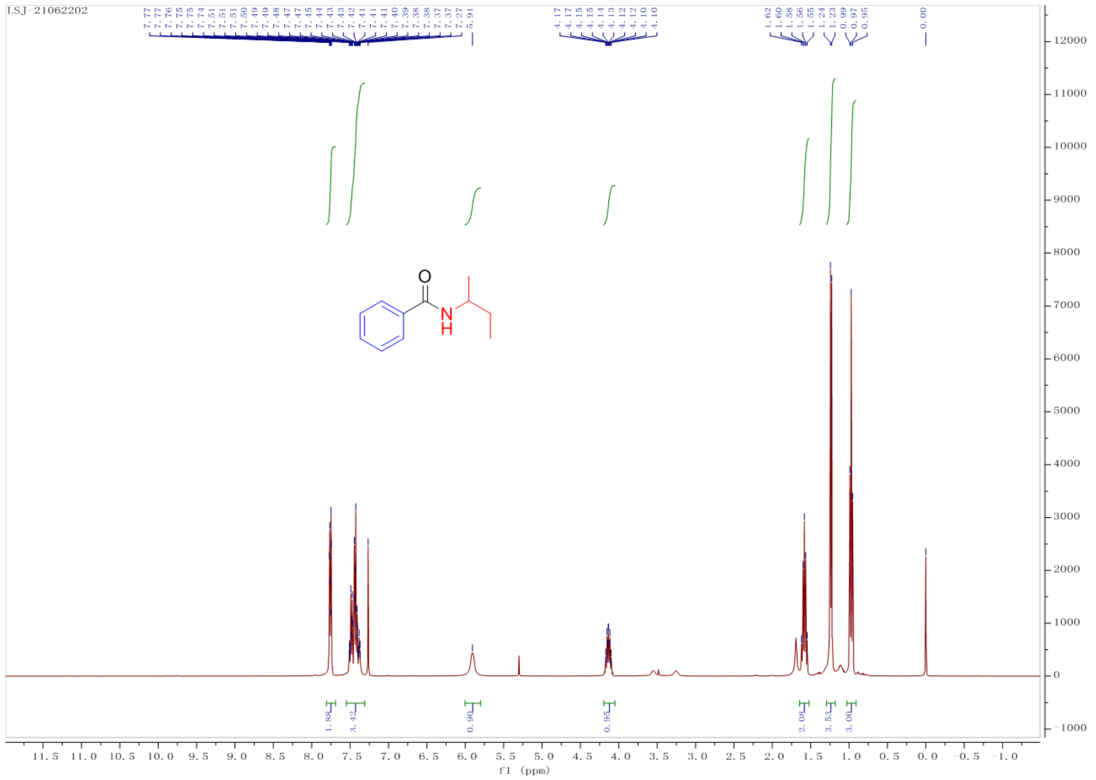
LSJ 21062202
f1 (ppm)

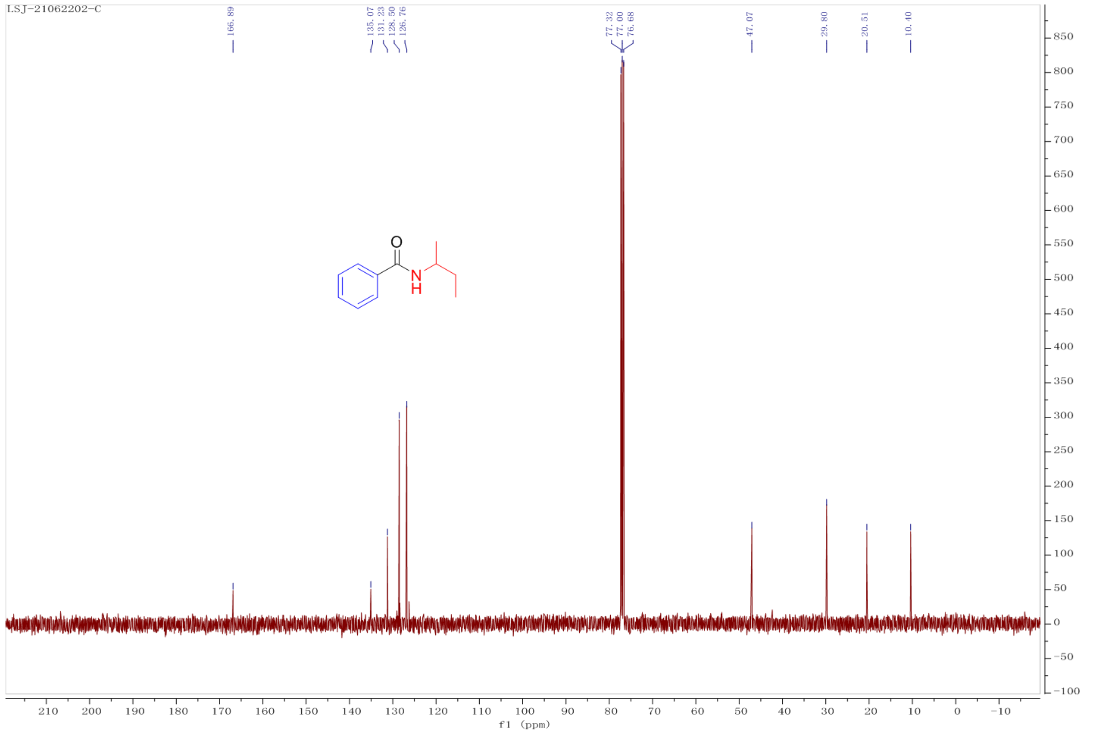
LSJ-21062202-C
166.89
135.07
131.23
128.50
126.76
77.32
77.00
76.68
47.07
29.80
20.51
10.40
f1 (ppm)

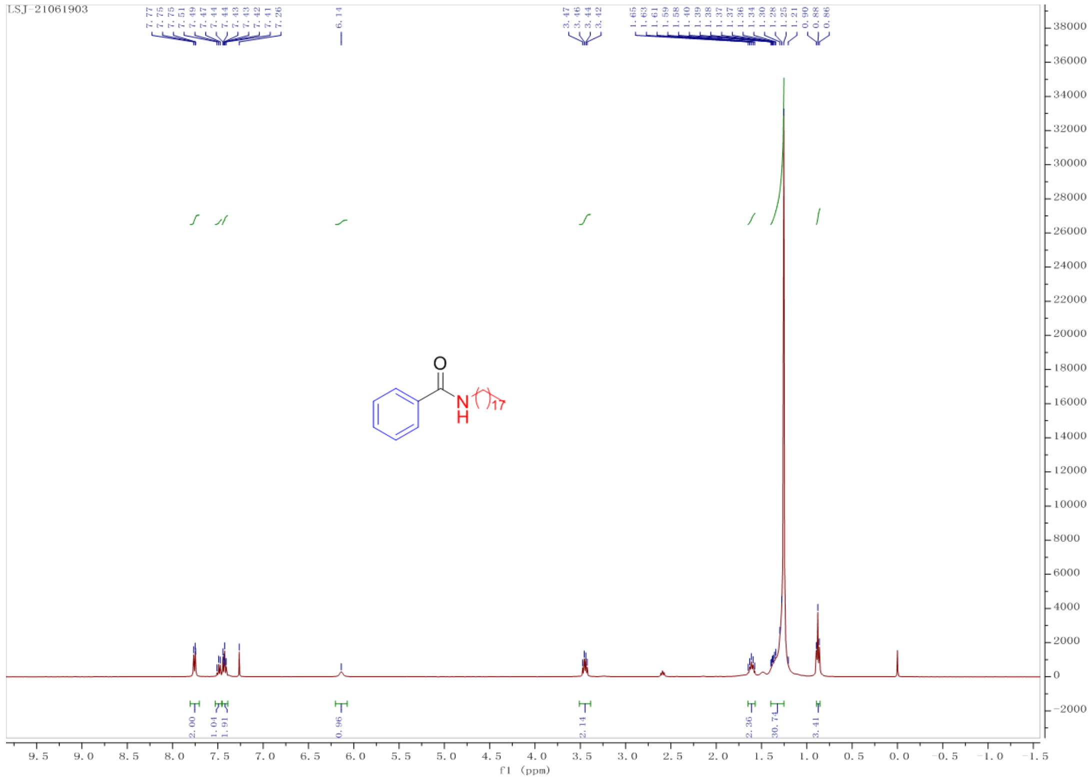

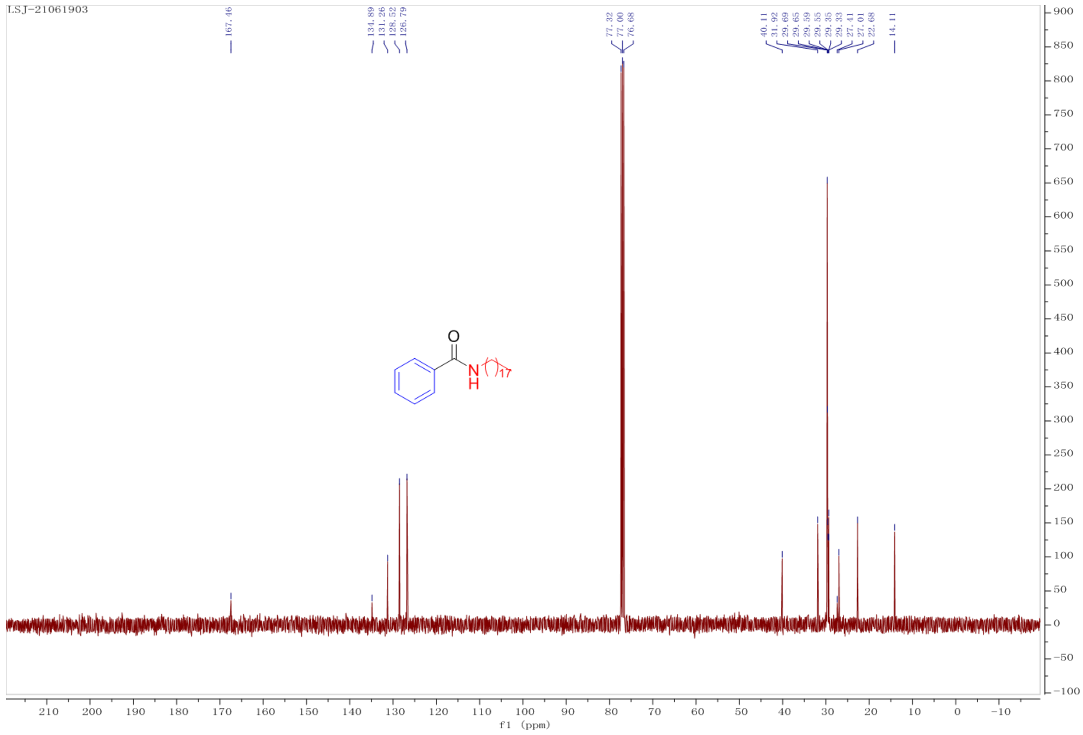

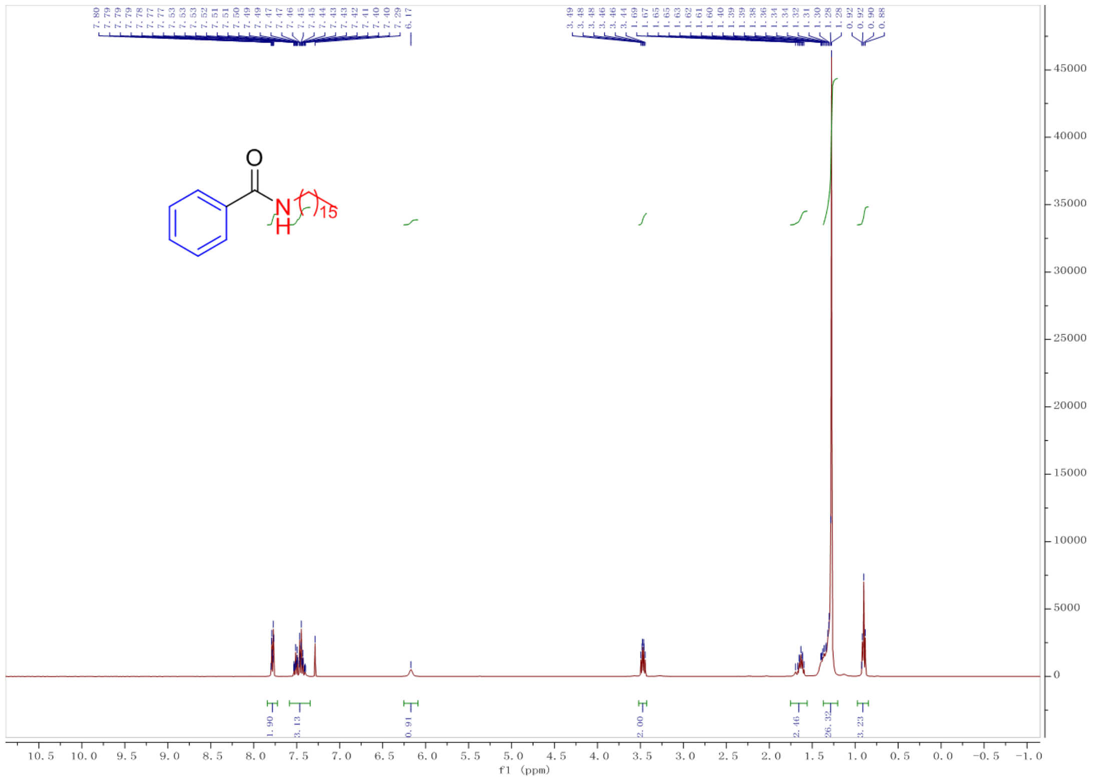

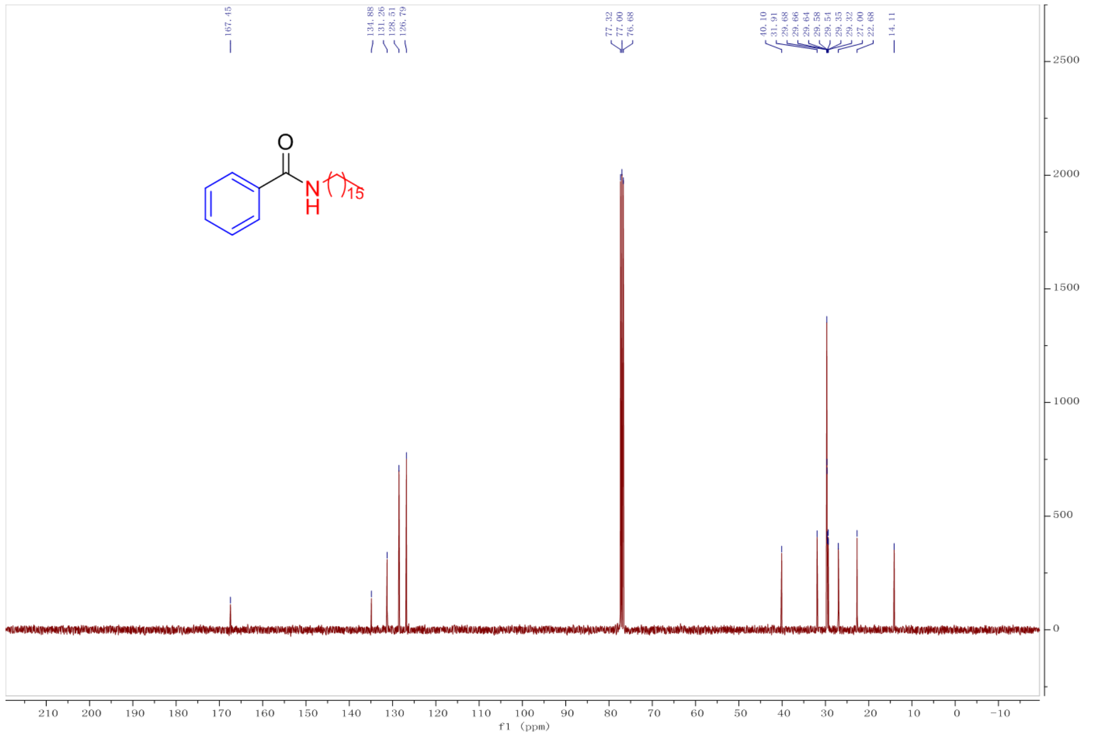

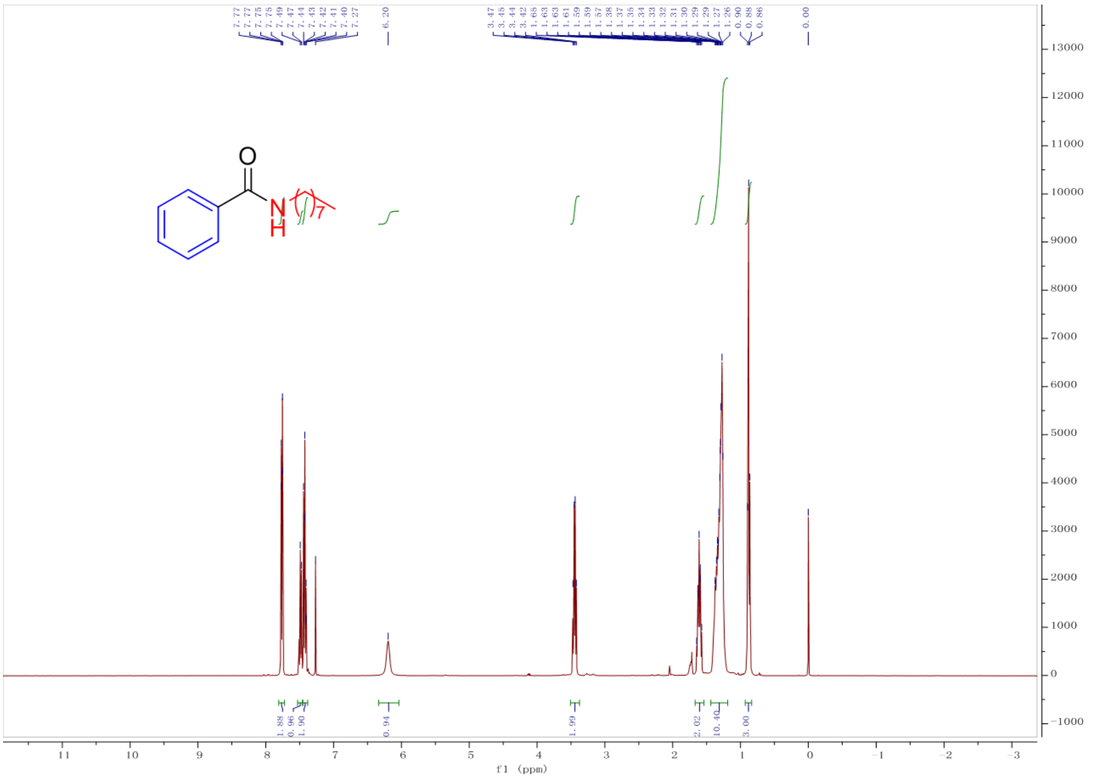

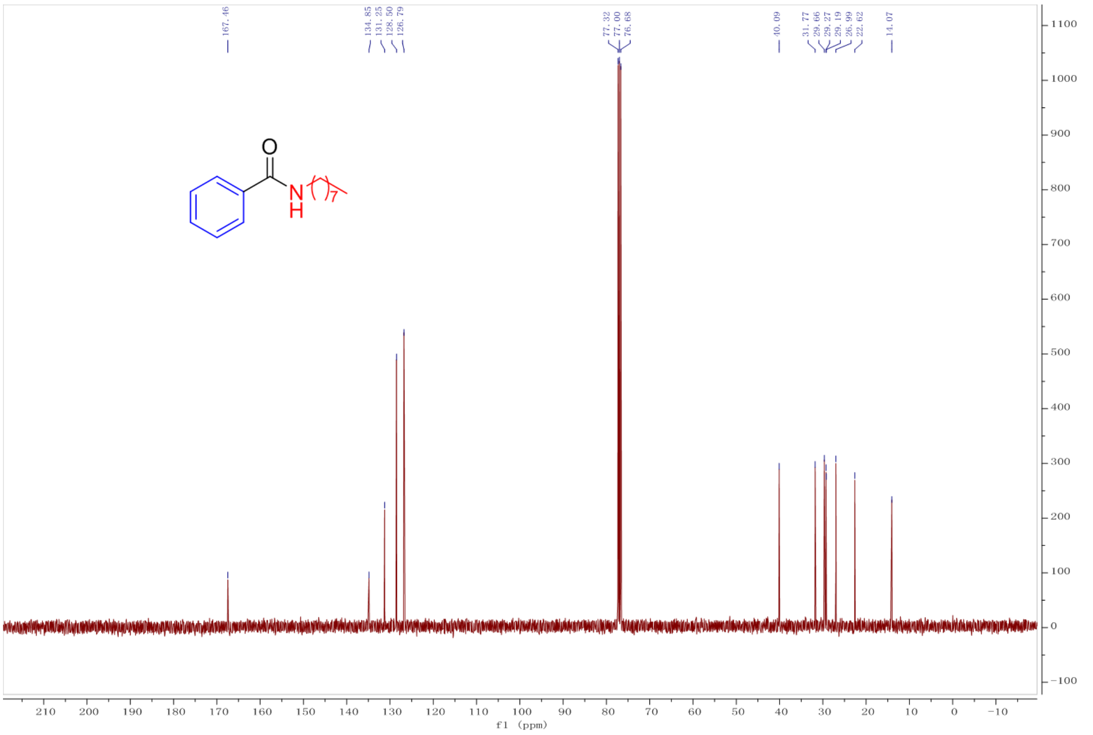

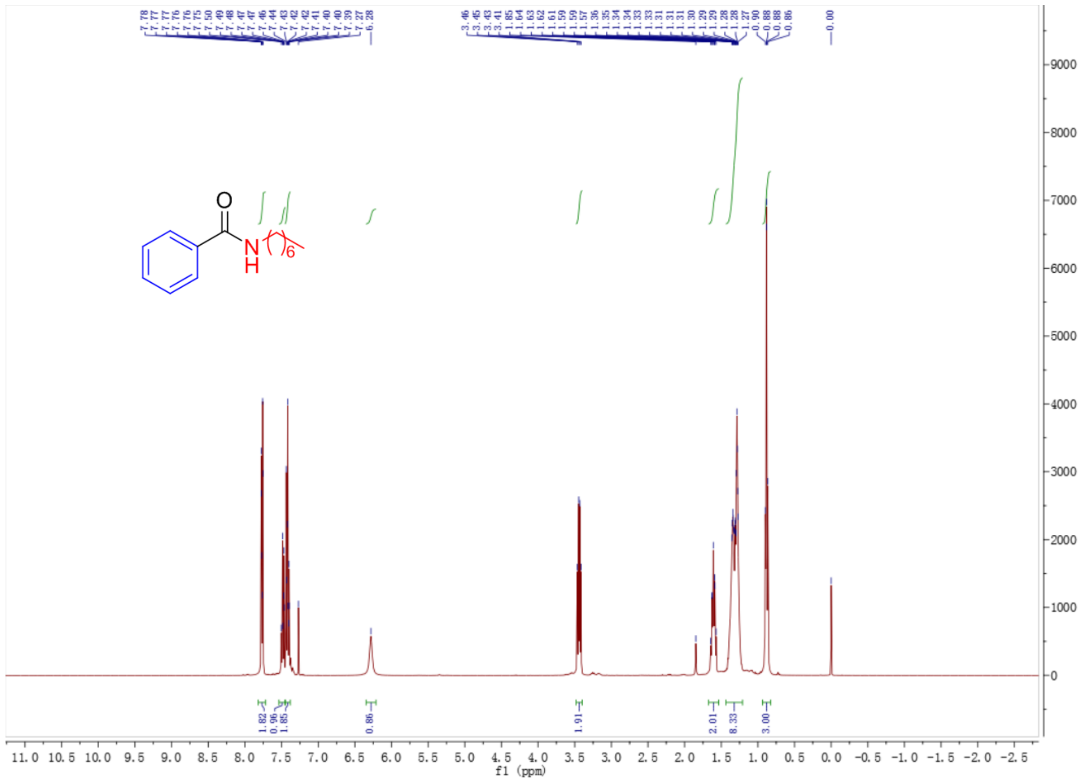

f1 (ppm)

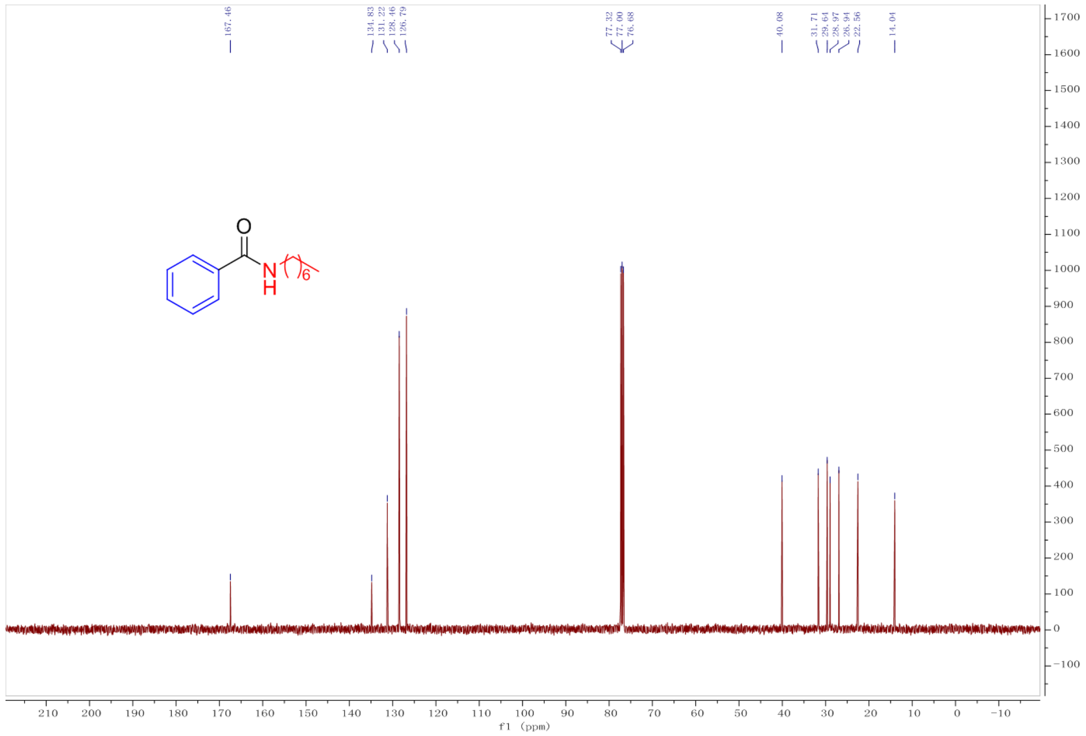

167.46
134.83
131.22
128.46
126.79
77.32
77.00
76.68
40.08
31.71
29.64
28.97
26.94
22.56
14.04
f1 (ppm)

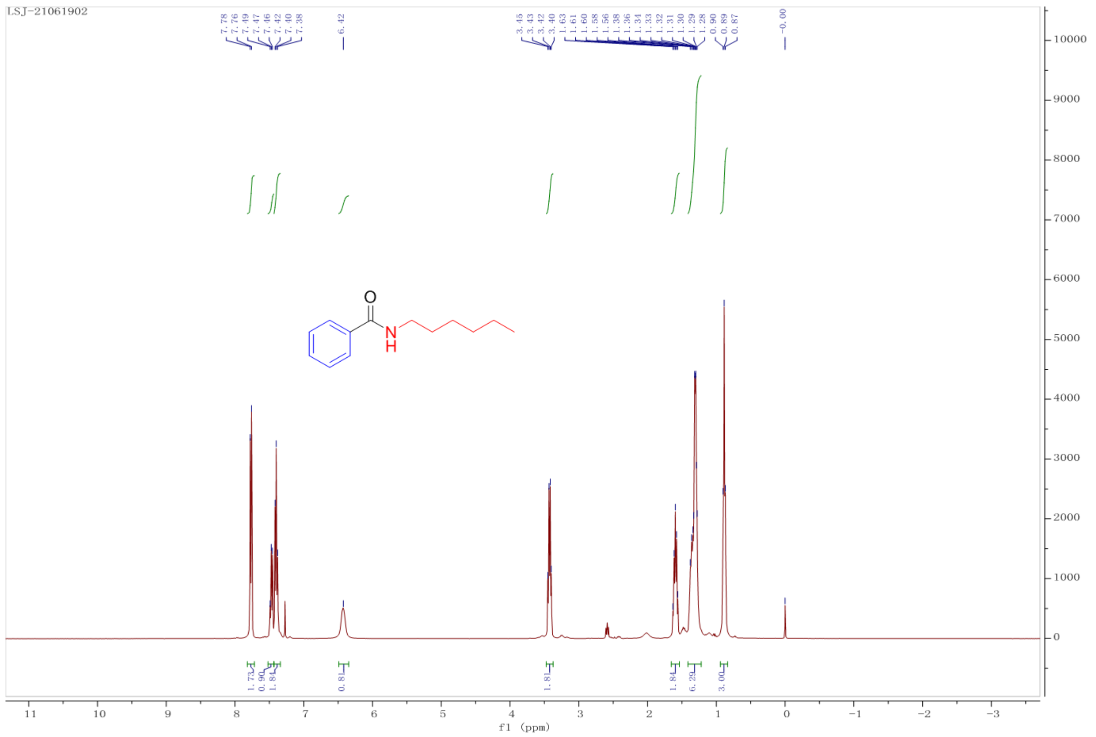
LSJ-21061902
f1 (ppm)

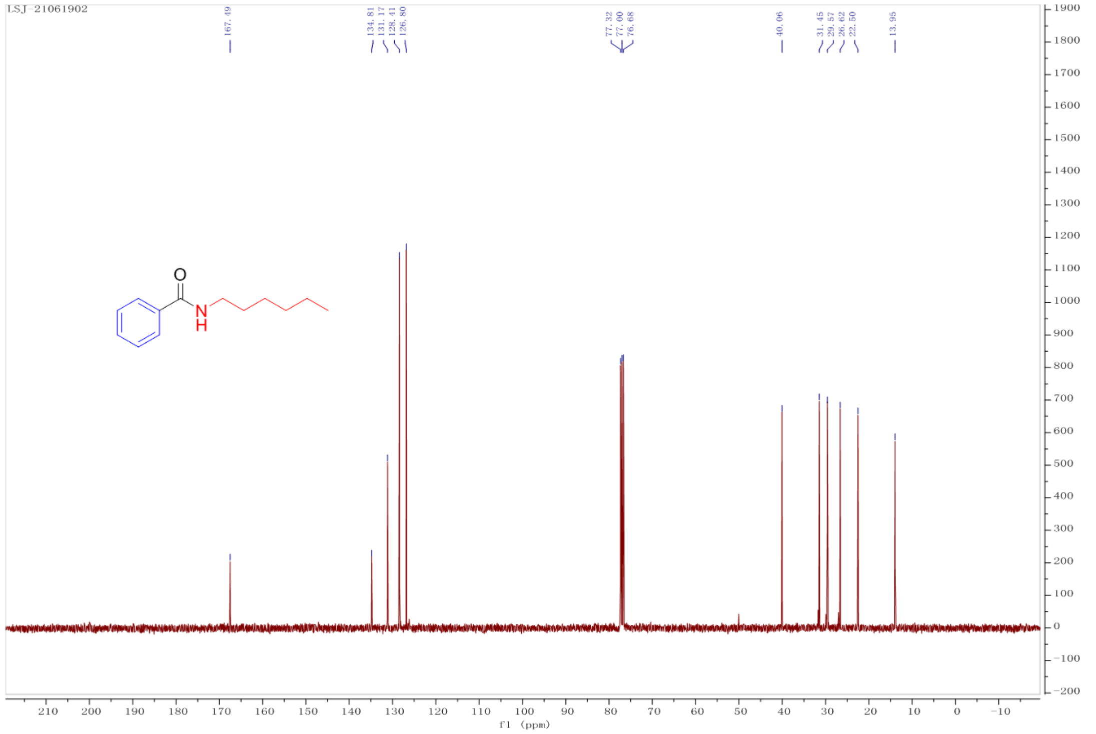
LSJ-21061902
f1 (ppm)

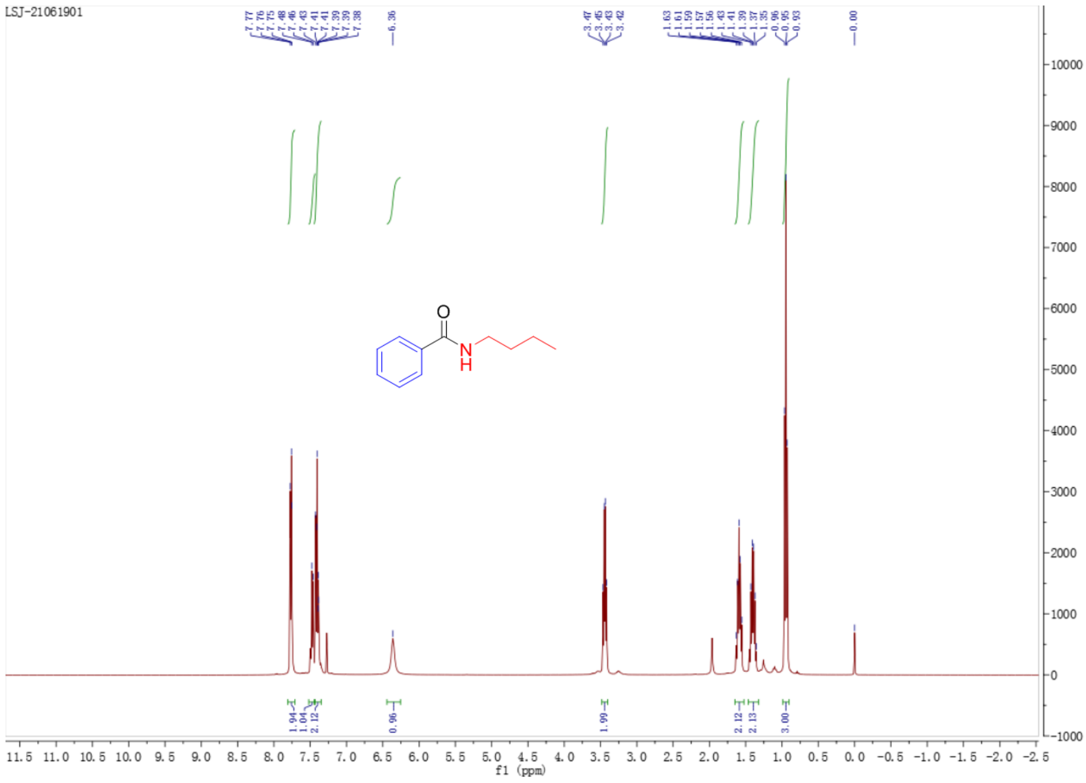
LSJ-21061901
7.77
7.76
7.75
7.48
7.46
7.43
7.41
7.41
7.39
7.39
7.38
6.36
3.47
3.45
3.43
3.42
1.63
1.61
1.59
1.57
1.56
1.43
1.41
1.39
1.37
1.35
0.96
0.95
0.93
0.00
1.94
1.04
2.12
0.96
1.99
2.12
2.13
3.00
O
N
H
f1 (ppm)

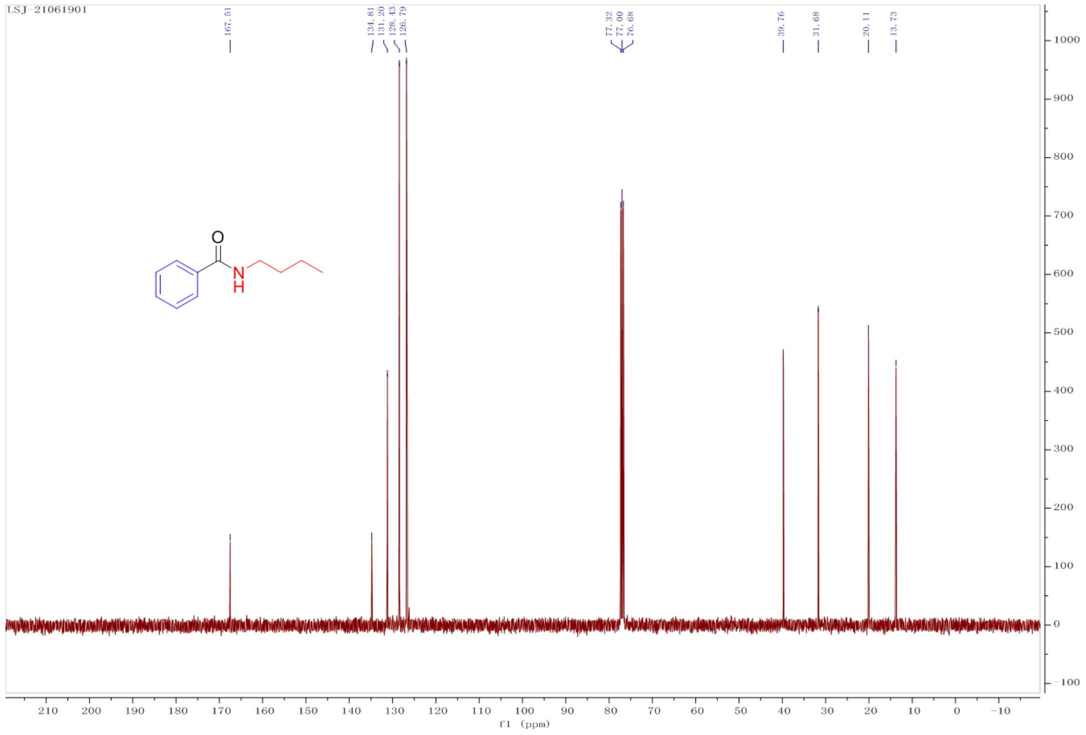
LSJ-21061901
167.51
134.81
131.20
128.43
126.79
77.32
77.00
76.68
39.76
31.68
20.11
13.73
O
N
H
f1 (ppm)

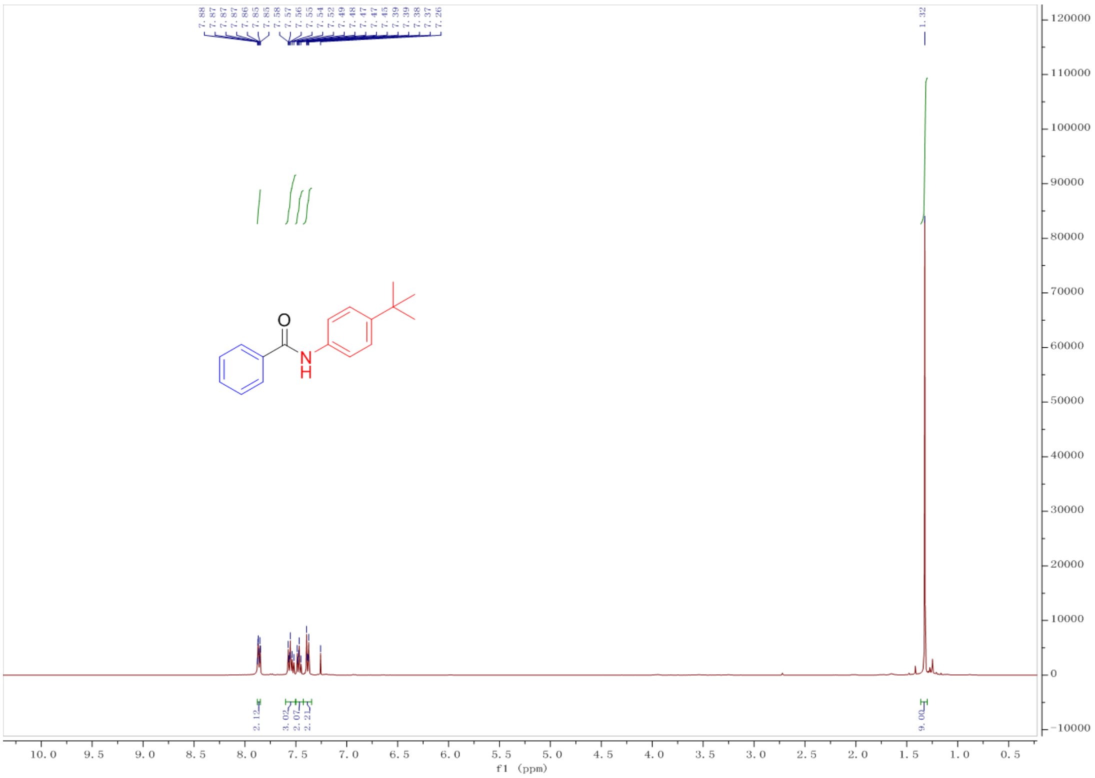

7.88
7.87
7.87
7.87
7.86
7.85
7.85
7.58
7.57
7.56
7.55
7.54
7.52
7.49
7.48
7.47
7.47
7.45
7.39
7.39
7.38
7.37
7.26
1.32
O
N
H
2.12
3.02
2.07
2.21
9.00
f1 (ppm)

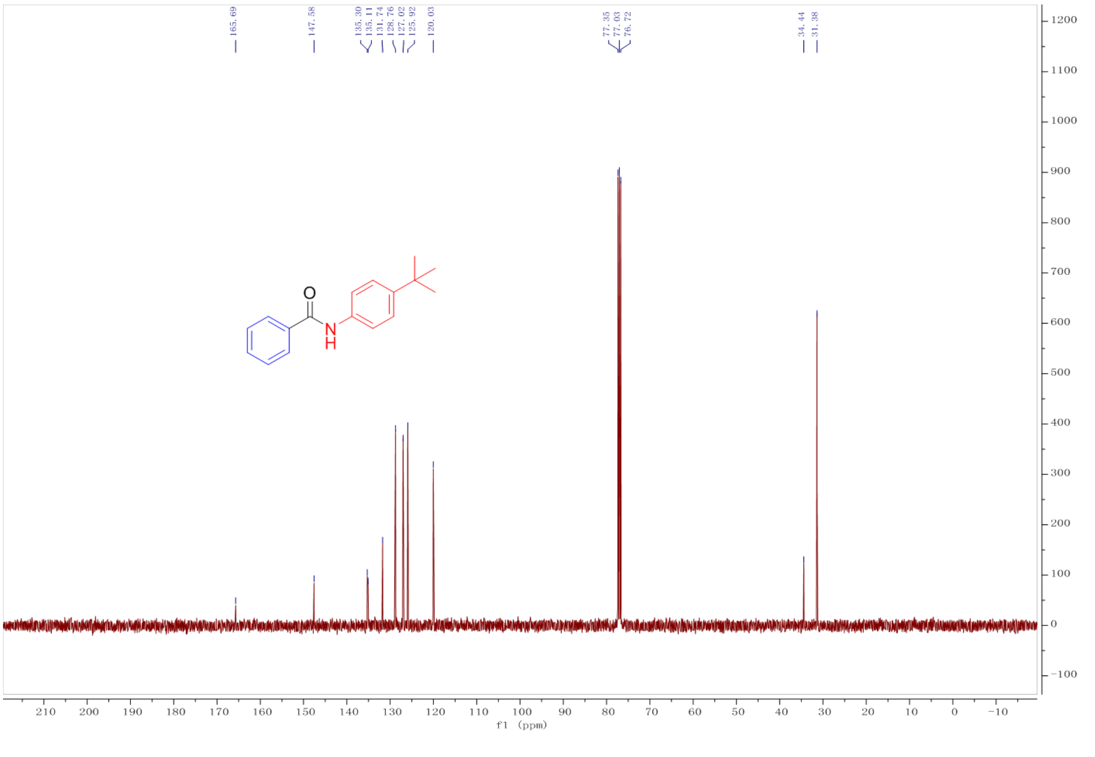

165.69
147.58
135.30
135.11
131.74
128.76
127.02
125.92
120.03
77.35
77.03
76.72
34.44
31.38
O
N
H
f1 (ppm)

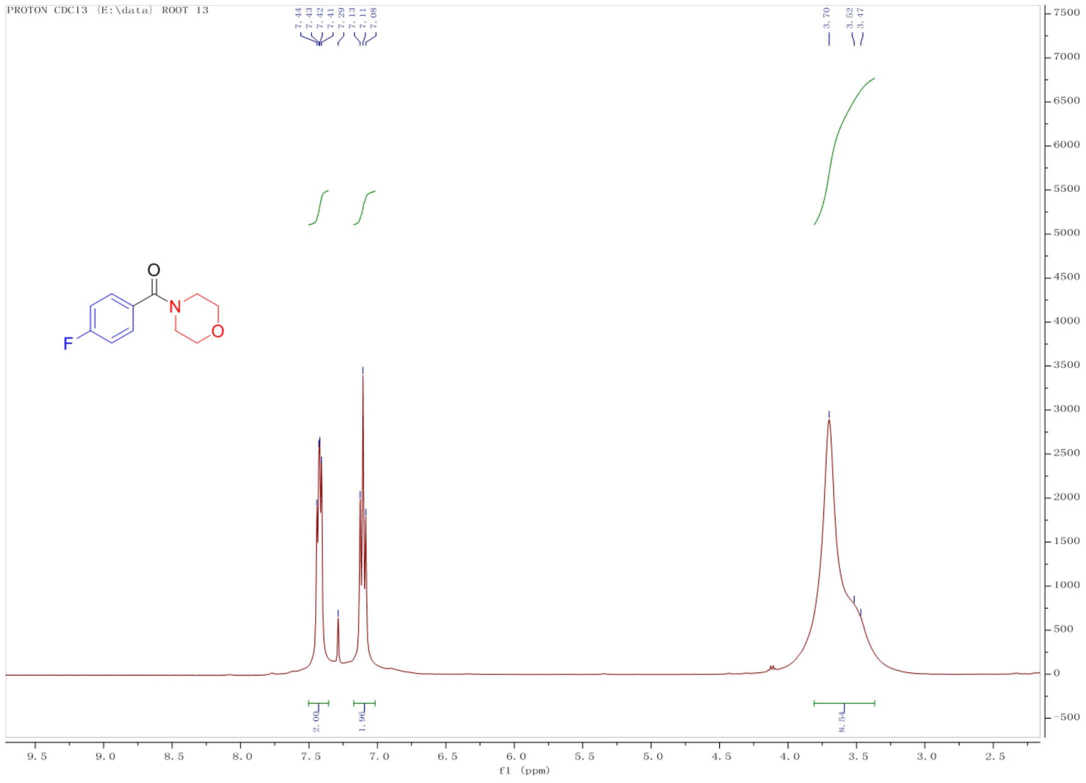
PROTON CDCl3 {E:\data} ROOT 13
7.44
7.43
7.42
7.41
7.29
7.13
7.11
7.08
3.70
3.52
3.47
F
O
N
O
2.00
1.96
8.54
f1 (ppm)

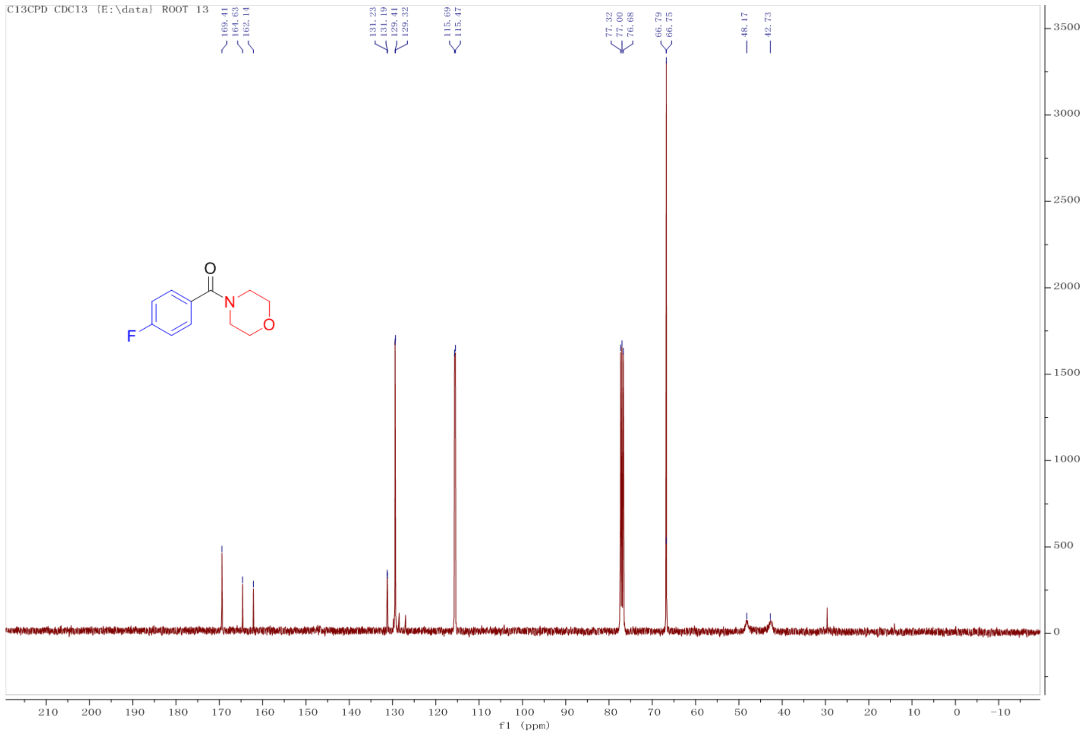
C13CPD CDCl3 {E:\data} ROOT 13
169.41
164.63
162.14
131.23
131.19
129.41
129.32
115.69
115.47
77.32
77.00
76.68
66.79
66.75
48.17
42.73
F
O
N
O
f1 (ppm)

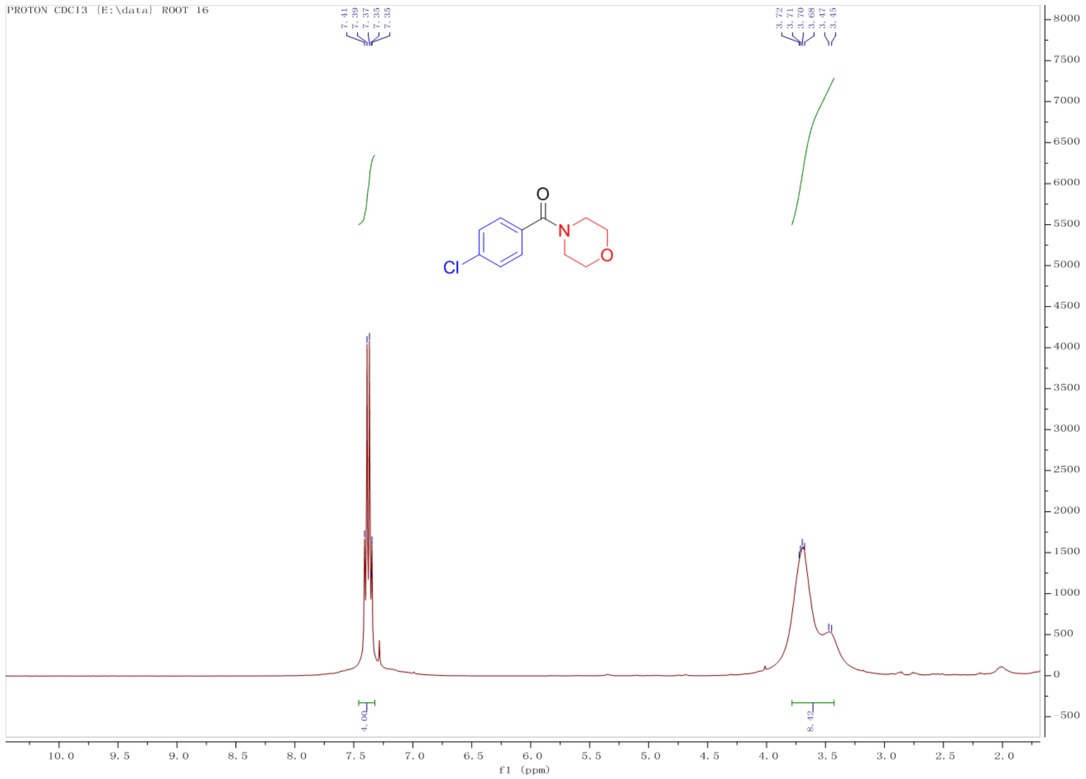
PROTON CDCl3 {E:\data} ROOT 16
Cl
O
N
O
f1 (ppm)

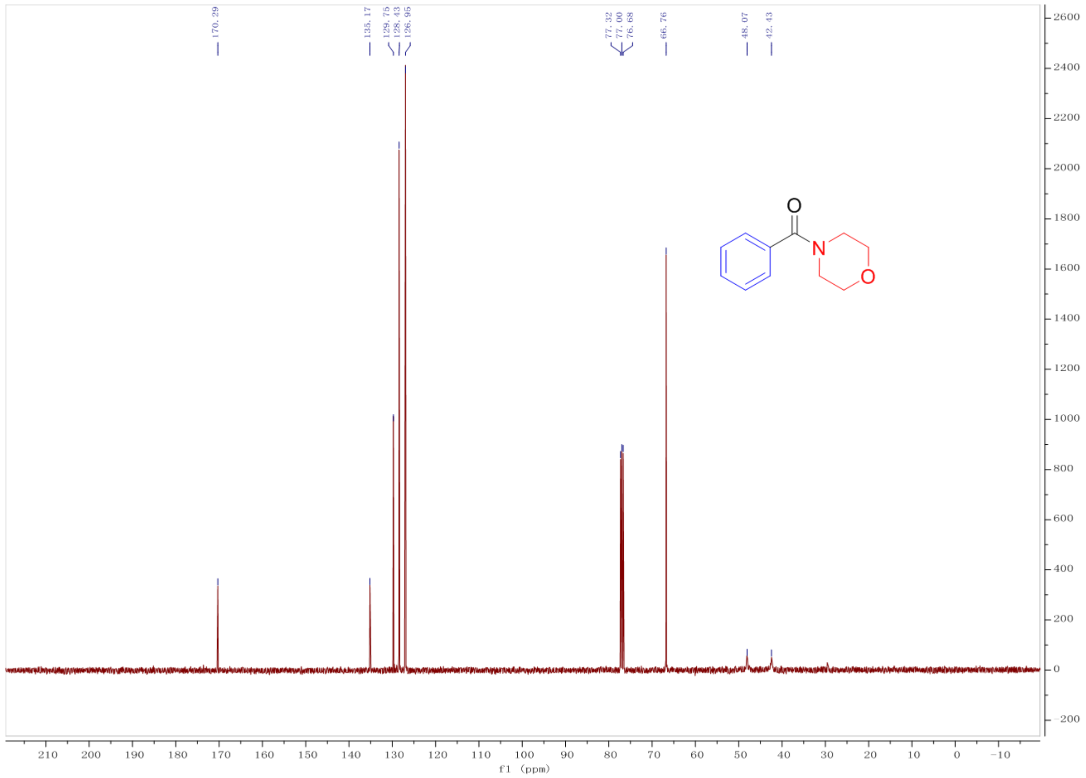
O
N
O
f1 (ppm)

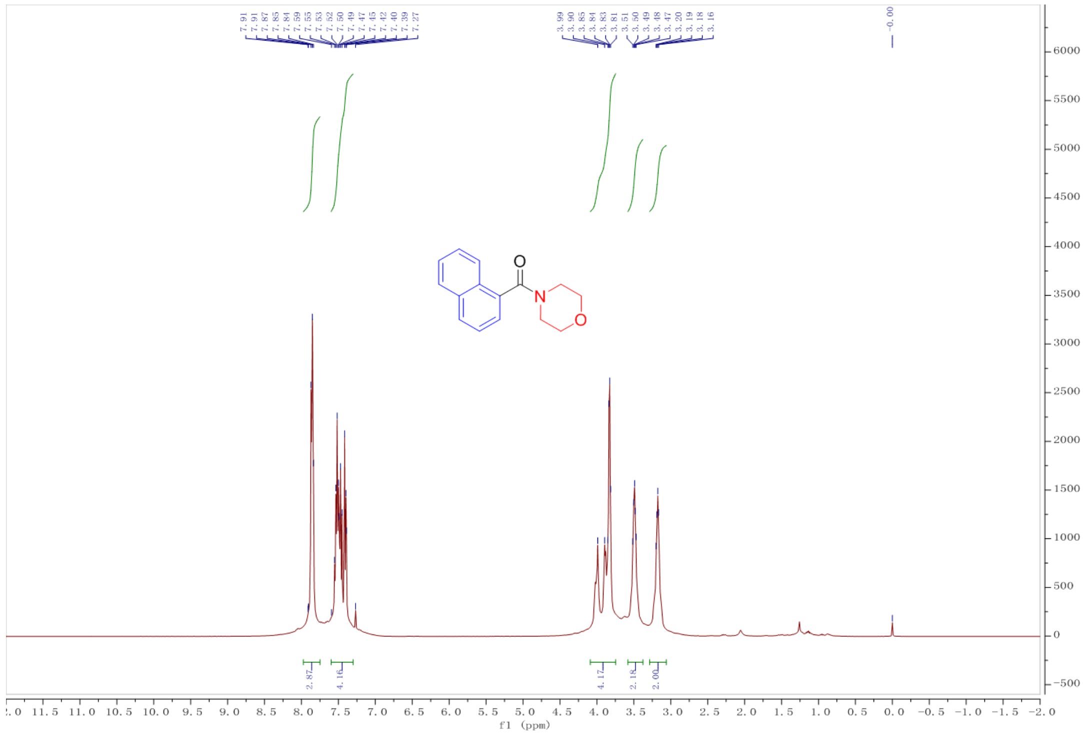

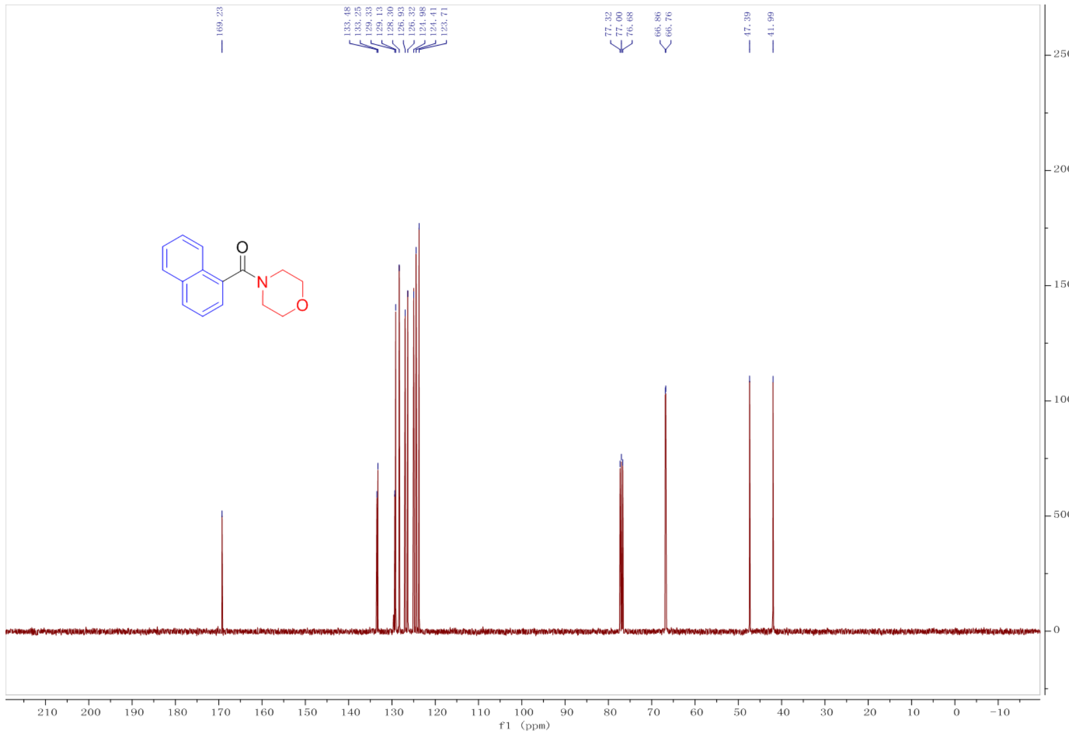

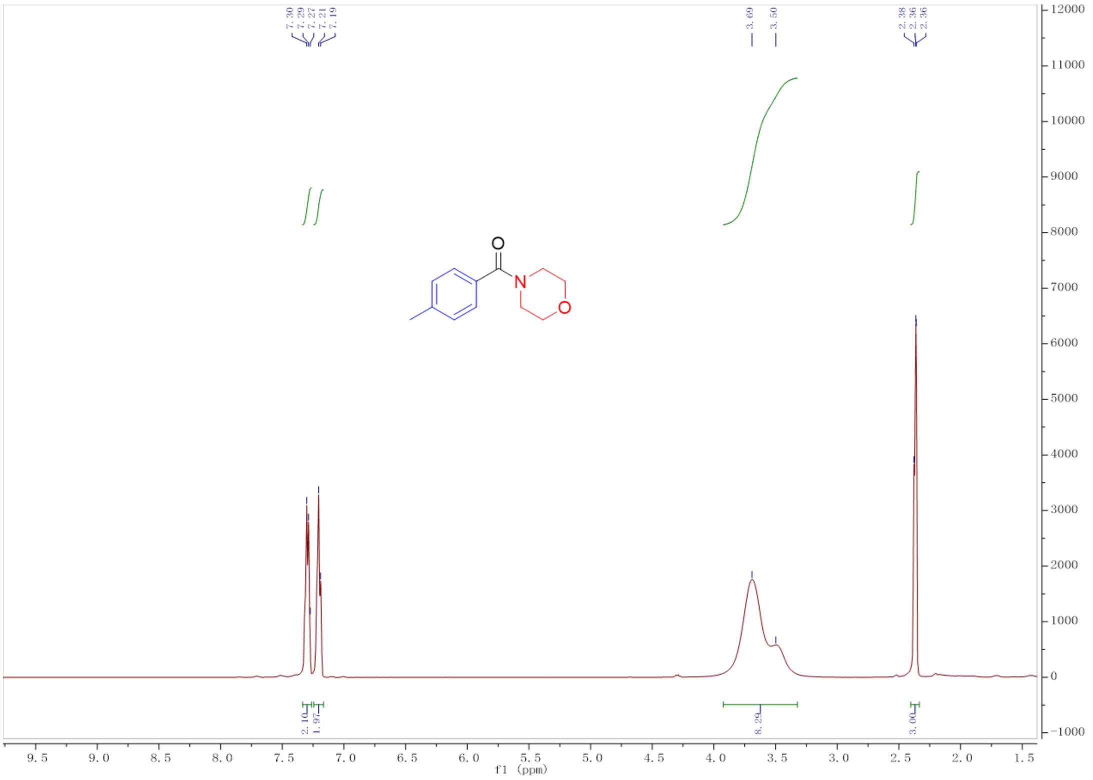
7.30
7.29
7.27
7.21
7.19
3.69
3.50
2.38
2.36
2.36
2.10
1.97
8.29
3.00
f1 (ppm)
O
N
O

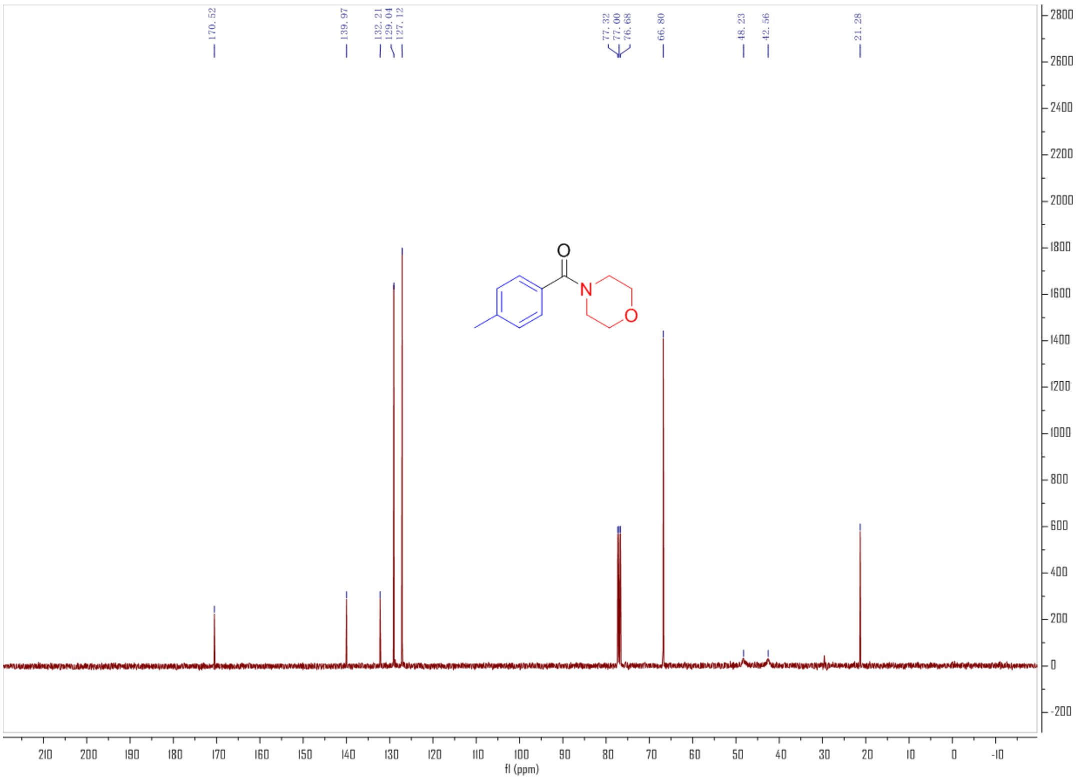
170.52
139.97
132.21
129.04
127.12
77.32
77.00
76.68
66.80
48.23
42.56
21.28
fl (ppm)
O
N
O

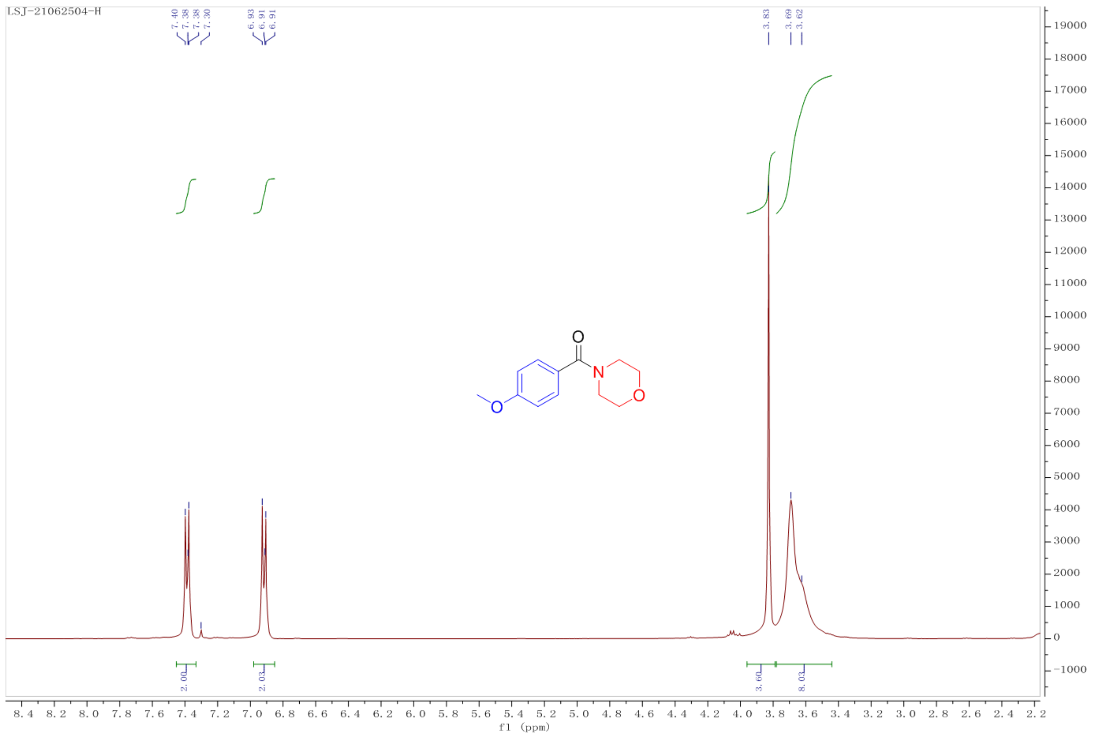
LSJ-21062504-H
7.40
7.38
7.38
7.30
6.93
6.91
6.91
3.83
3.69
3.62
2.00
2.03
3.60
8.03
f1 (ppm)

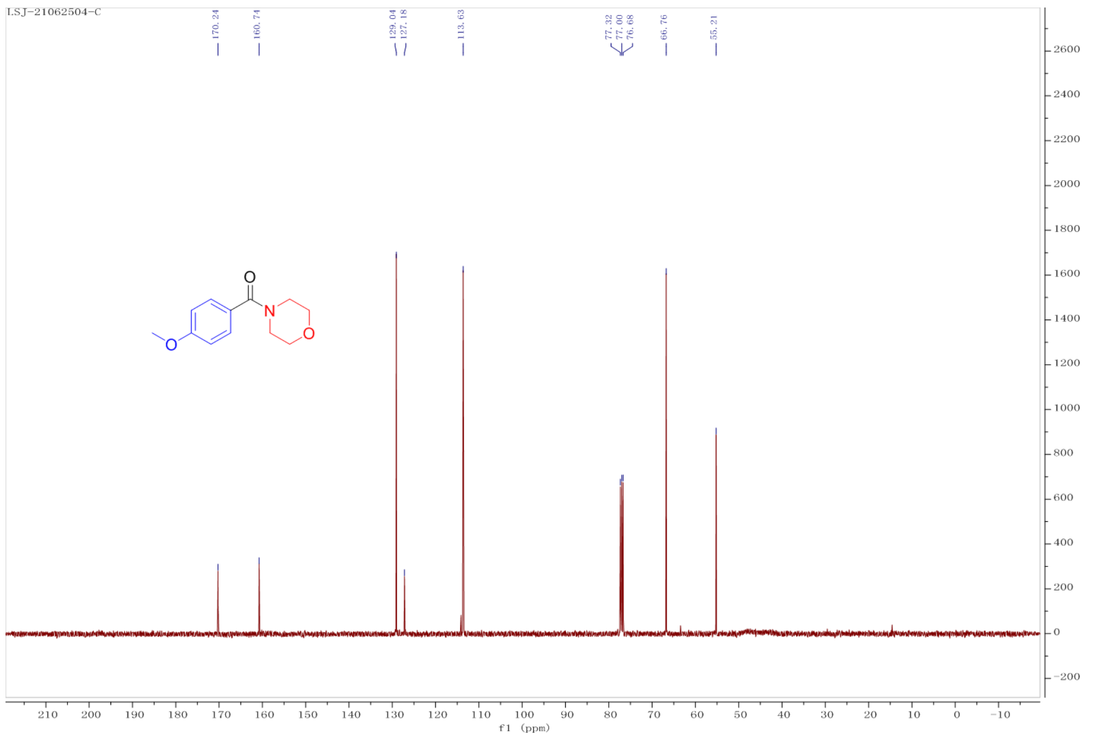
LSJ-21062504-C
170.24
160.74
129.04
127.18
113.63
77.32
77.00
76.68
66.76
55.21
f1 (ppm)

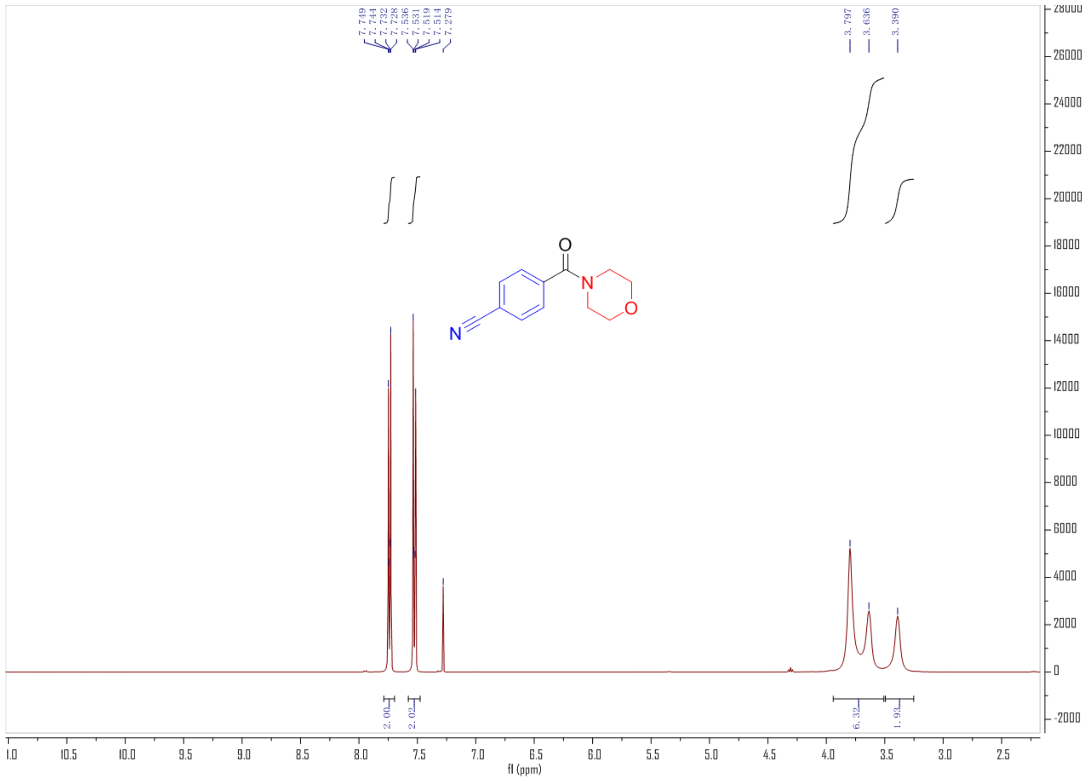

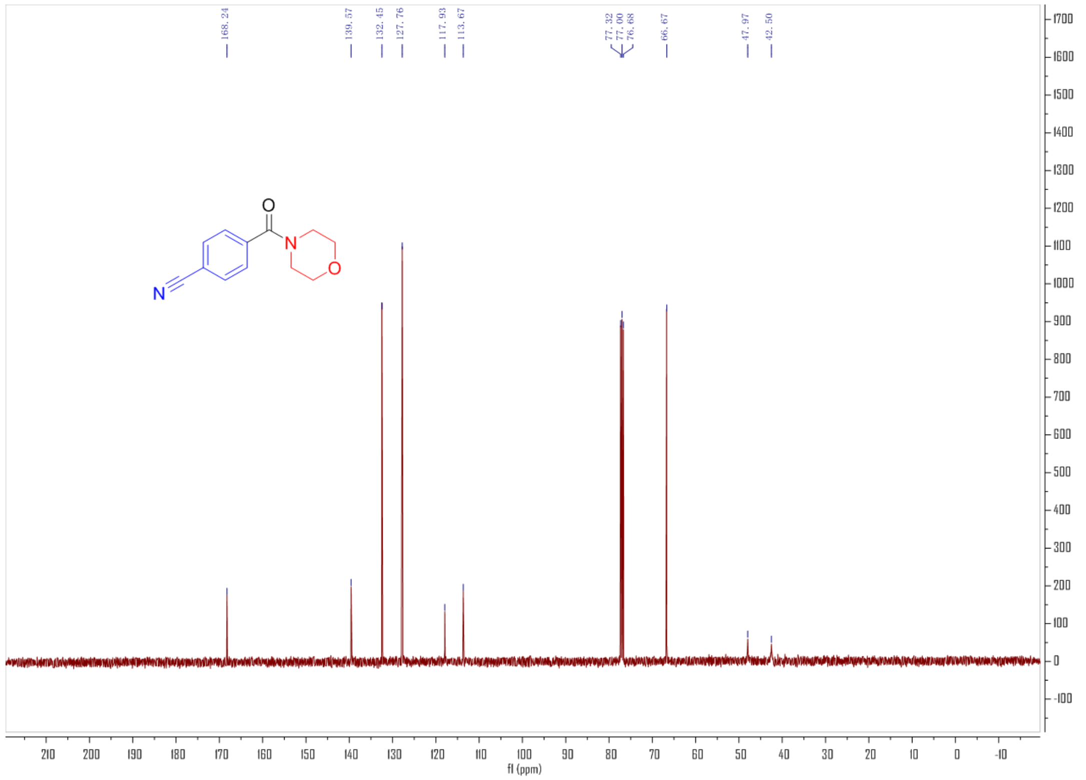

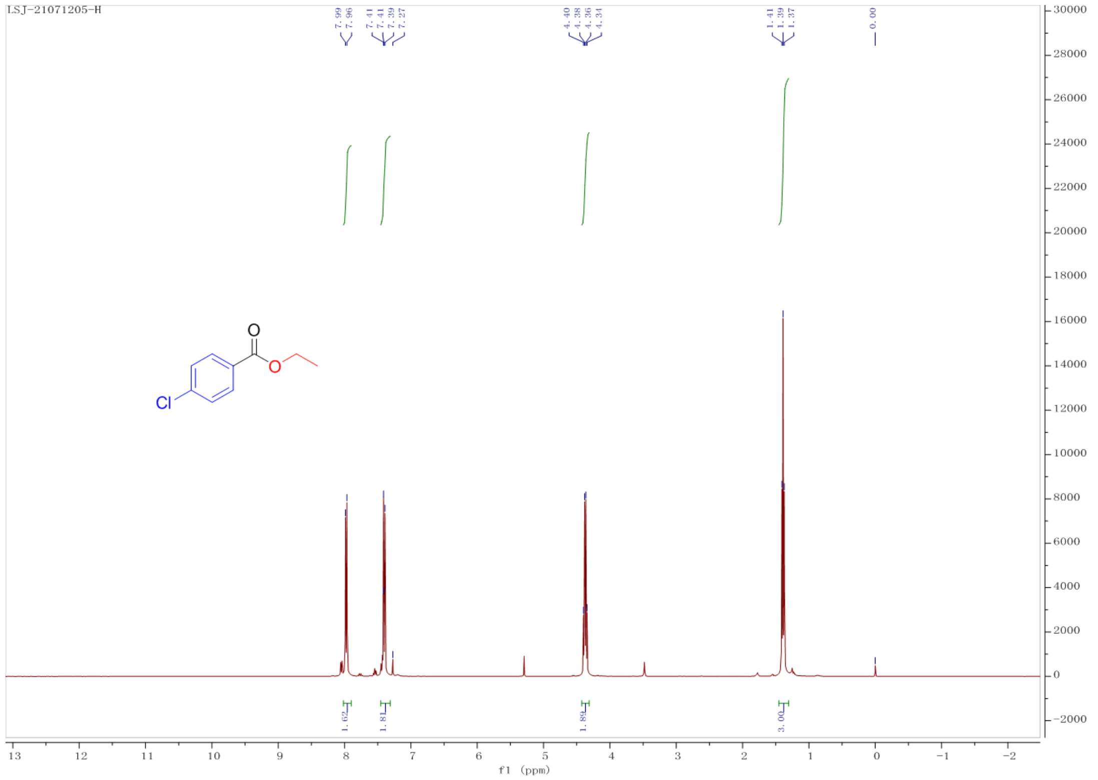
LSJ-21071205-H
7.99
7.96
7.41
7.41
7.39
7.27
4.40
4.38
4.36
4.34
1.41
1.39
1.37
0.00
Cl
O
1.62
1.81
1.89
3.00
f1 (ppm)

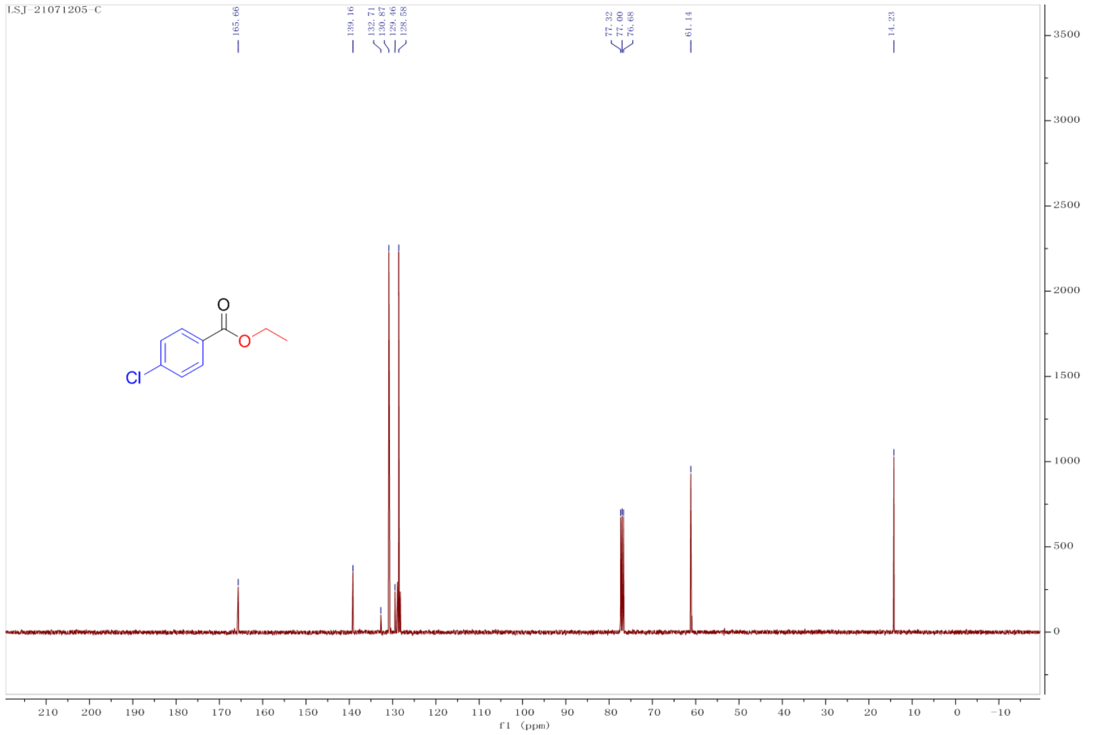
LSJ-21071205-C
165.66
139.16
132.71
130.87
129.46
128.58
77.32
77.00
76.68
61.14
14.23
Cl
O
f1 (ppm)

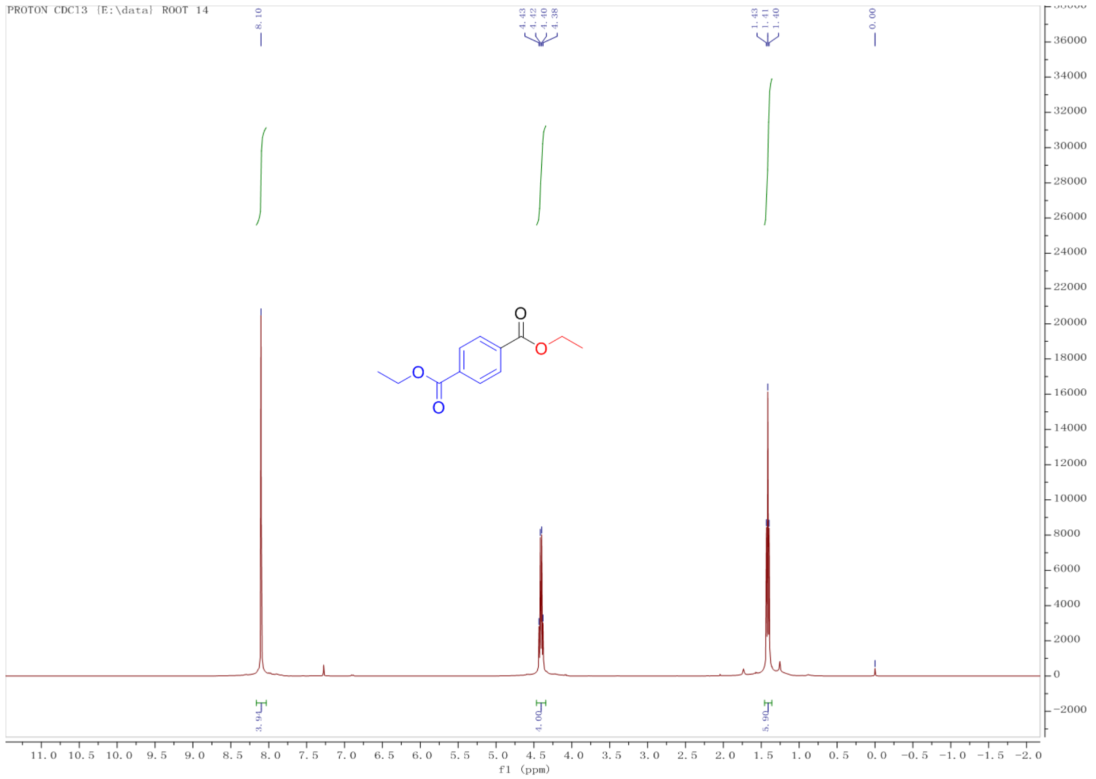
PROTON CDCl3 {E:\data} ROOT 14
8.10
4.43
4.42
4.40
4.38
1.43
1.41
1.40
0.00
3.94
4.00
5.90
O
O
O
O
f1 (ppm)

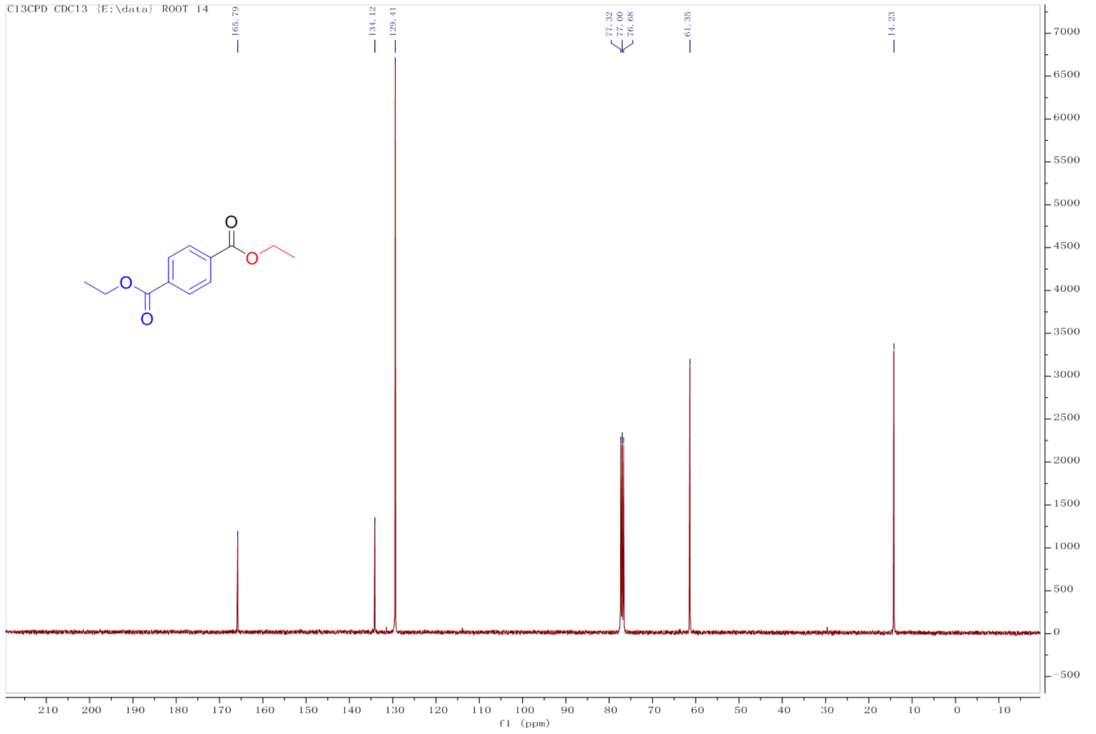
C13CPD CDCl3 {E:\data} ROOT 14
165.79
134.12
129.41
77.32
77.00
76.68
61.35
14.23
O
O
O
O
f1 (ppm)

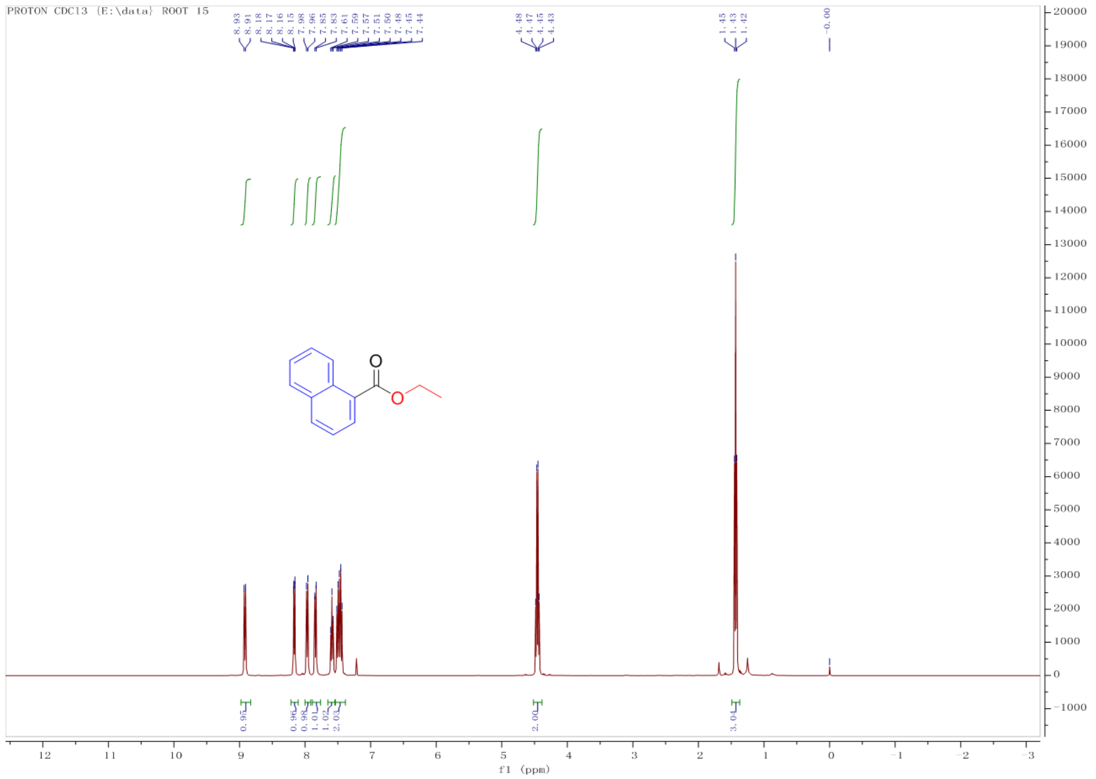
PROTON CDCl3 {E:\data} ROOT 15
8.93
8.91
8.18
8.17
8.16
8.15
7.98
7.96
7.85
7.83
7.61
7.59
7.57
7.51
7.50
7.48
7.45
7.44
4.48
4.47
4.45
4.43
1.45
1.43
1.42
-0.00
0.95
0.96
0.98
1.01
1.02
2.03
2.00
3.04
O
O
f1 (ppm)

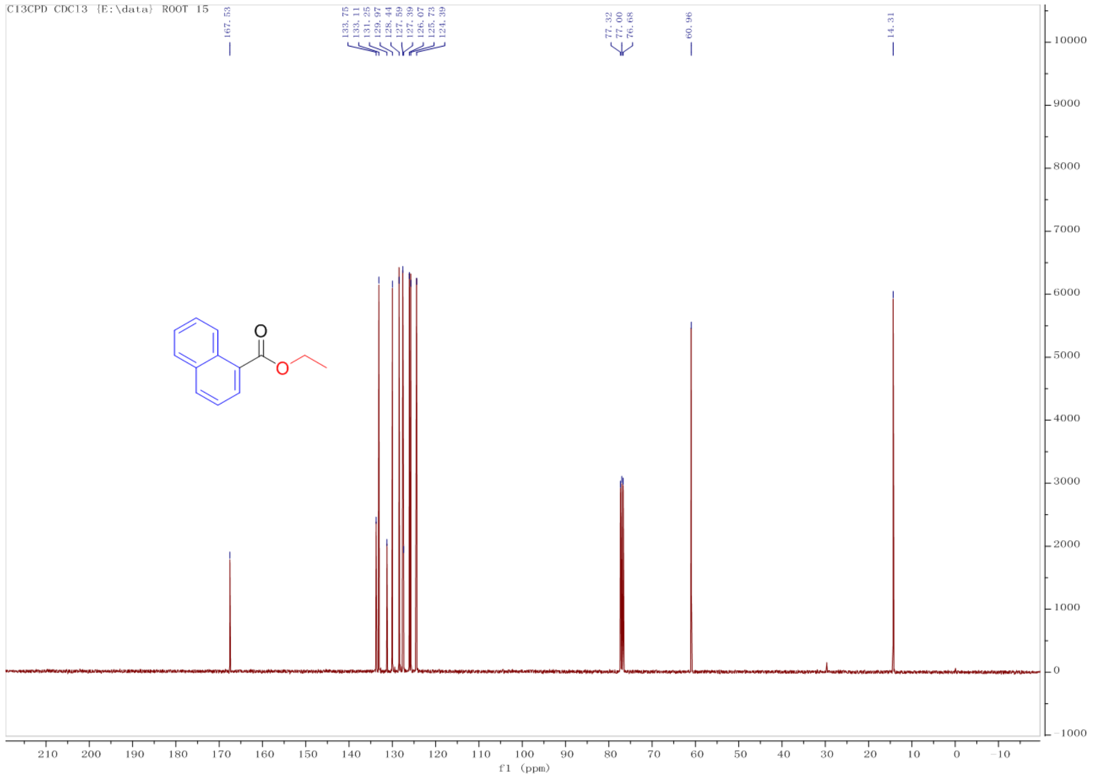
C13CPD CDCl3 {E:\data} ROOT 15
167.53
133.75
133.11
131.25
129.97
128.44
127.59
127.39
126.07
125.73
124.39
77.32
77.00
76.68
60.96
14.31
O
O
f1 (ppm)

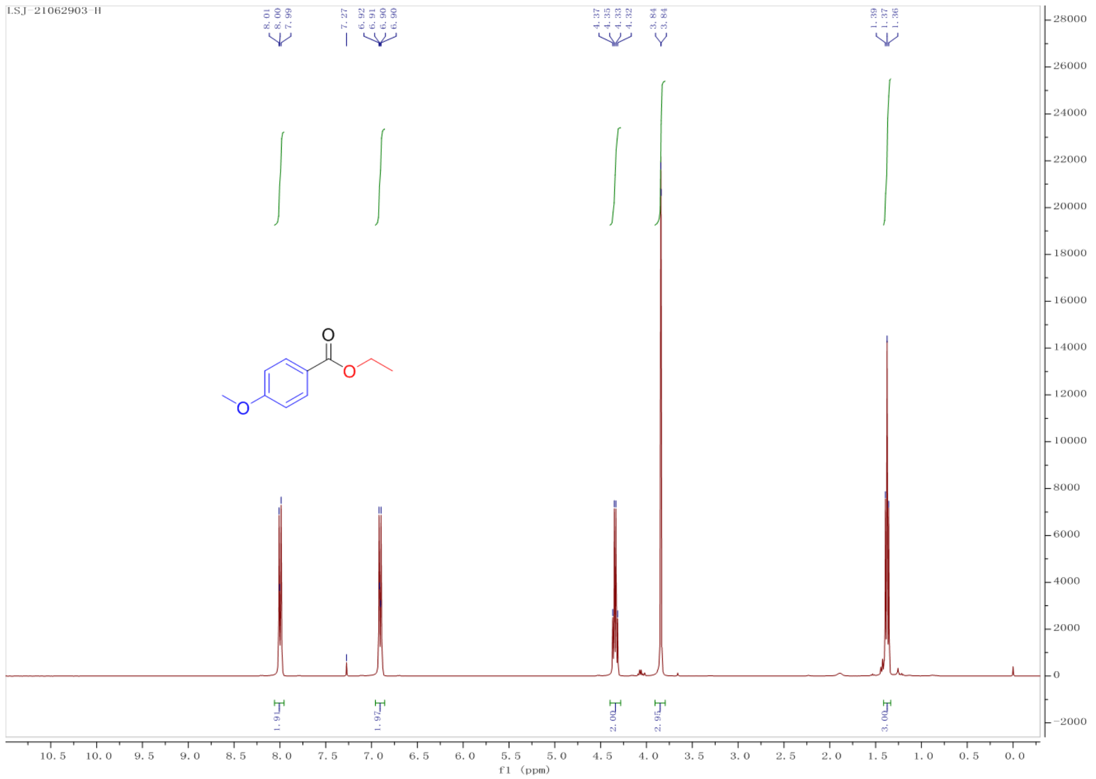
LSJ-21062903-H
8.01
8.00
7.99
7.27
6.92
6.91
6.90
6.90
4.37
4.35
4.33
4.32
3.84
3.84
1.39
1.37
1.36
1.91
1.97
2.00
2.95
3.00
f1 (ppm)

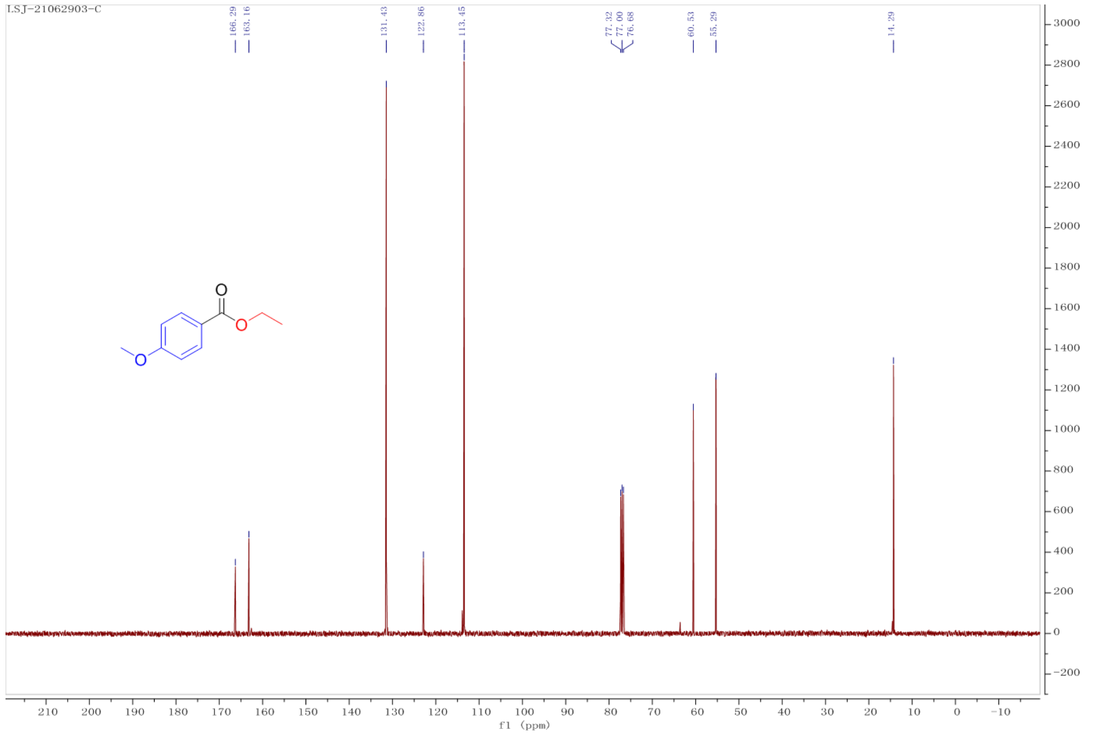
LSJ-21062903-C
166.29
163.16
131.43
122.86
113.45
77.32
77.00
76.68
60.53
55.29
14.29
f1 (ppm)

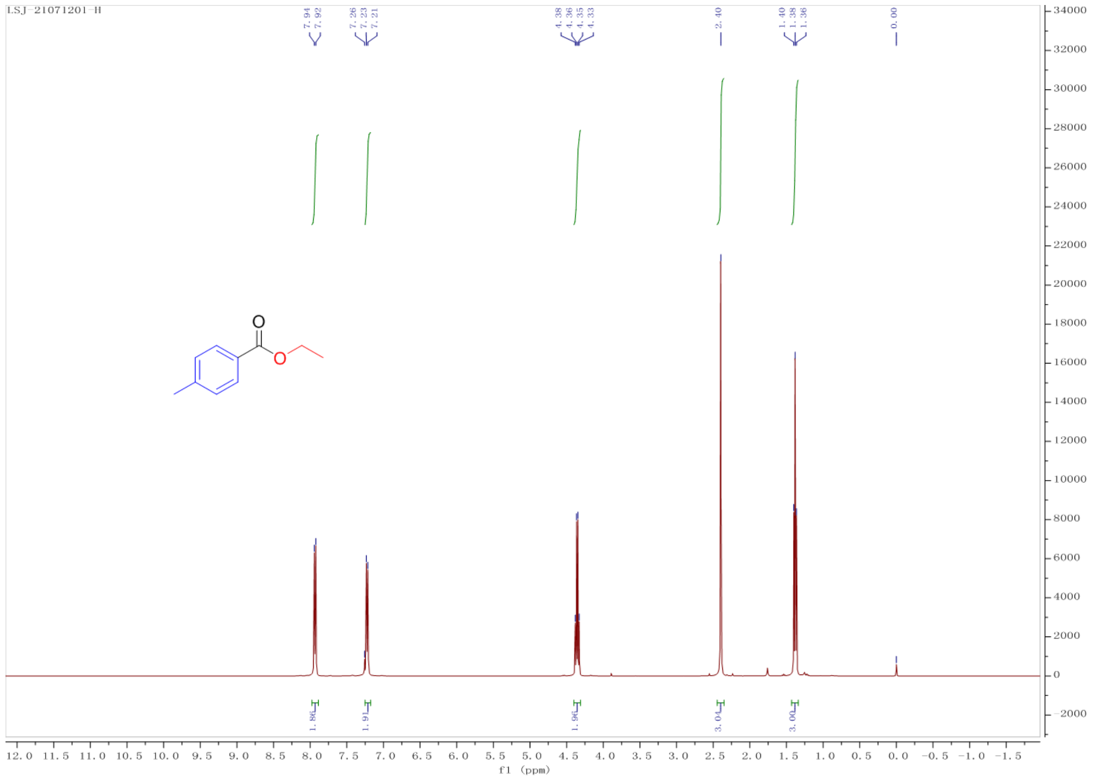
LSJ-21071201-H
7.94
7.92
7.26
7.23
7.21
4.38
4.36
4.35
4.33
2.40
1.40
1.38
1.36
0.00
1.86
1.91
1.96
3.04
3.00
f1 (ppm)

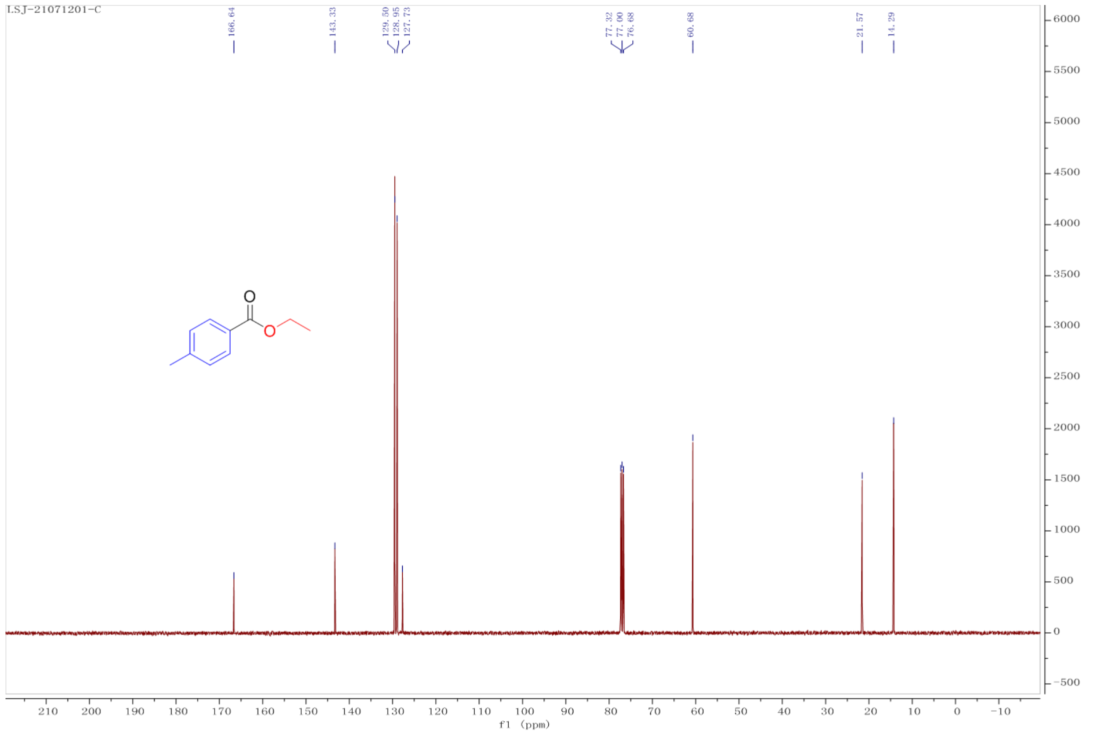
LSJ-21071201-C
166.64
143.33
129.50
128.95
127.73
77.32
77.00
76.68
60.68
21.57
14.29
f1 (ppm)

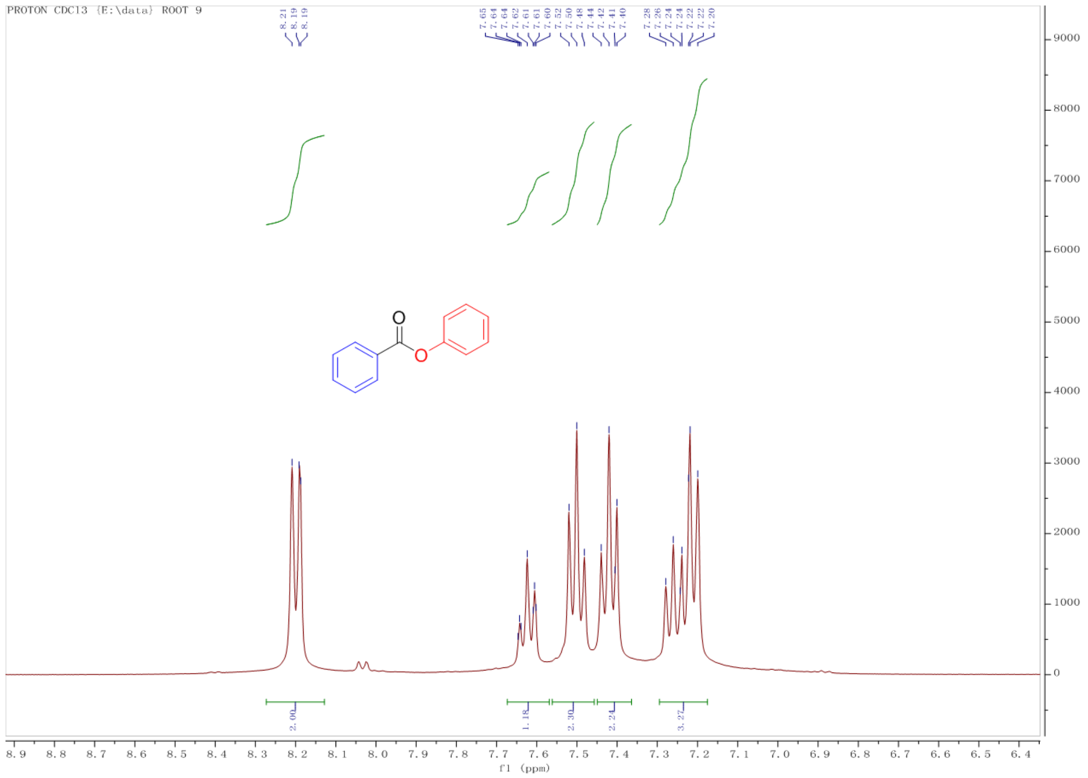
PROTON CDCl3 {E:\data} ROOT 9
8.21 8.19 8.19
7.65 7.64 7.62 7.61 7.61 7.60 7.52 7.50 7.48 7.44 7.42 7.41 7.40
7.28 7.26 7.24 7.24 7.22 7.22 7.20
O
O
2.00
1.18
2.30
2.24
3.27
f1 (ppm)

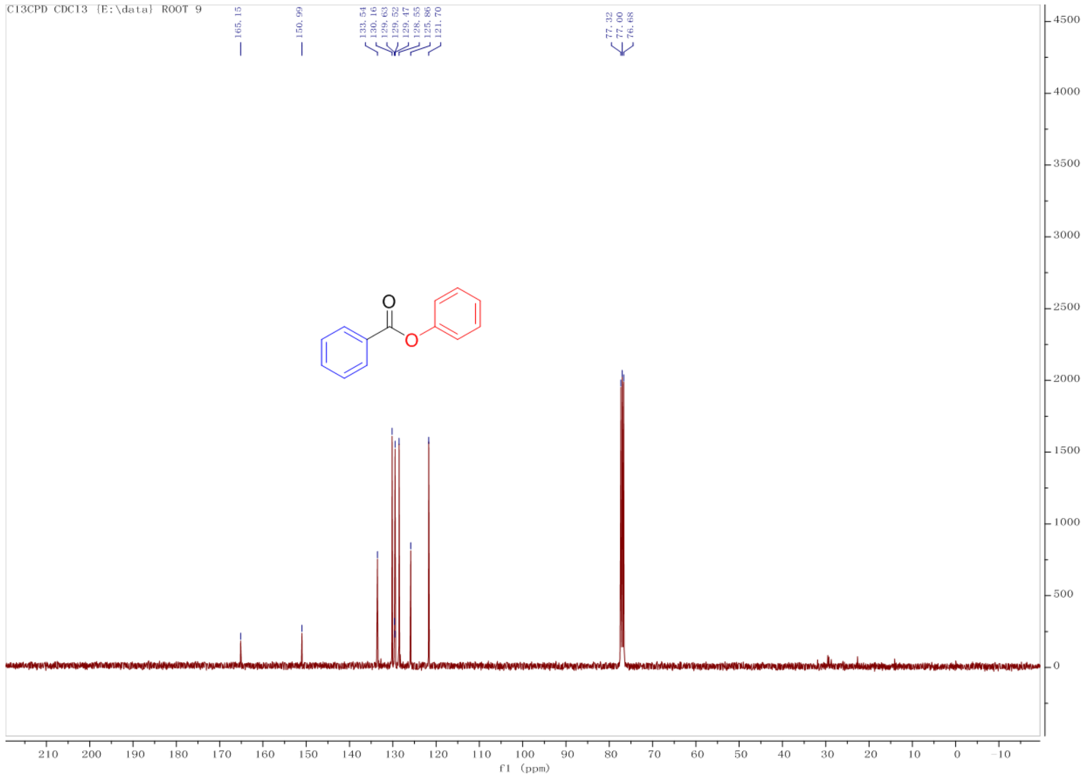
C13CPD CDCl3 {E:\data} ROOT 9
165.15
150.99
133.54 130.16 129.63 129.52 129.47 128.55 125.86 121.70
77.32 77.00 76.68
O
O
f1 (ppm)

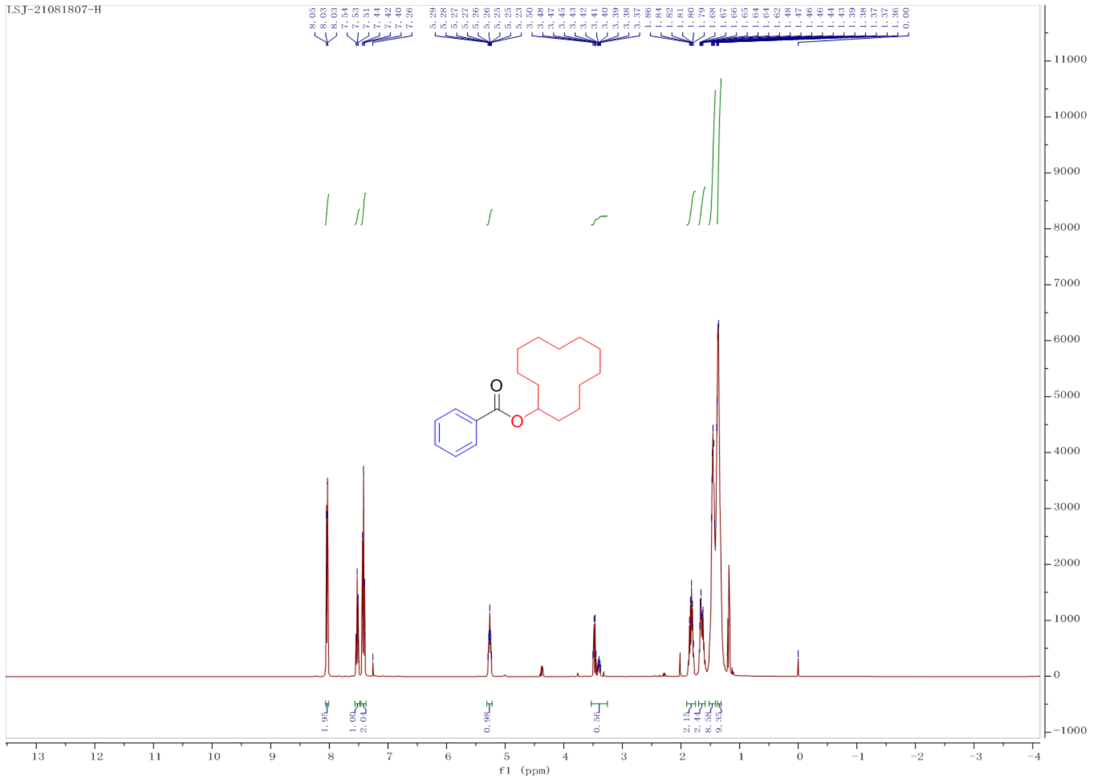
LSJ-21081807-H
8.05
8.03
8.03
7.54
7.53
7.51
7.44
7.42
7.40
7.26
5.29
5.28
5.27
5.27
5.26
5.26
5.25
5.25
5.25
5.23
3.50
3.48
3.47
3.45
3.43
3.42
3.41
3.40
3.39
3.38
3.37
1.86
1.84
1.82
1.81
1.80
1.79
1.68
1.67
1.66
1.65
1.64
1.64
1.62
1.48
1.47
1.46
1.46
1.44
1.43
1.39
1.38
1.37
1.37
1.36
0.00
1.95
1.00
2.01
0.98
0.56
2.15
2.14
8.58
9.35
O
O
f1 (ppm)

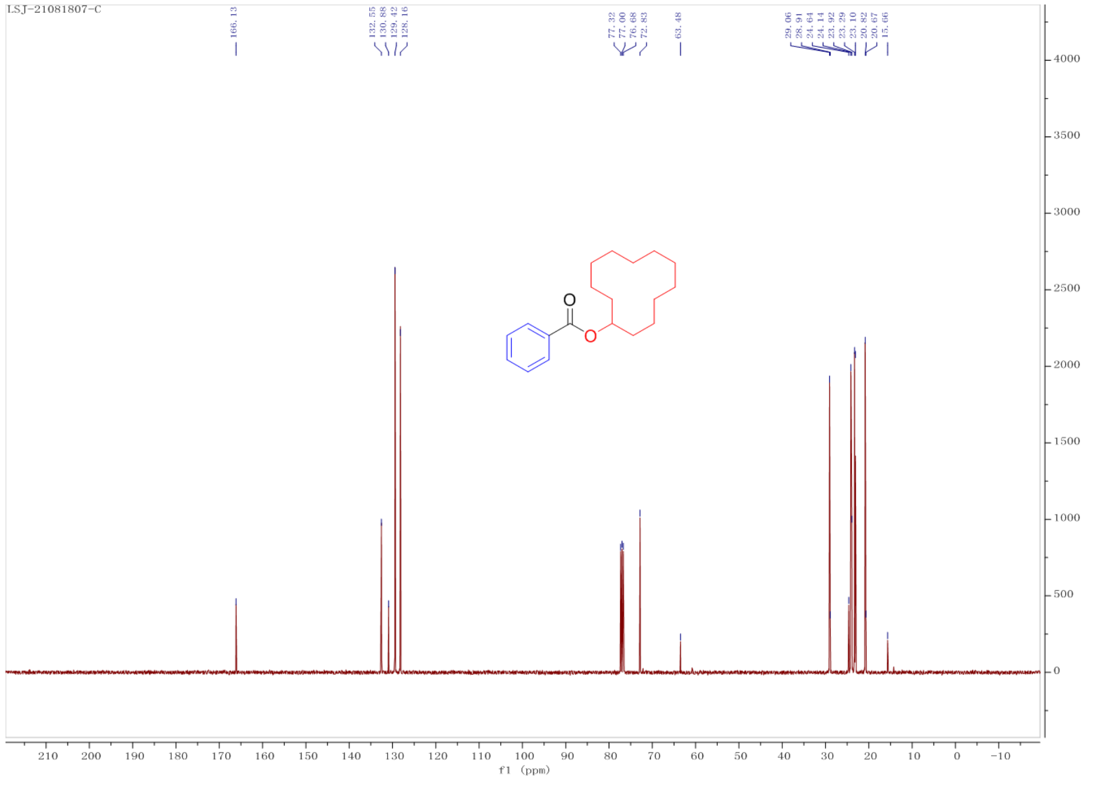
LSJ-21081807-C
166.13
132.55
130.88
129.42
128.16
77.32
77.00
76.68
72.83
63.48
29.06
28.91
24.64
24.14
23.92
23.29
23.10
20.82
20.67
15.66
O
O
f1 (ppm)

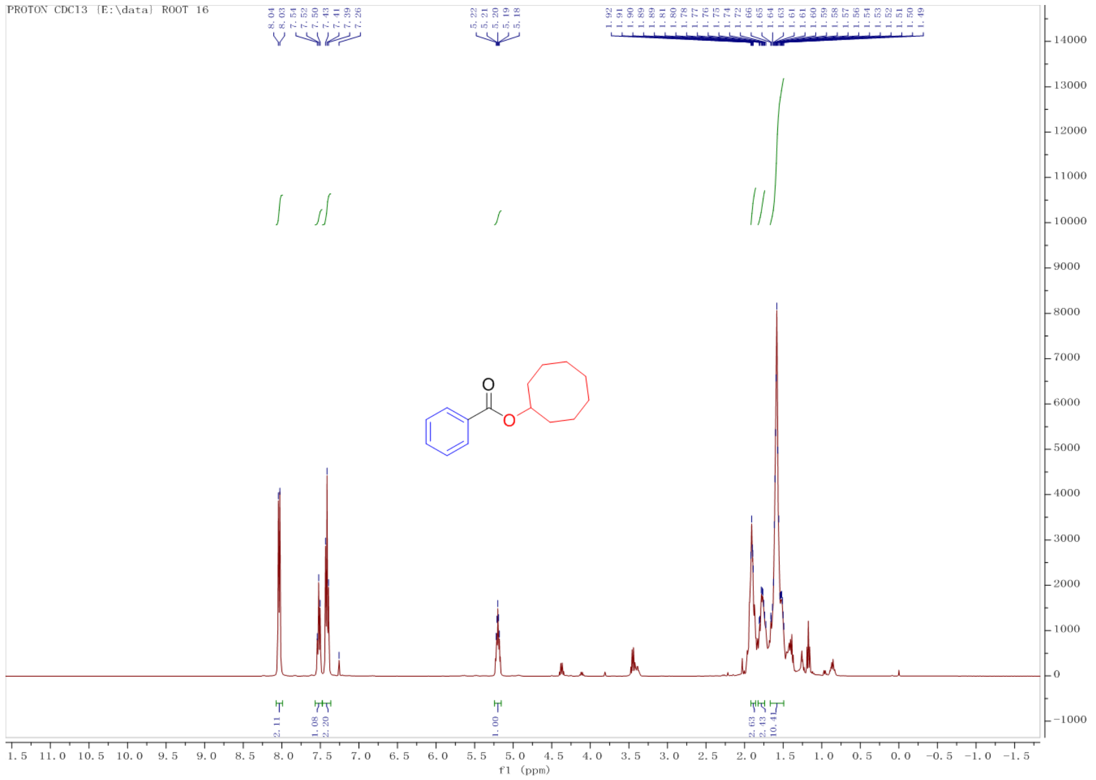
PROTON CDCl3 {E:\data} ROOT 16
f1 (ppm)

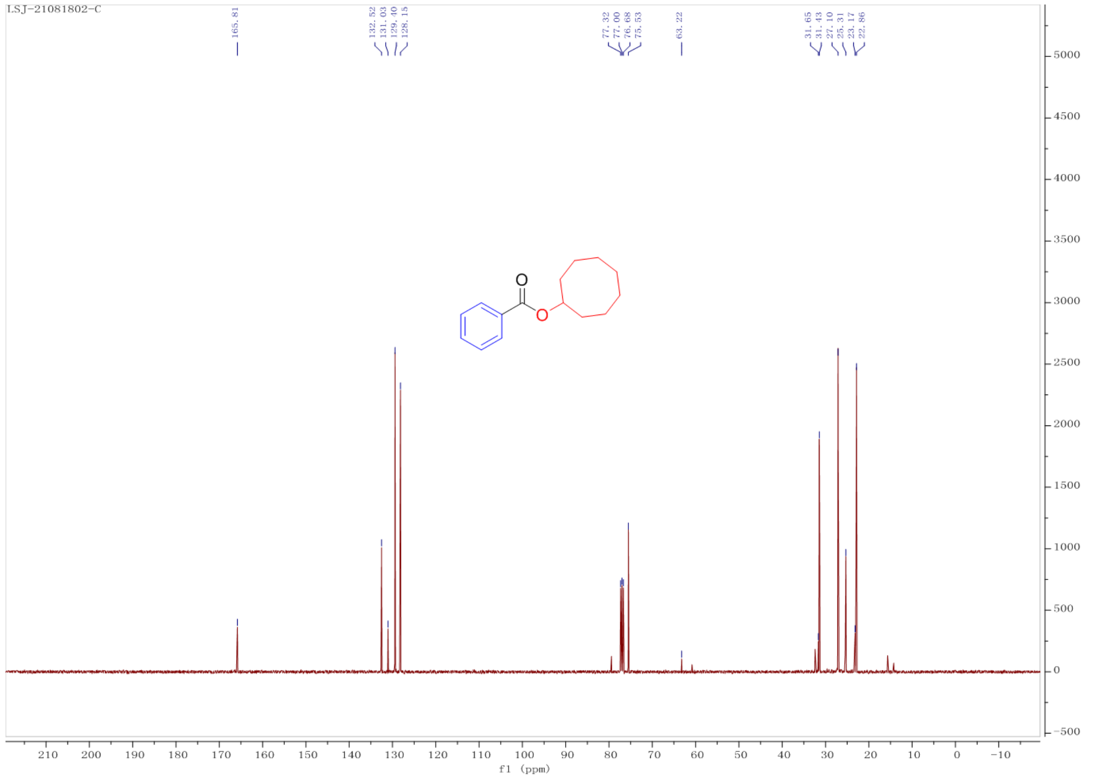
LSJ-21081802-C
165.81
132.52
131.03
129.40
128.15
77.32
77.00
76.68
75.53
63.22
31.65
31.43
27.10
25.31
23.17
22.86
f1 (ppm)

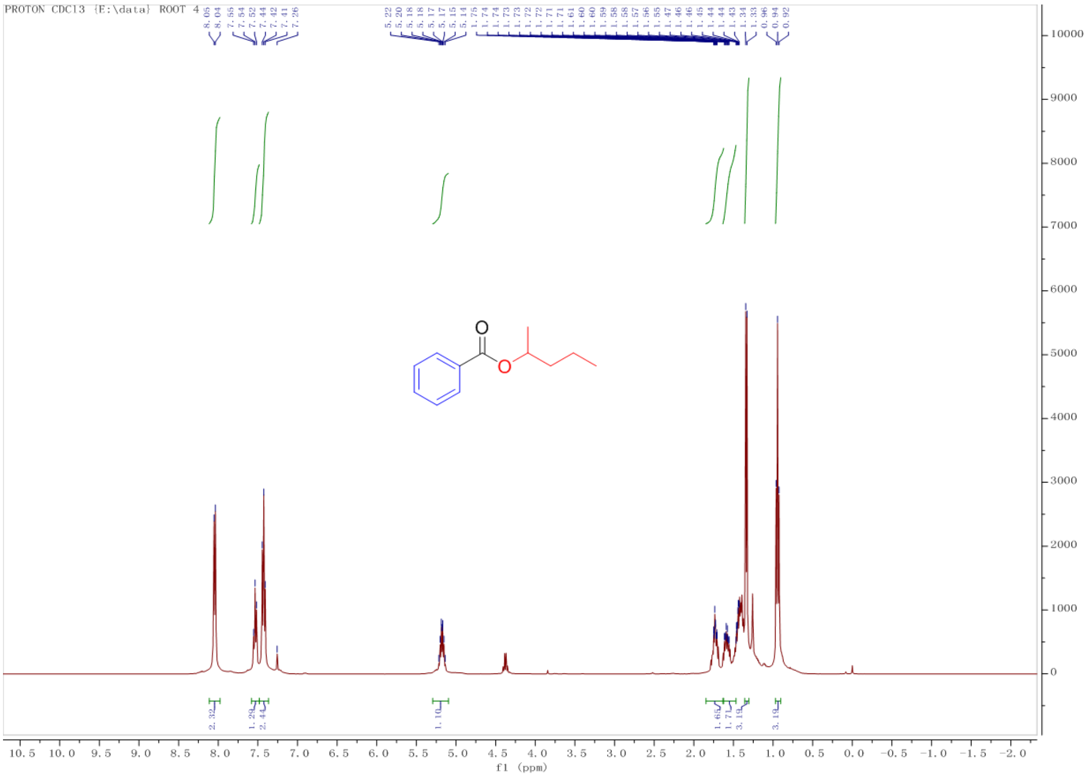
PROTON CDCl3 {E:\data} ROOT 4
f1 (ppm)

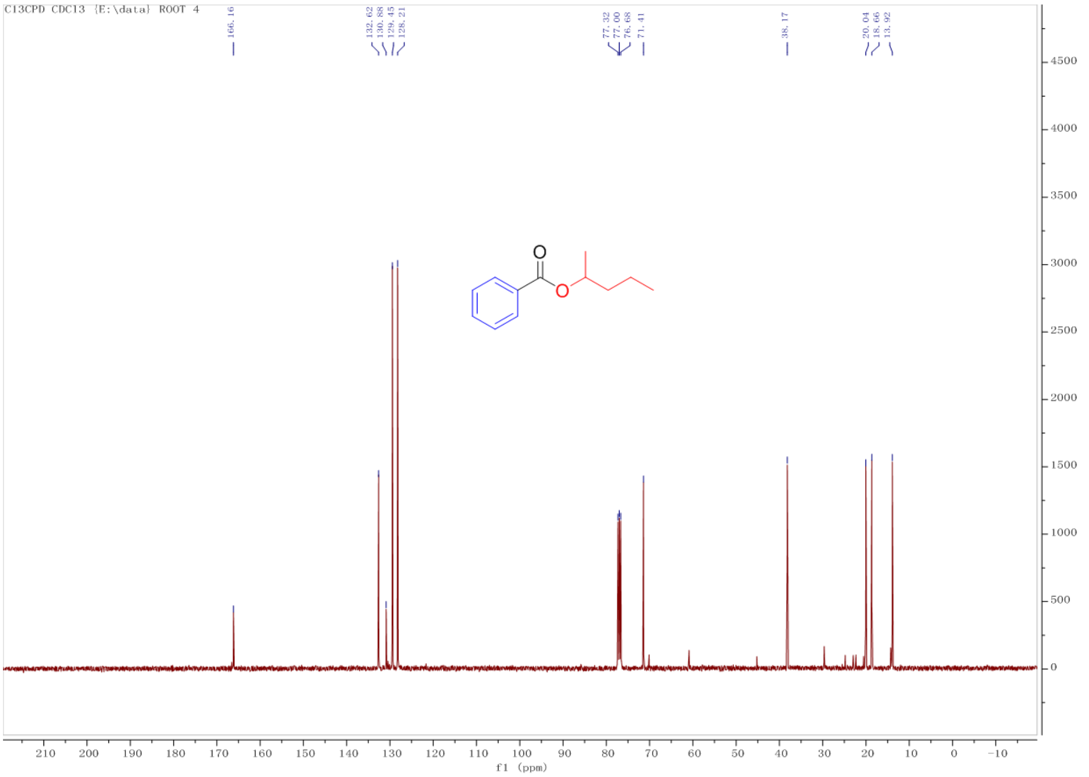
C13CPD CDCl3 {E:\data} ROOT 4
166.16
132.62
130.88
129.45
128.21
77.32
77.00
76.68
71.41
38.17
20.04
18.66
13.92
f1 (ppm)

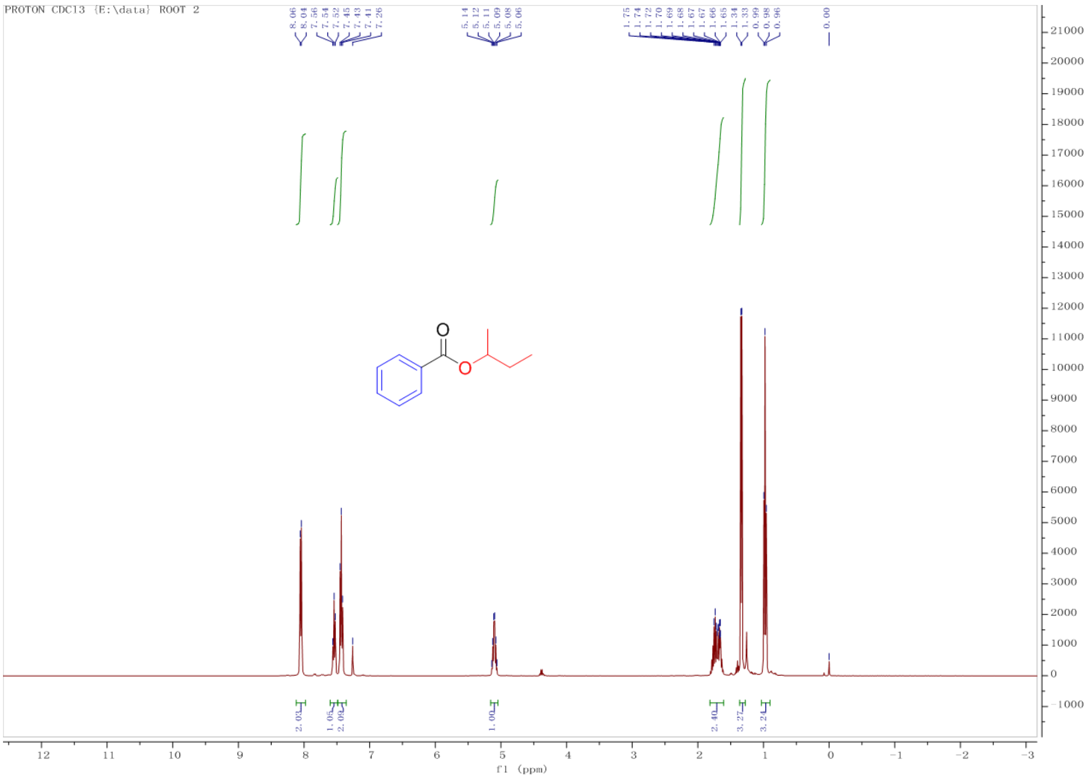
PROTON CDCl3 {E:\data} ROOT 2
O
O
f1 (ppm)

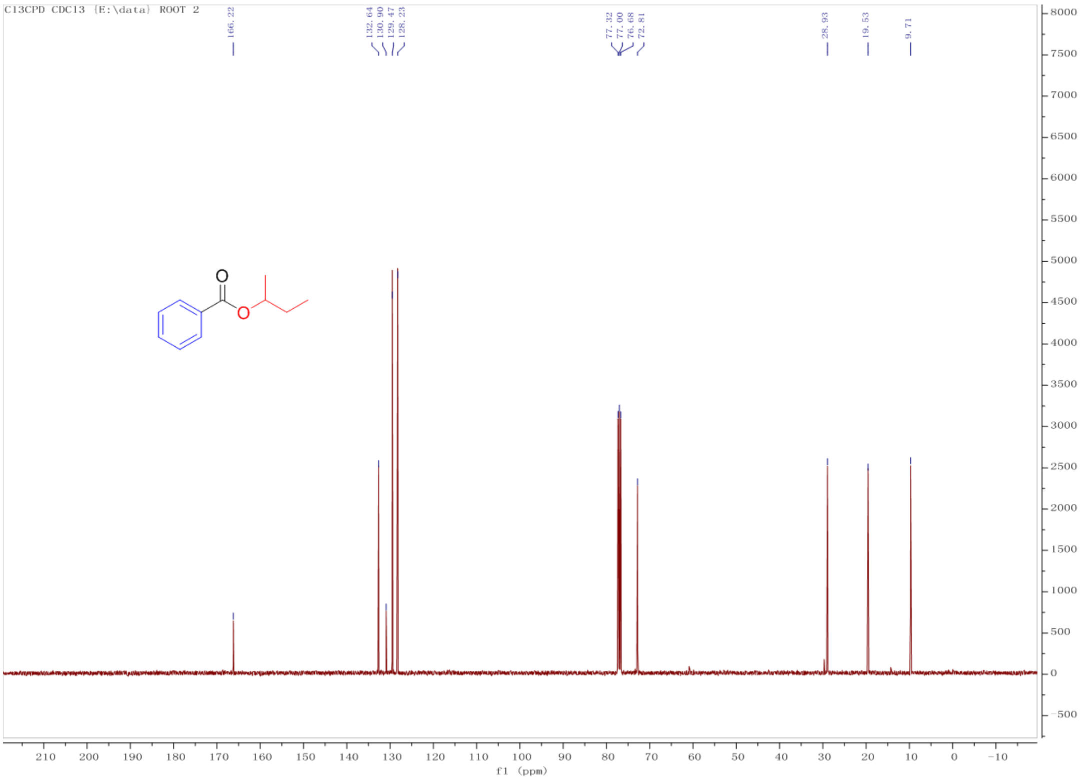
C13CPD CDCl3 {E:\data} ROOT 2
O
O
f1 (ppm)

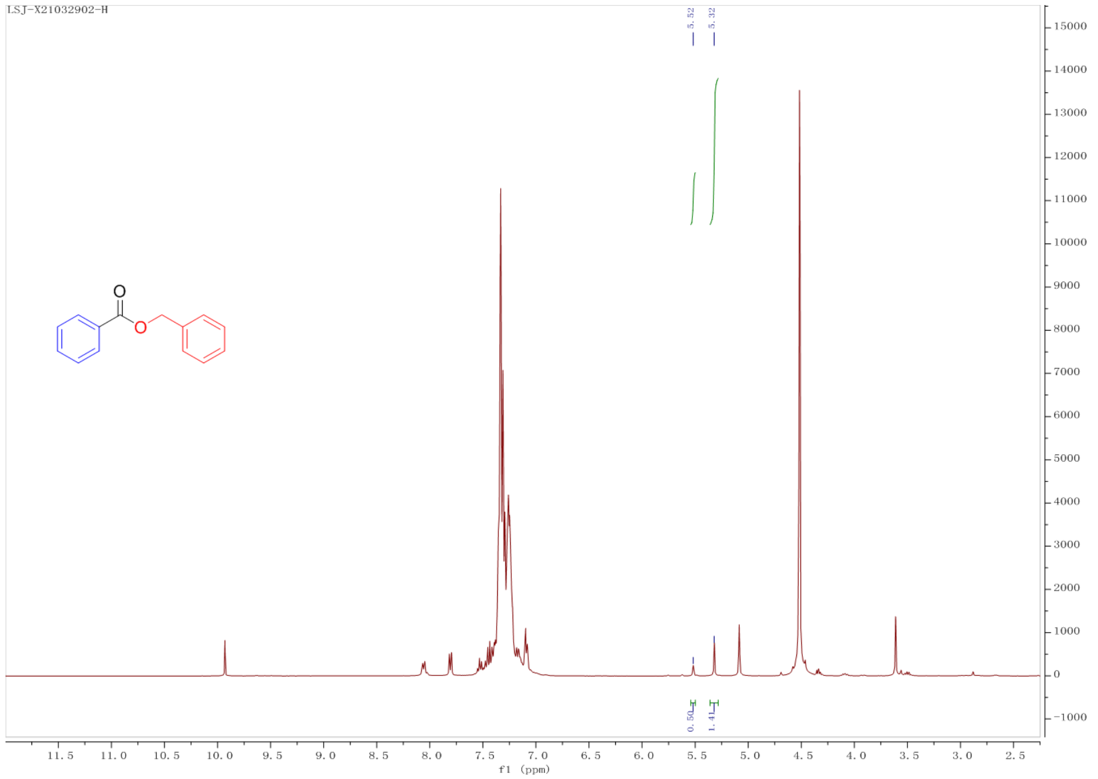
LSJ-X21032902-H
5.52
5.32
0.50
1.41
f1 (ppm)

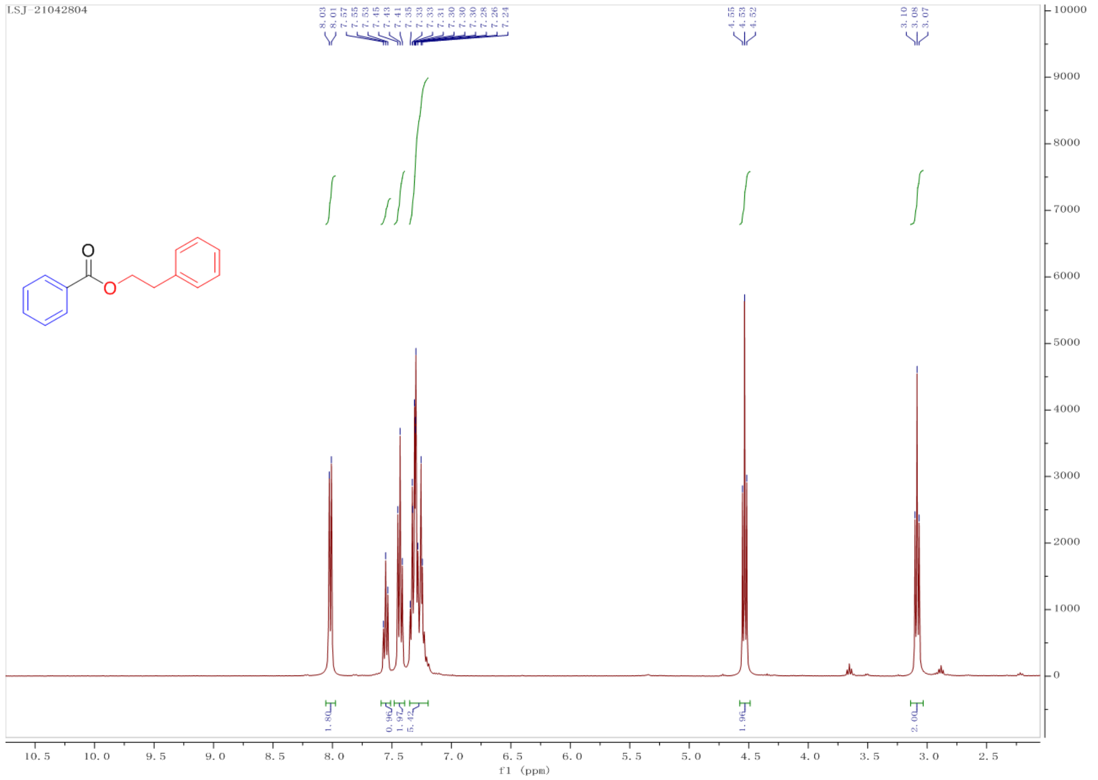
LSJ-21042804
8.03
8.01
7.57
7.55
7.53
7.45
7.43
7.41
7.35
7.33
7.31
7.30
7.30
7.30
7.28
7.26
7.24
4.55
4.53
4.52
3.10
3.08
3.07
1.80
0.96
1.97
5.42
1.96
2.00
f1 (ppm)

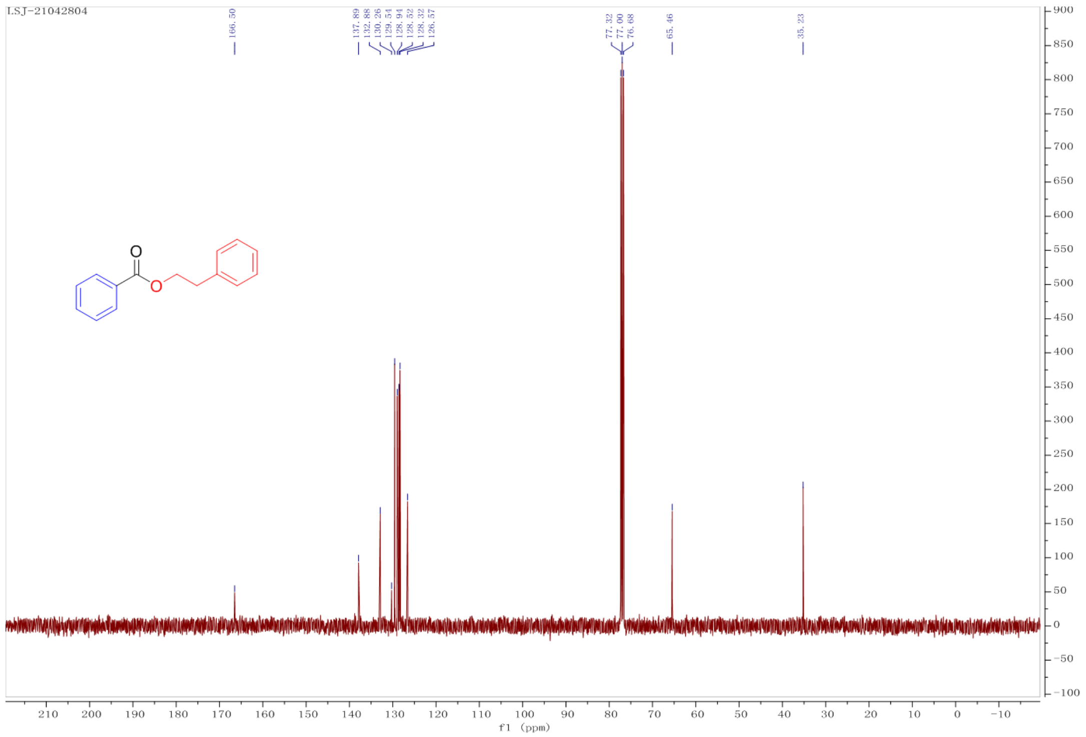
LSJ-21042804
166.50
137.89
132.88
130.26
129.54
128.94
128.52
128.32
126.57
77.32
77.00
76.68
65.46
35.23
f1 (ppm)

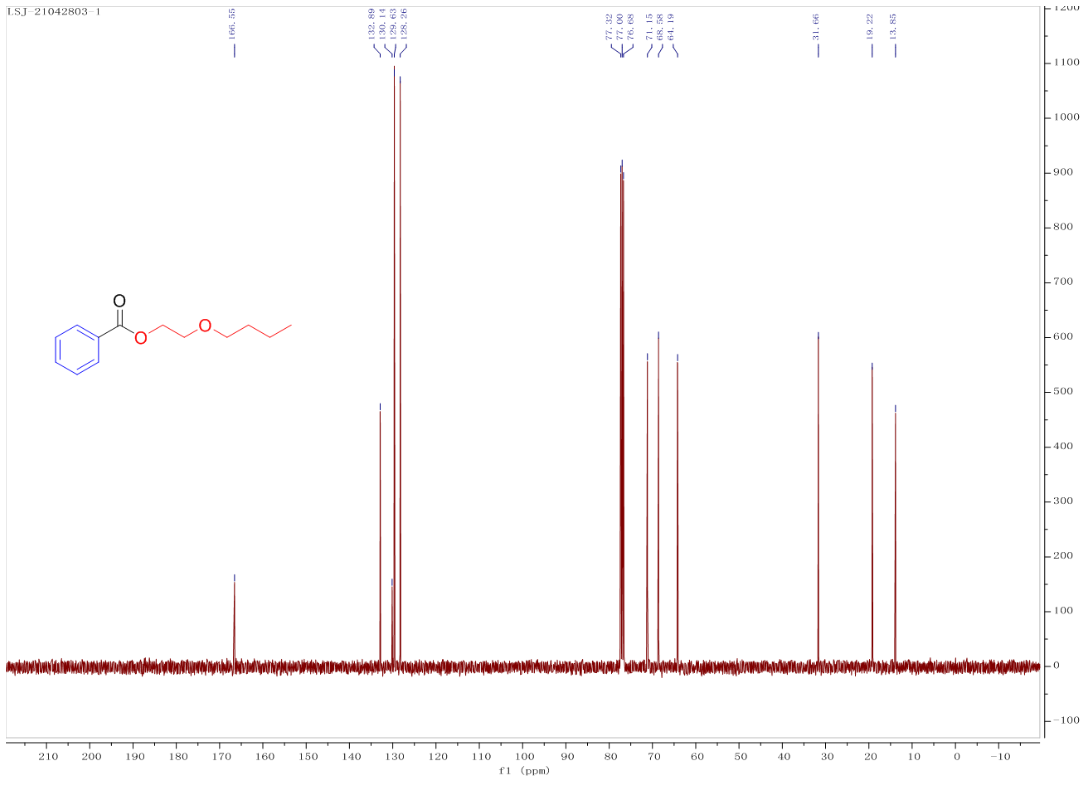
LSJ-21042803-1
166.55
132.89
130.14
129.63
128.26
77.32
77.00
76.68
71.15
68.58
64.19
31.66
19.22
13.85
f1 (ppm)

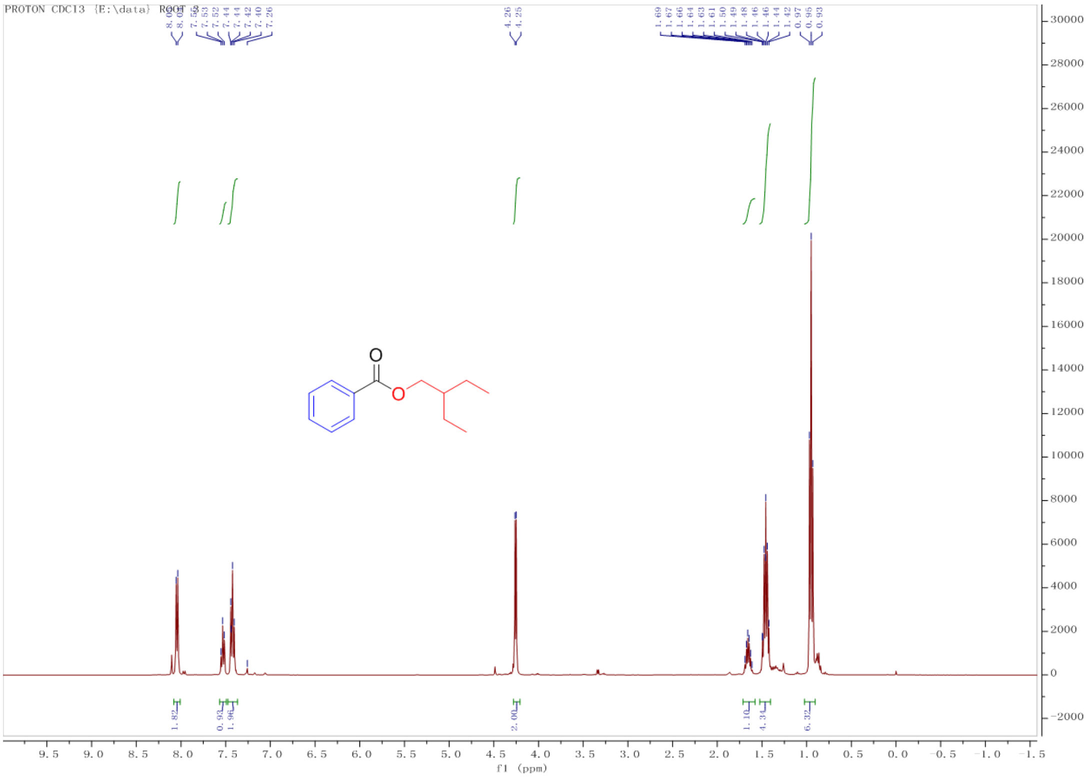
PROTON CDCl3 {E:\data} ROOT 3
O
O
f1 (ppm)

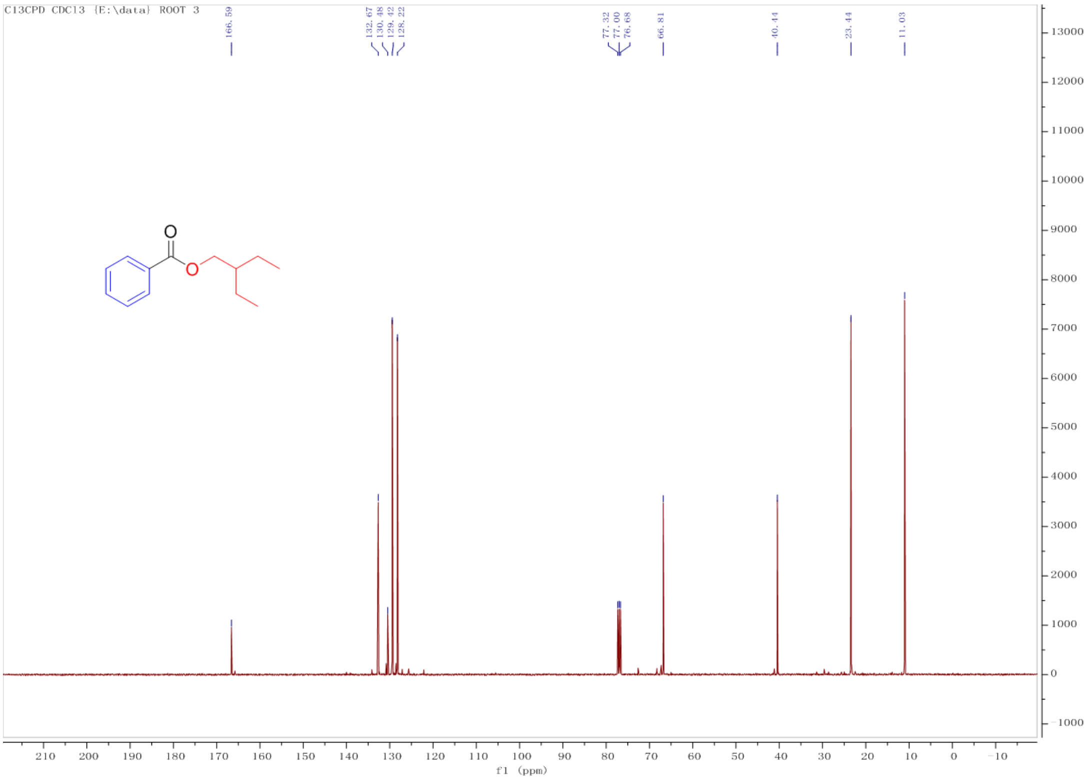
C13CPD CDCl3 {E:\data} ROOT 3
166.59
132.67
130.48
129.42
128.22
77.32
77.00
76.68
66.81
40.44
23.44
11.03
O
O
f1 (ppm)

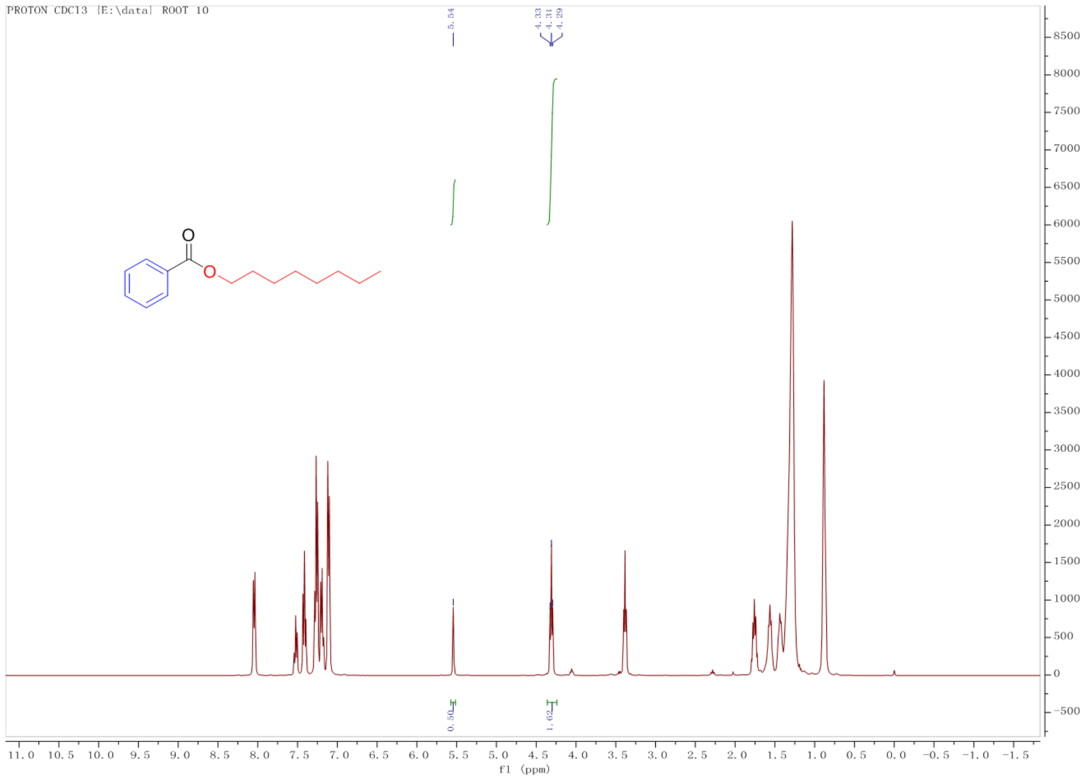
PROTON CDCl3 {E:\data} ROOT 10
f1 (ppm)

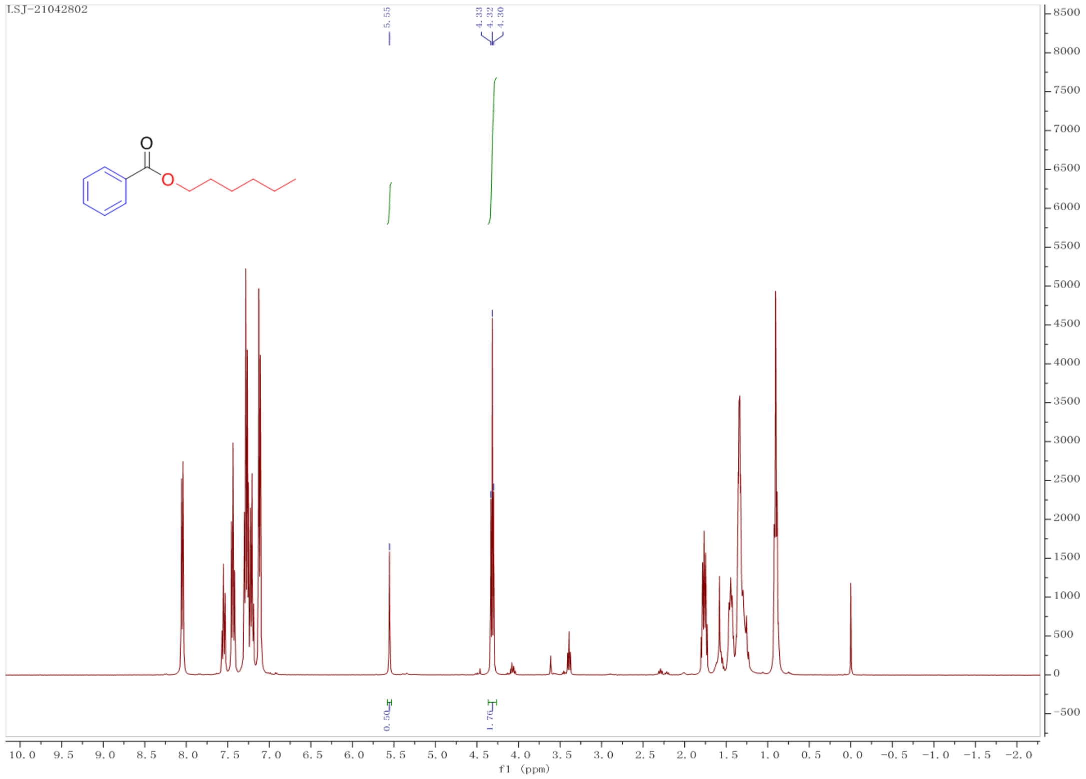
LSJ-21042802
f1 (ppm)

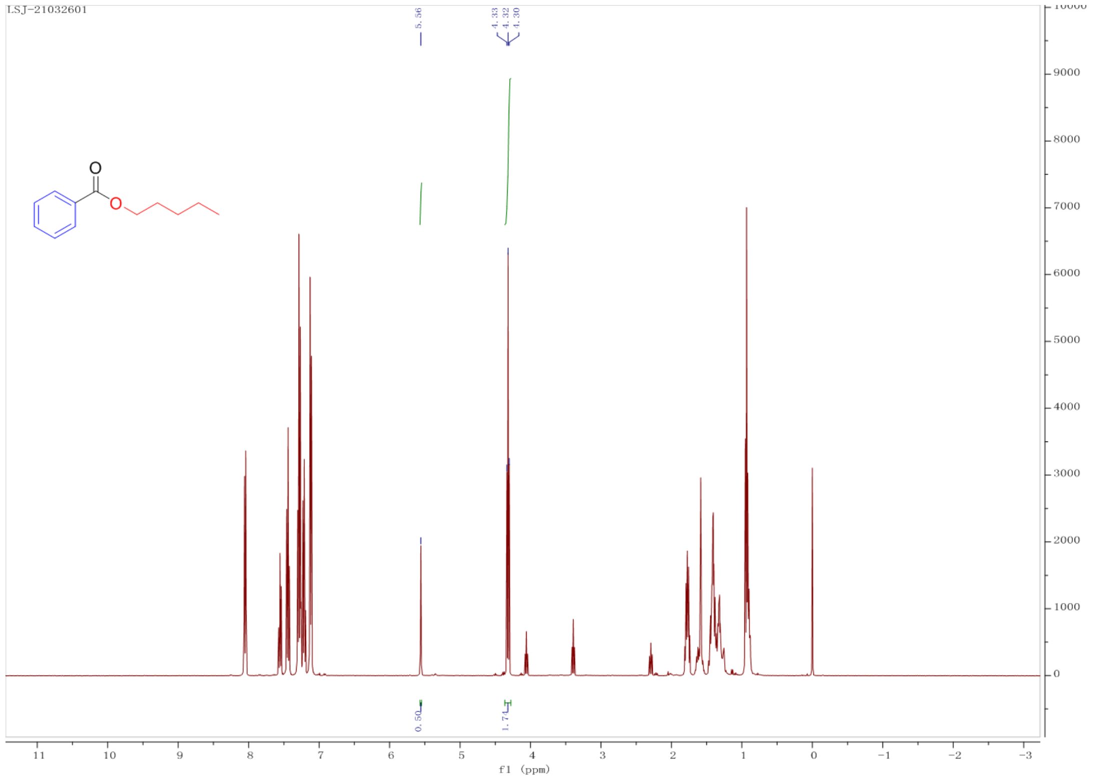
LSJ-21032601
f1 (ppm)

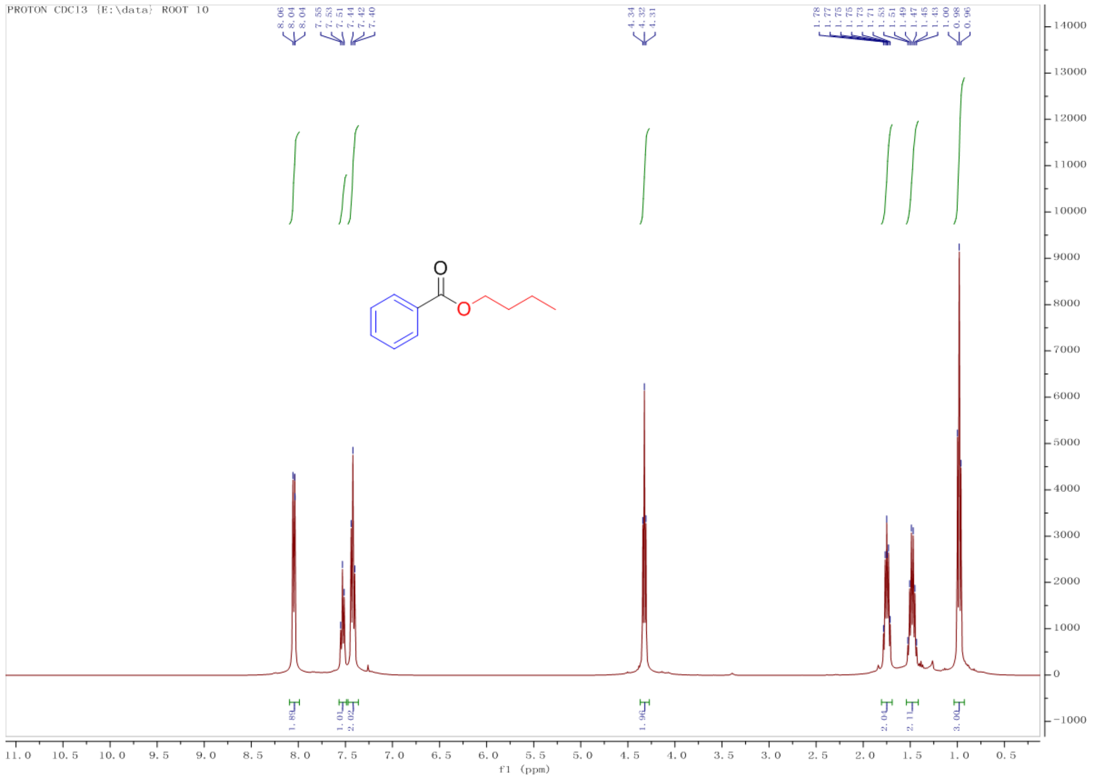
PROTON CDCl3 {E:\data} ROOT 10
f1 (ppm)

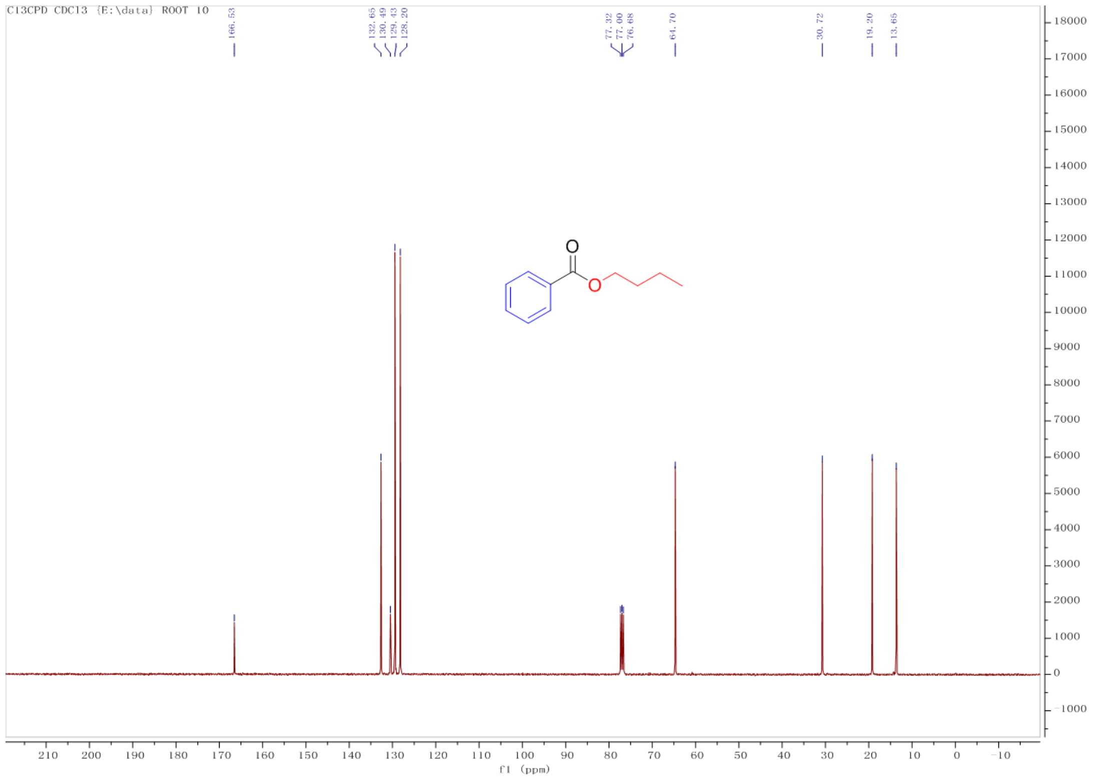

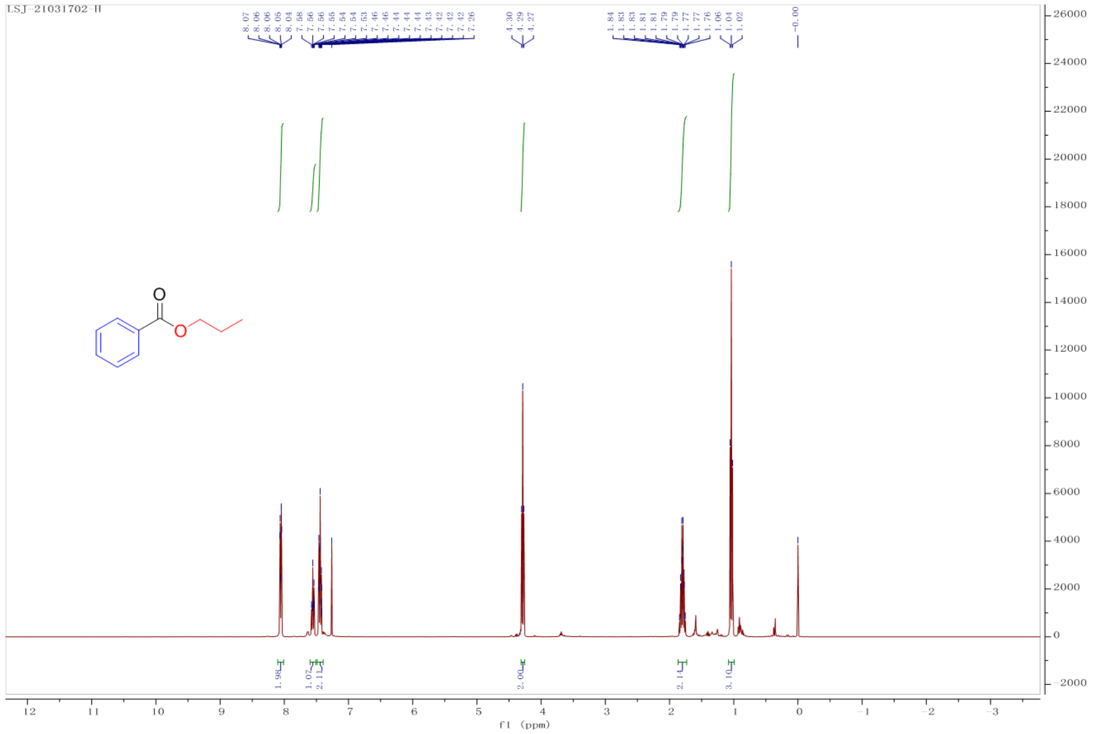

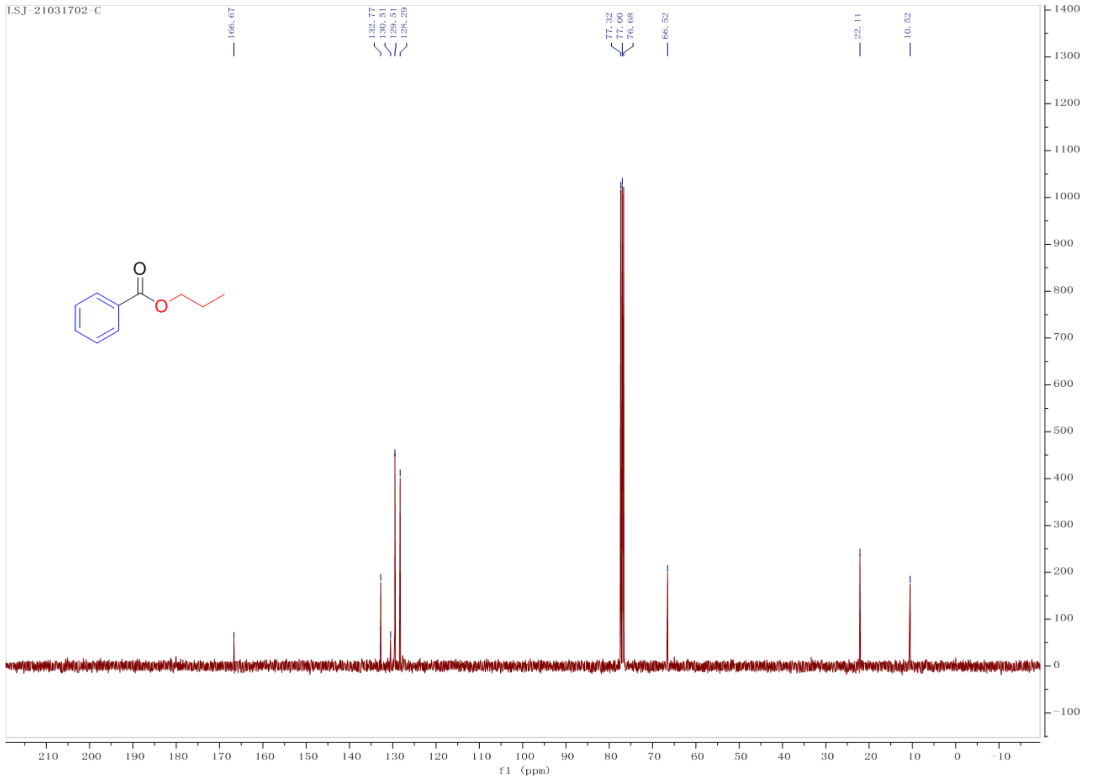
LSJ-21031702-C
166.67
132.77
130.51
129.51
128.29
77.32
77.00
76.68
66.52
22.11
10.52
O
O
f1 (ppm)

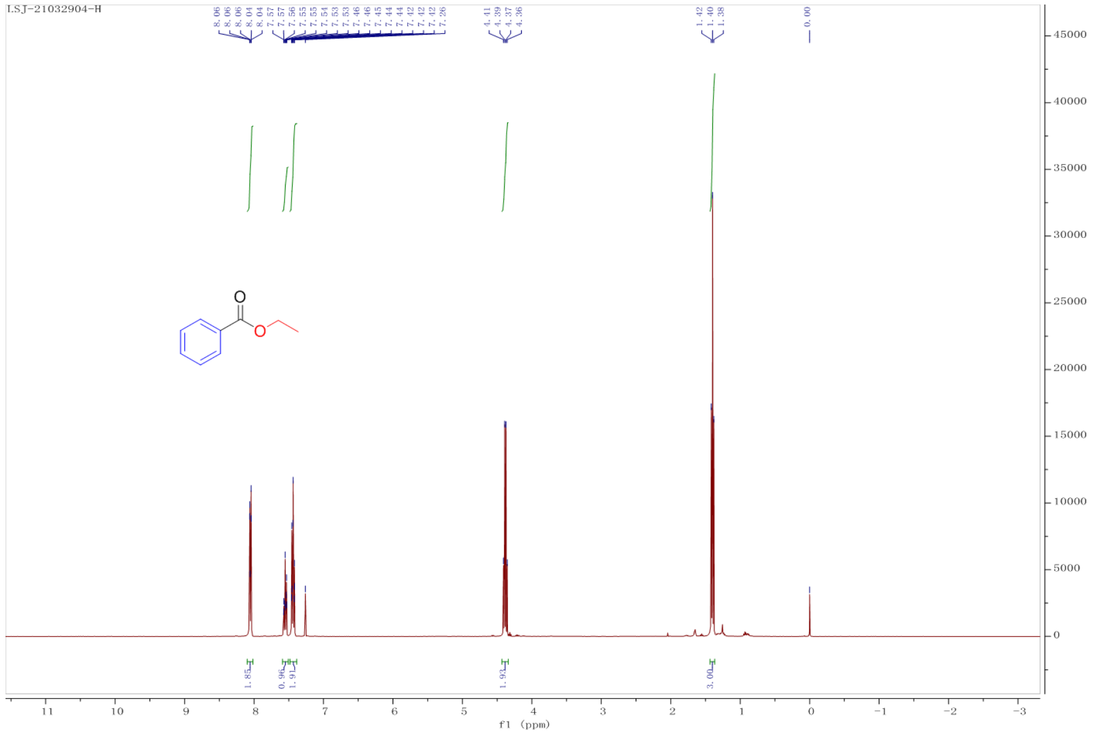
LSJ-21032904-H
8.06
8.06
8.06
8.04
8.04
7.57
7.56
7.55
7.55
7.54
7.53
7.53
7.46
7.46
7.45
7.44
7.44
7.42
7.42
7.42
7.26
4.41
4.39
4.37
4.36
1.42
1.40
1.38
0.00
1.85
0.96
1.91
1.93
3.00
O
O
f1 (ppm)

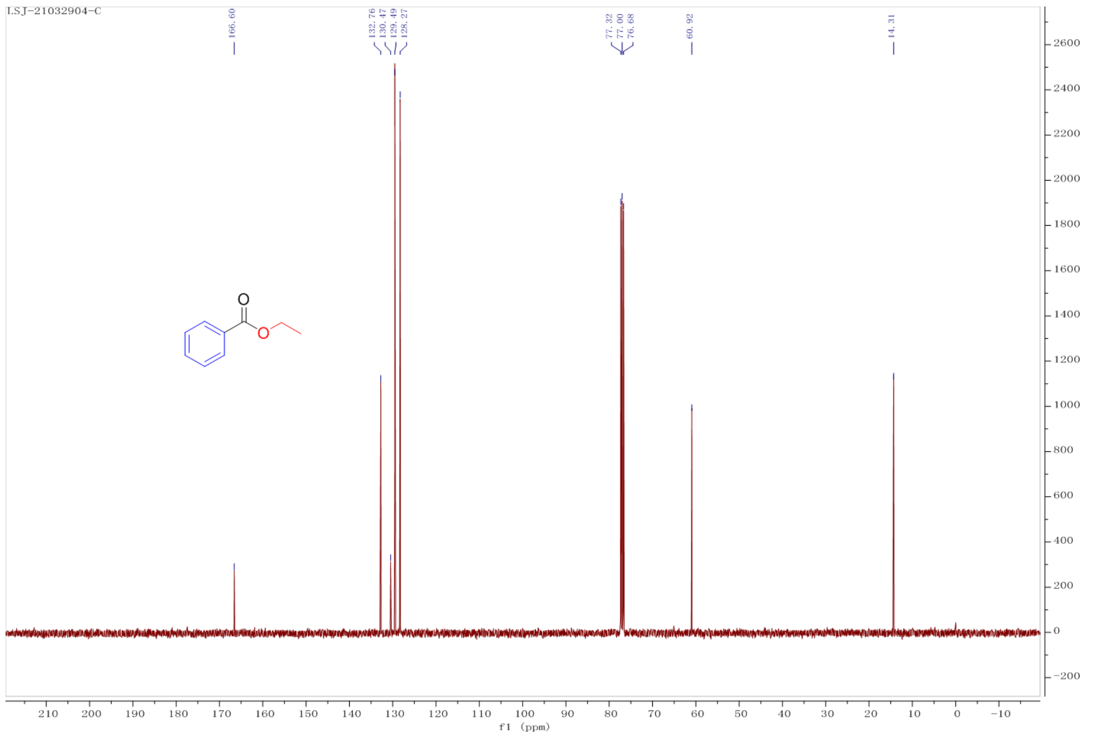
LSJ-21032904-C
166.60
132.76
130.47
129.49
128.27
77.32
77.00
76.68
60.92
14.31
f1 (ppm)

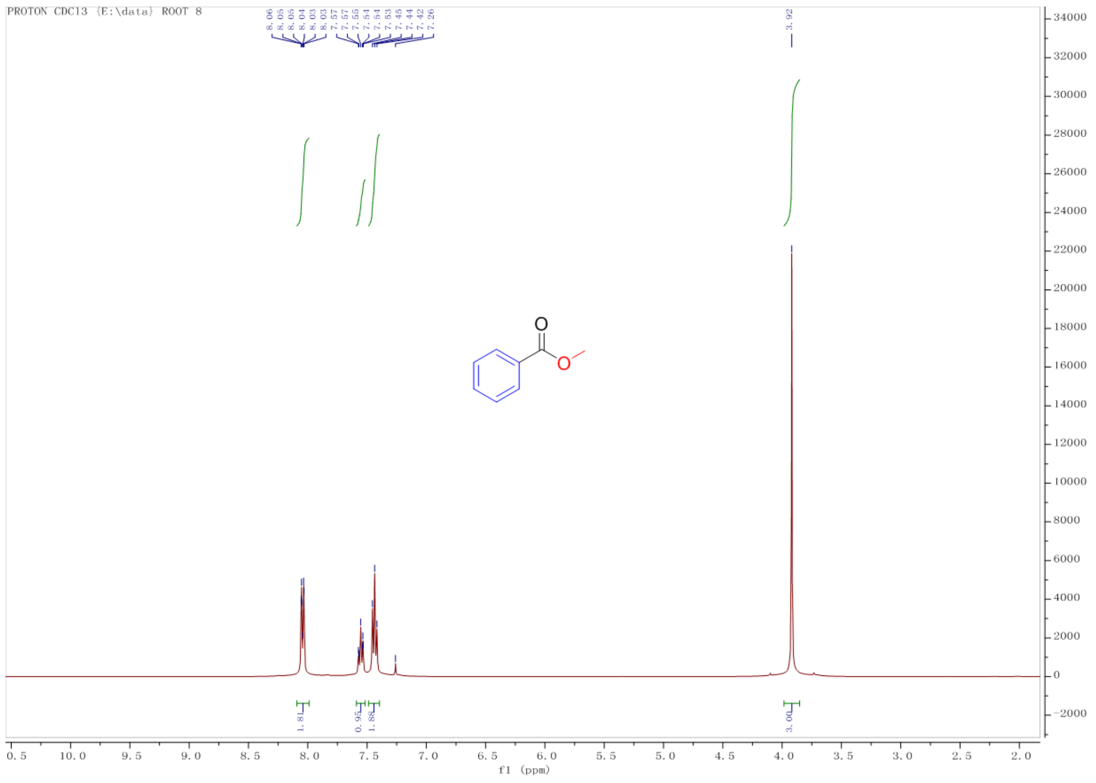
PROTON CDCl3 {E:\data} ROOT 8
3.92
1.81
0.95
1.88
3.00
f1 (ppm)

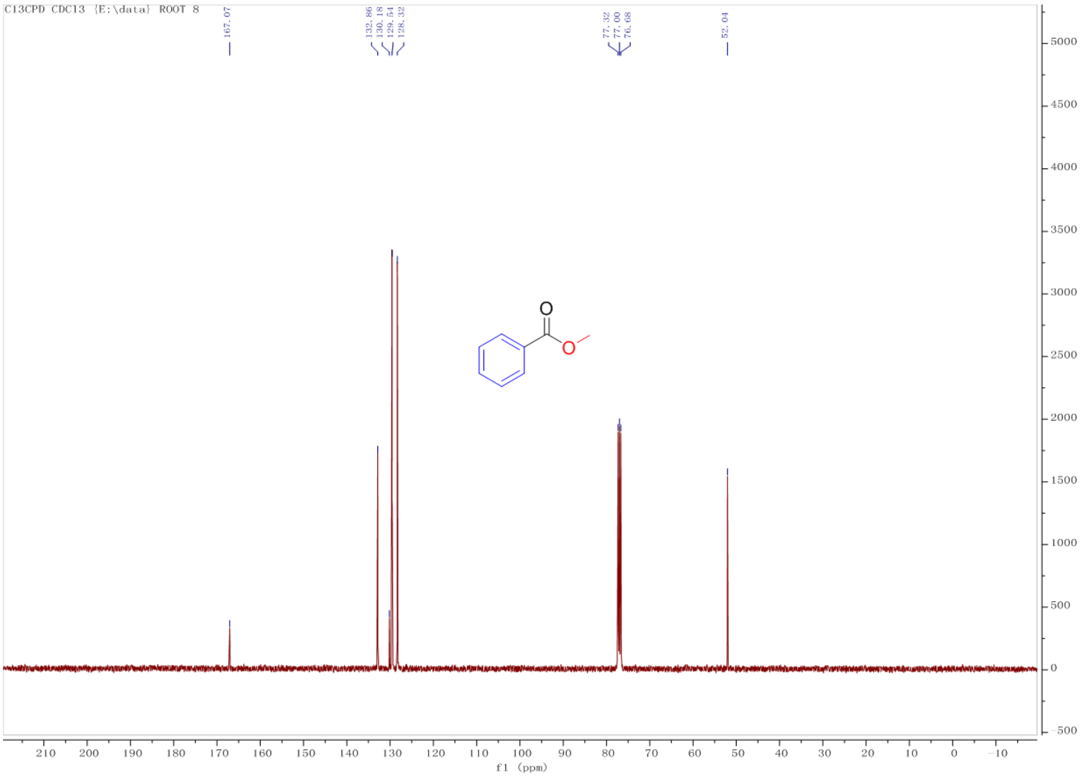
C13CPD CDCl3 {E:\data} ROOT 8
167.07
132.86
130.18
129.54
128.32
77.32
77.00
76.68
52.04
O
O
f1 (ppm)

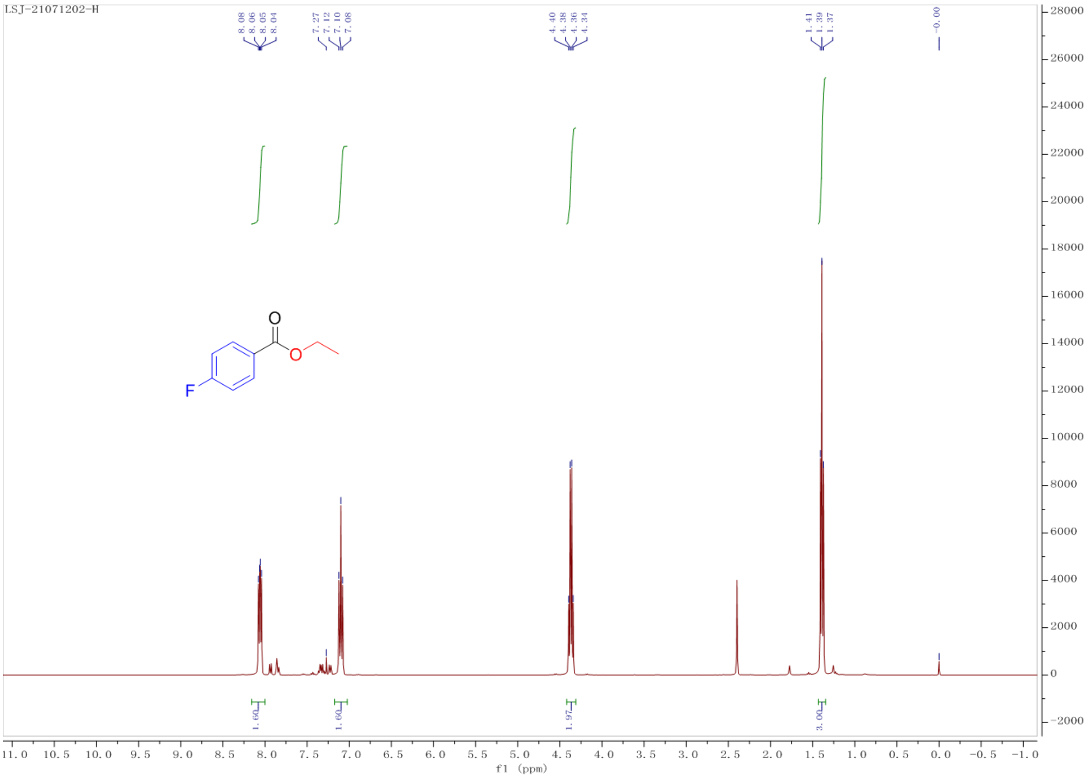
LSJ-21071202-H
8.08
8.06
8.05
8.04
7.27
7.12
7.10
7.08
4.40
4.38
4.36
4.34
1.41
1.39
1.37
-0.00
O
O
F
1.60
1.60
1.97
3.00
f1 (ppm)

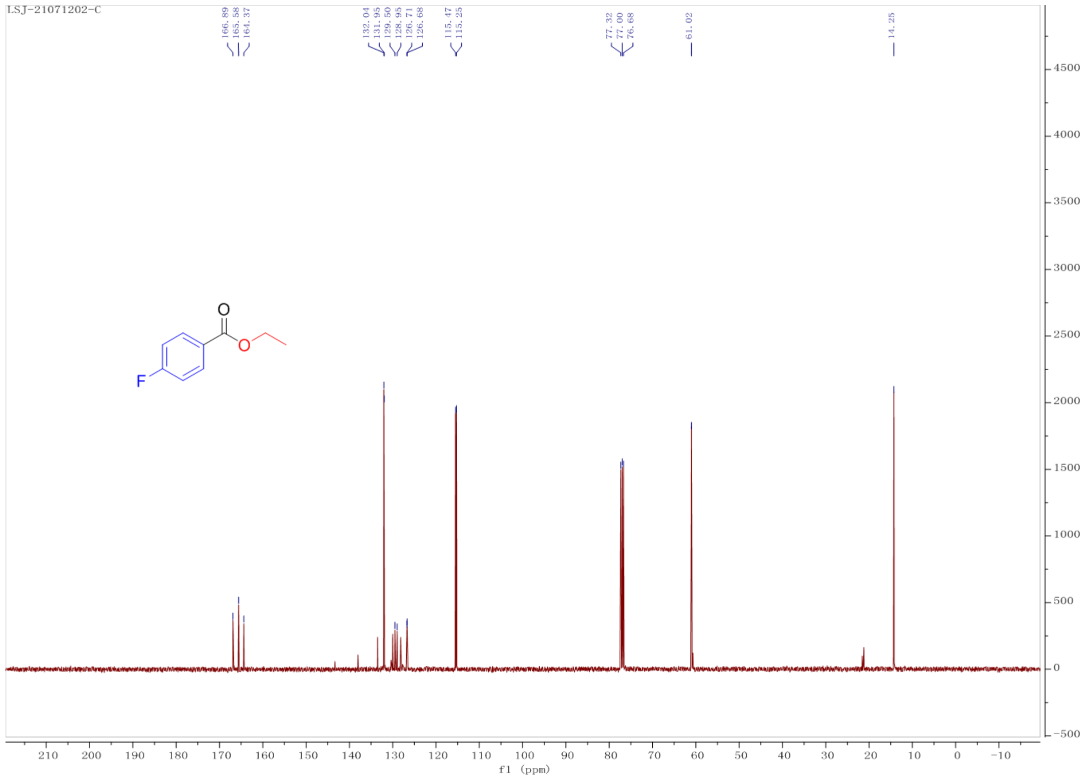
LSJ-21071202-C
166.89
165.58
164.37
132.04
131.95
129.50
128.95
126.71
126.68
115.47
115.25
77.32
77.00
76.68
61.02
14.25
O
O
F
f1 (ppm)

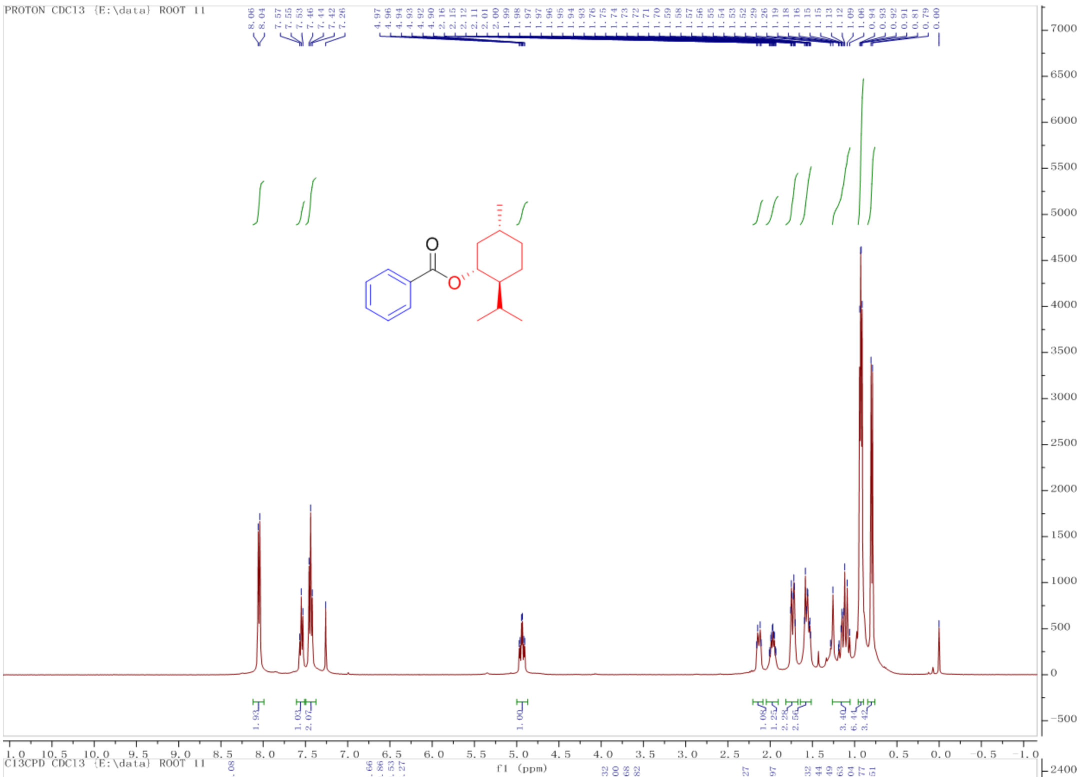
PROTON CDCl3 {E:\data} ROOT 11
f1 (ppm)

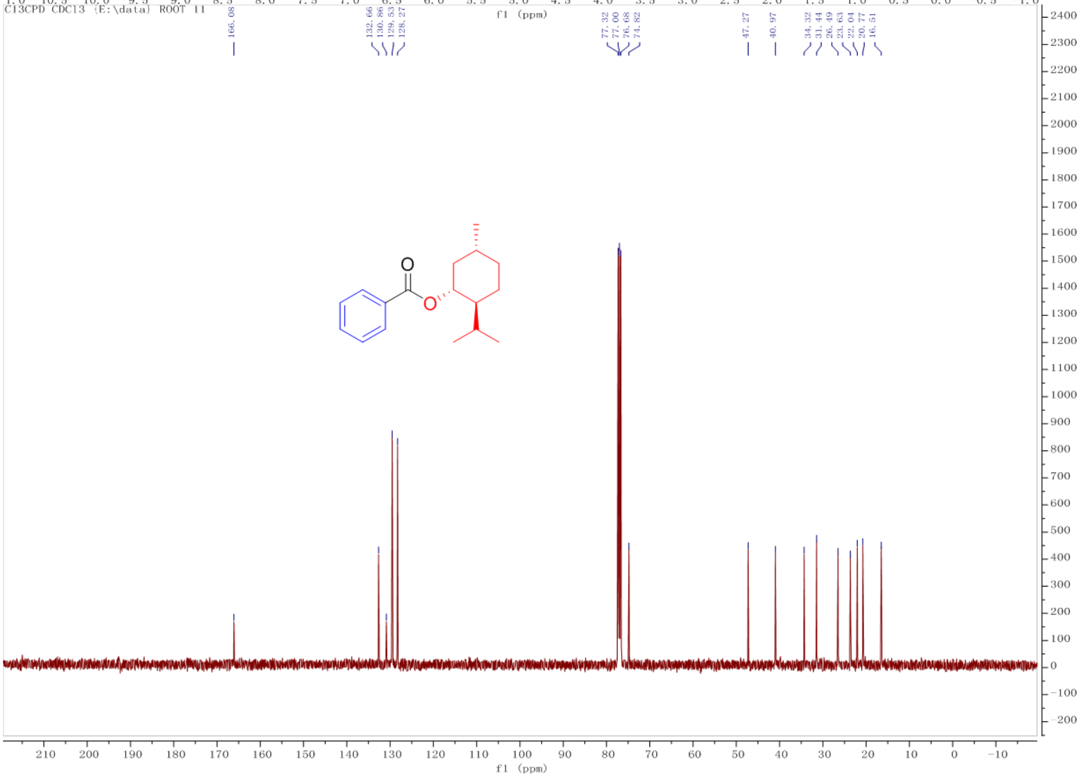
C13CPD CDCl3 {E:\data} ROOT 11
166.08
132.66
130.88
129.53
128.27
77.32
77.00
76.68
74.82
47.27
40.97
34.32
31.44
26.49
23.63
22.04
20.77
16.51
f1 (ppm)

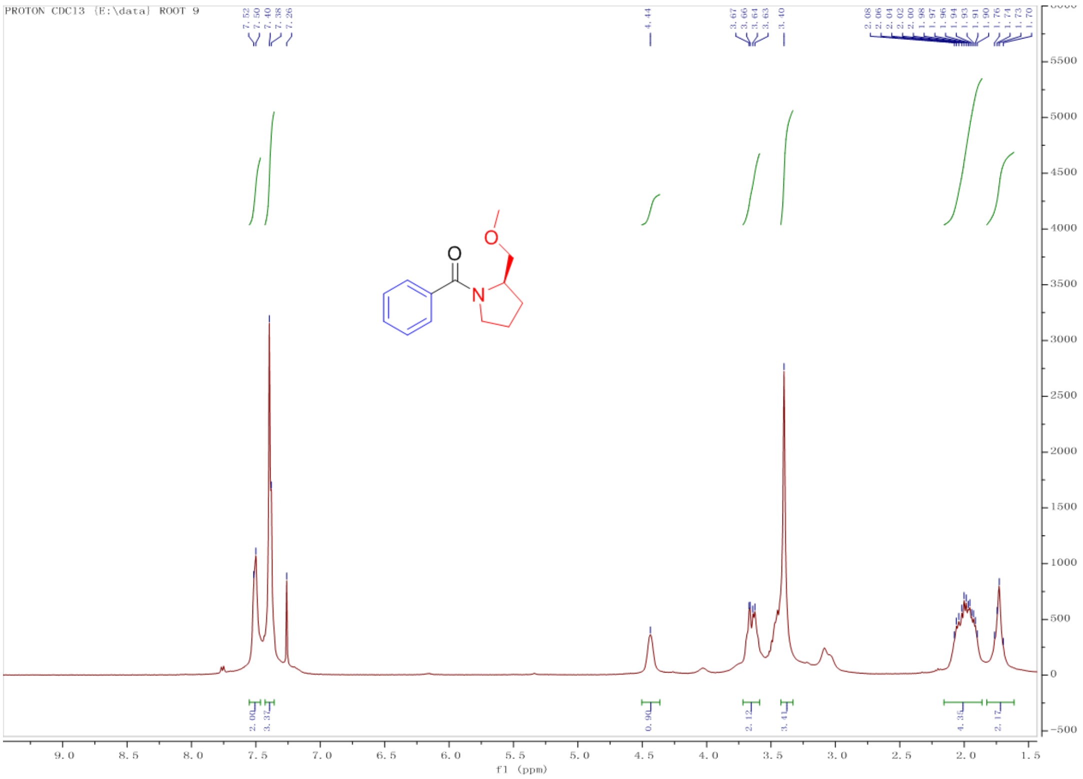
PROTON CDCl3 {E:\data} ROOT 9
7.52
7.50
7.40
7.38
7.26
4.44
3.67
3.66
3.64
3.63
3.40
2.00
3.37
0.90
2.12
3.41
4.35
2.17
f1 (ppm)

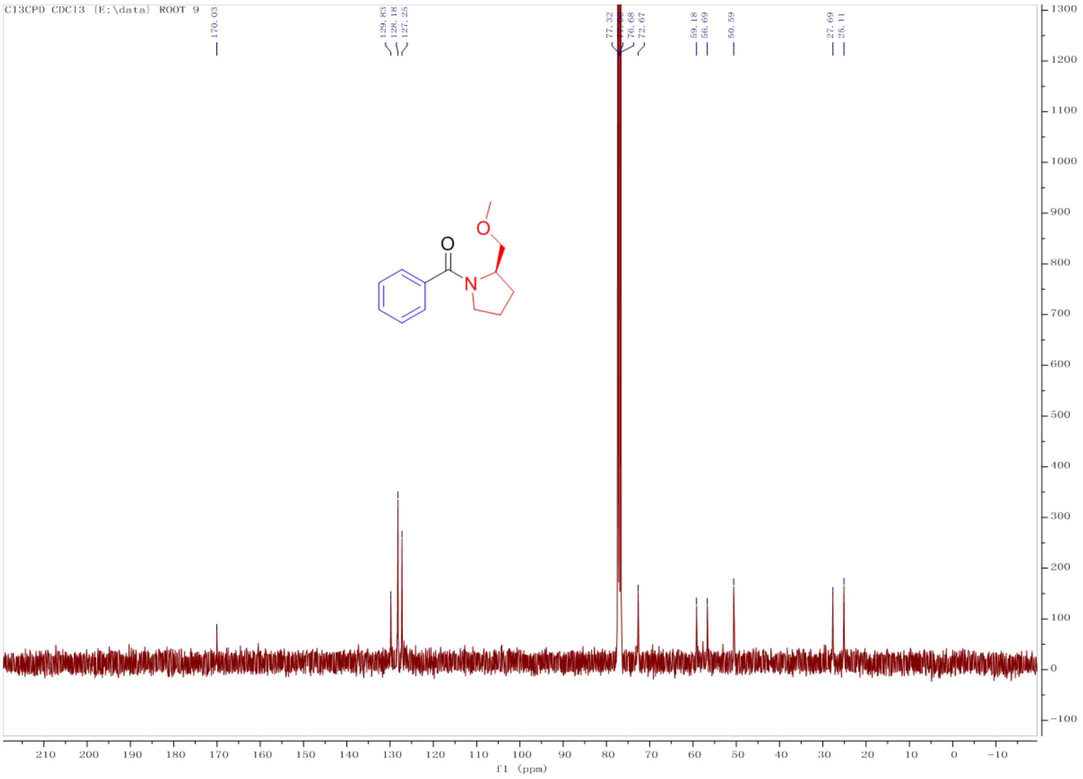
C13CPD CDCl3 {E:\data} ROOT 9
170.03
129.83
128.18
127.25
77.32
76.68
72.67
59.18
56.69
50.59
27.69
25.11
f1 (ppm)

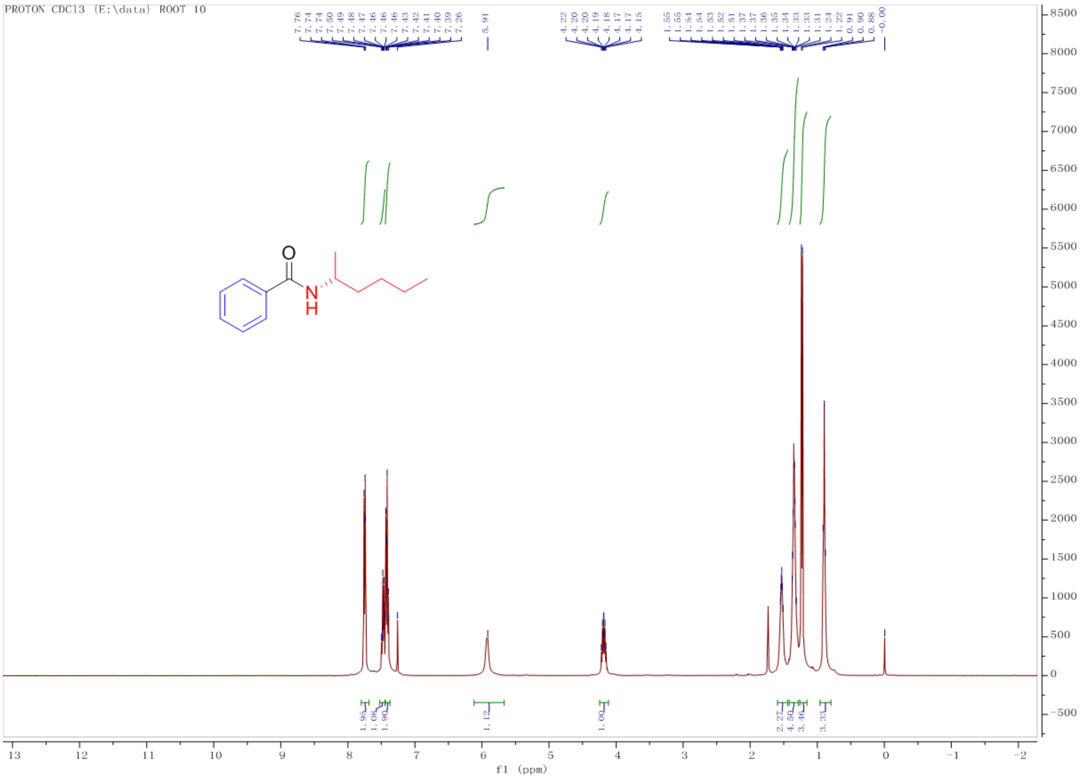
PROTON CDCl3 {E:\data} ROOT 10
f1 (ppm)

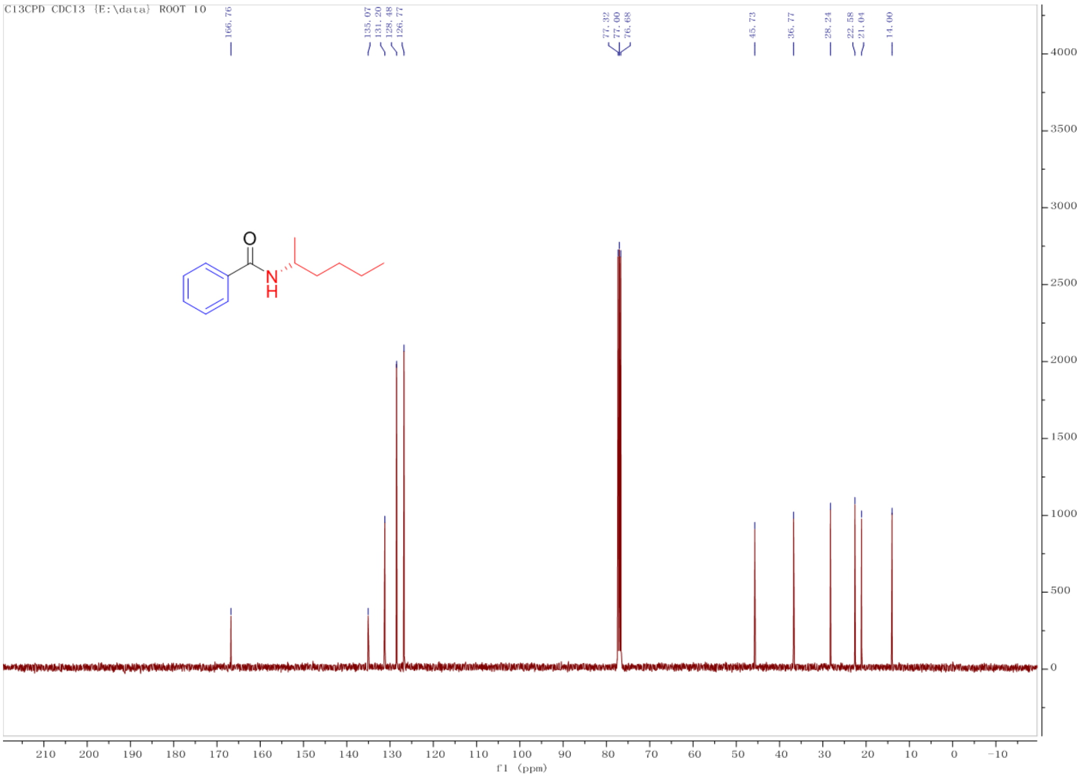
C13CPD CDCl3 {E:\data} ROOT 10
166.76
135.07
131.20
128.48
126.77
77.32
77.00
76.68
45.73
36.77
28.24
22.58
21.04
14.00
f1 (ppm)

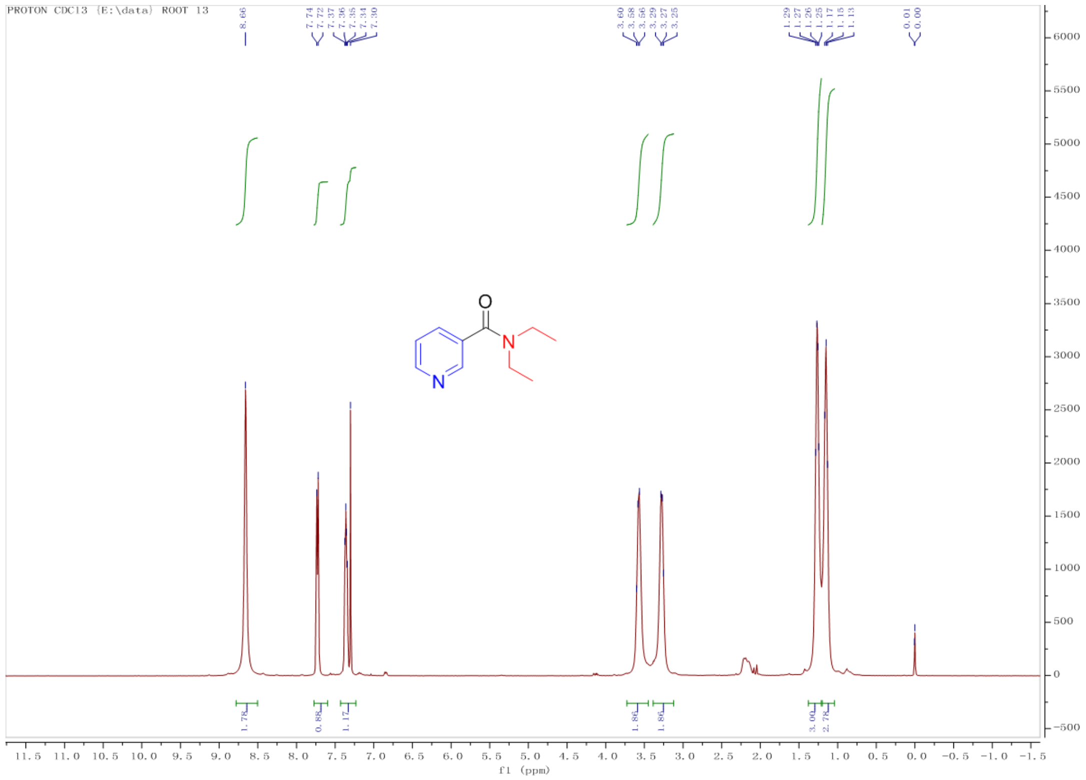
PROTON CDCl3 {E:\data} ROOT 13
8.66
7.74
7.72
7.37
7.36
7.35
7.34
7.30
3.60
3.58
3.56
3.29
3.27
3.25
1.29
1.27
1.26
1.25
1.17
1.15
1.13
0.01
0.00
1.78
0.88
1.17
1.86
1.86
3.00
2.78
f1 (ppm)

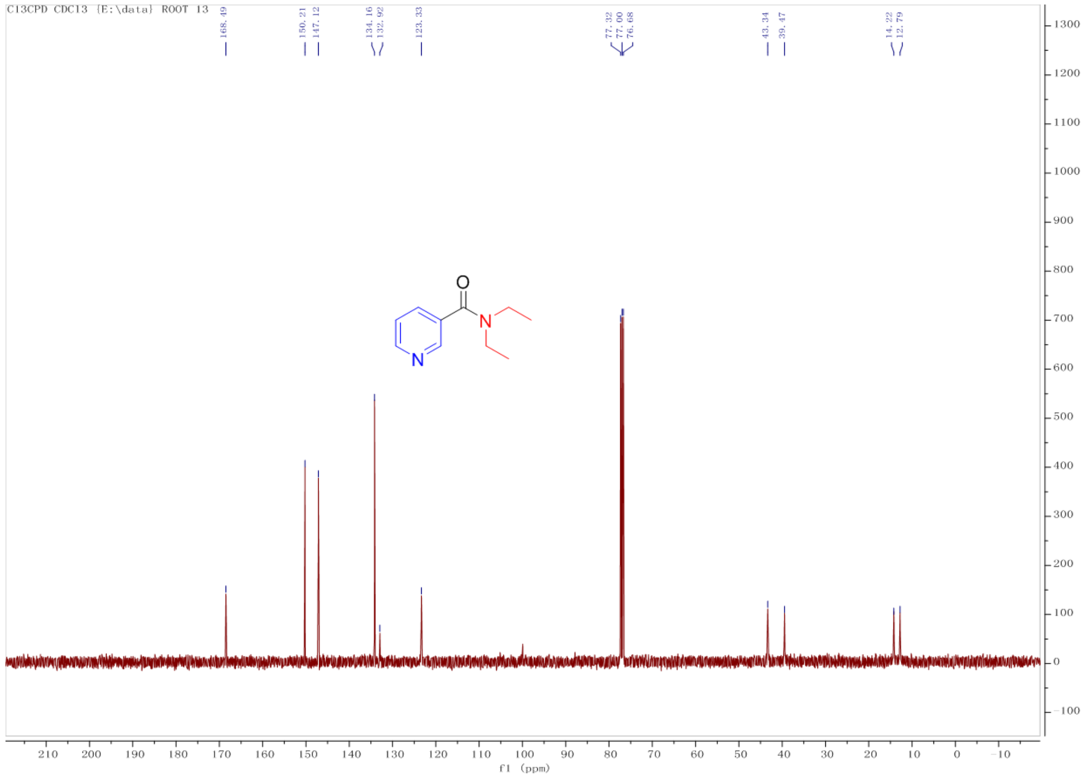
C13CPD CDCl3 {E:\data} ROOT 13
168.49
150.21
147.12
134.16
132.92
123.33
77.32
77.00
76.68
43.34
39.47
14.22
12.79
f1 (ppm)

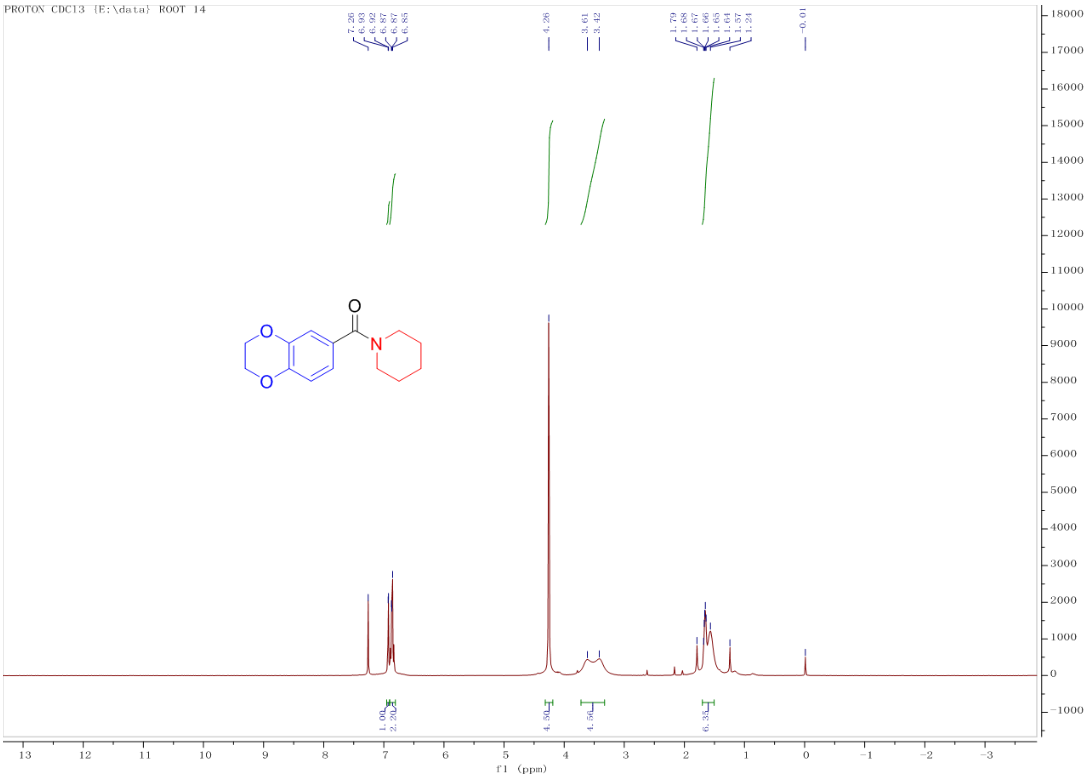
PROTON CDCl3 {E:\data} ROOT 14
7.26
6.93
6.92
6.87
6.87
6.85
4.26
3.61
3.42
1.79
1.68
1.67
1.66
1.65
1.64
1.57
1.24
-0.01
1.00
2.20
4.50
4.56
6.35
f1 (ppm)

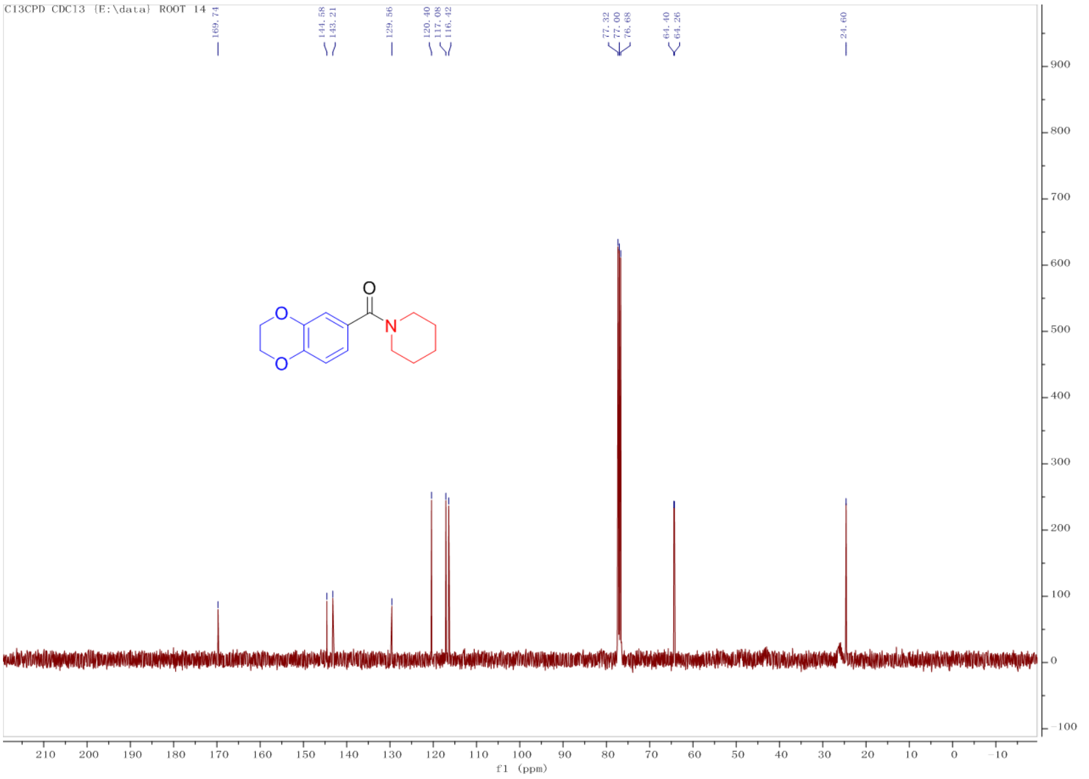
C13CPD CDCl3 {E:\data} ROOT 14
169.74
144.58
143.21
129.56
120.40
117.08
116.42
77.32
77.00
76.68
64.40
64.26
24.60
f1 (ppm)

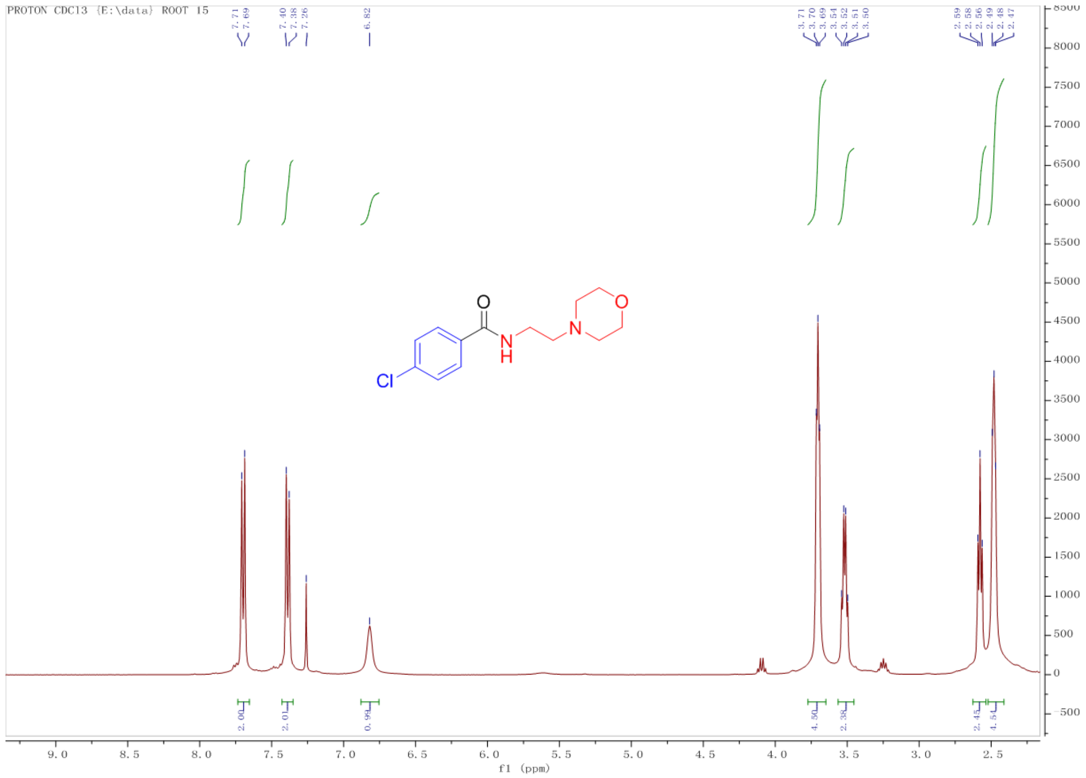
PROTON CDCl3 {E:\data} ROOT 15
7.71
7.69
7.40
7.38
7.26
6.82
3.71
3.70
3.69
3.54
3.52
3.51
3.50
2.59
2.58
2.56
2.49
2.48
2.47
2.00
2.01
0.96
4.50
2.38
2.45
4.54
O
N
H
N
O
Cl
f1 (ppm)

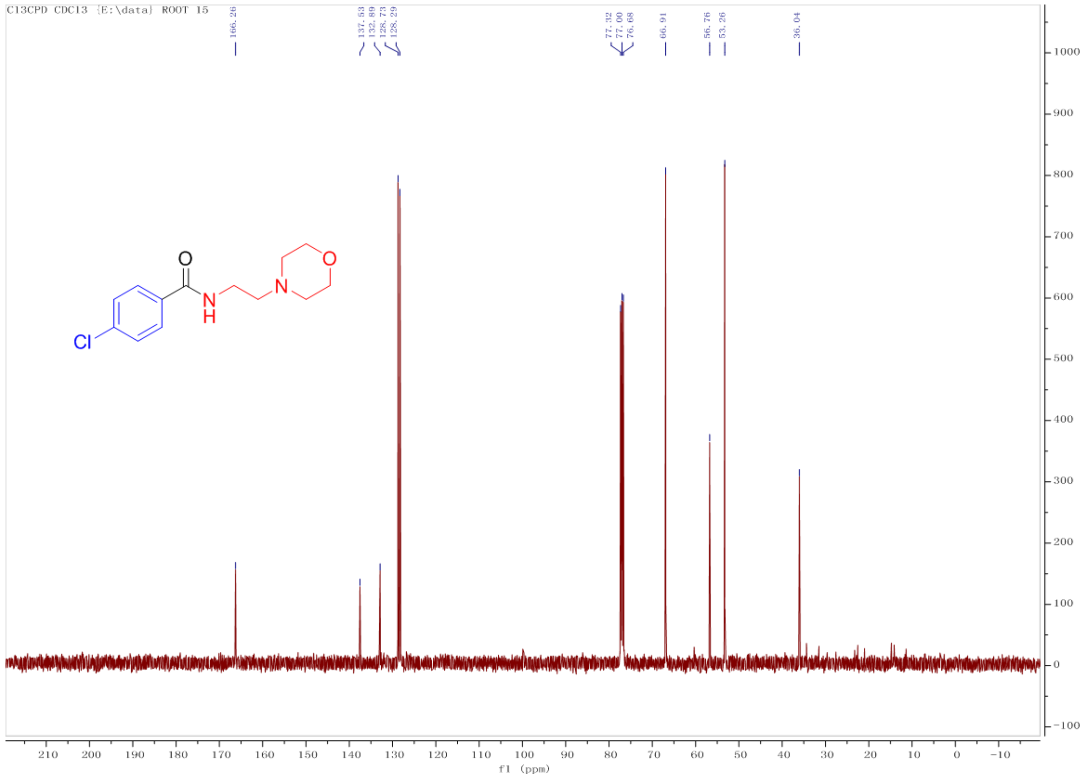
C13CPD CDCl3 {E:\data} ROOT 15
166.26
137.53
132.89
128.73
128.29
77.32
77.00
76.68
66.91
56.76
53.26
36.04
O
N
H
N
O
Cl
f1 (ppm)

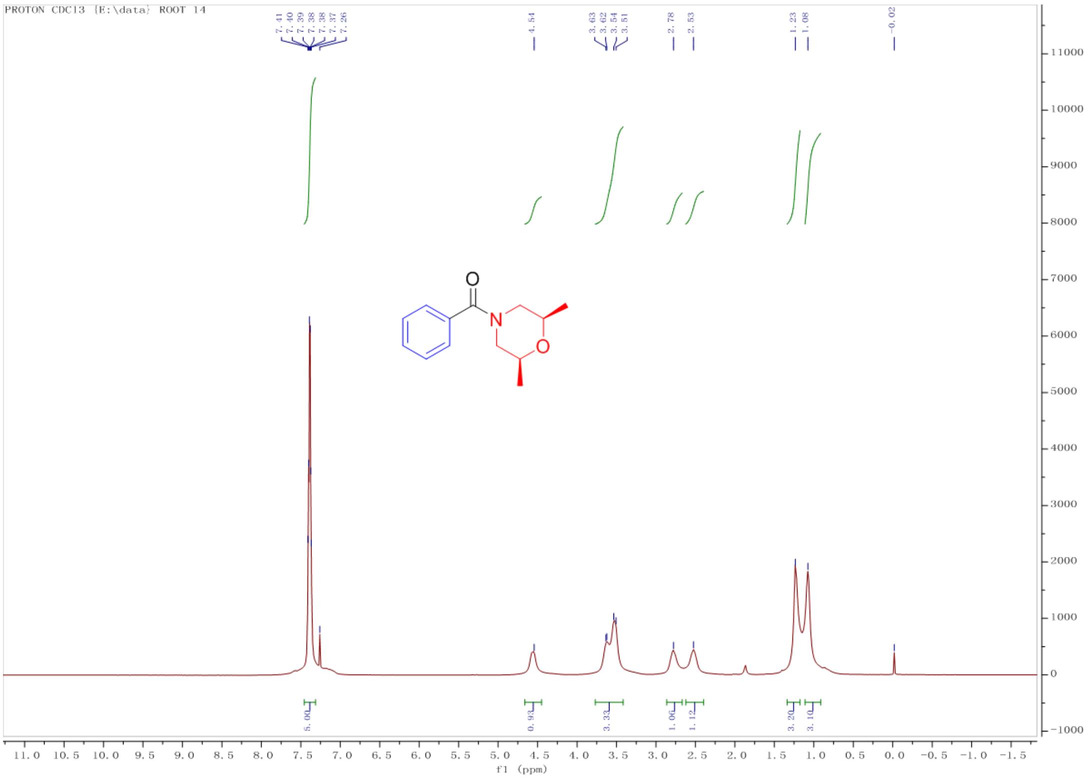
PROTON CDCl3 {E:\data} ROOT 14
7.41
7.40
7.39
7.38
7.38
7.37
7.26
4.54
3.63
3.62
3.54
3.51
2.78
2.53
1.23
1.08
-0.02
5.00
0.93
3.33
1.06
1.12
3.20
3.10
f1 (ppm)

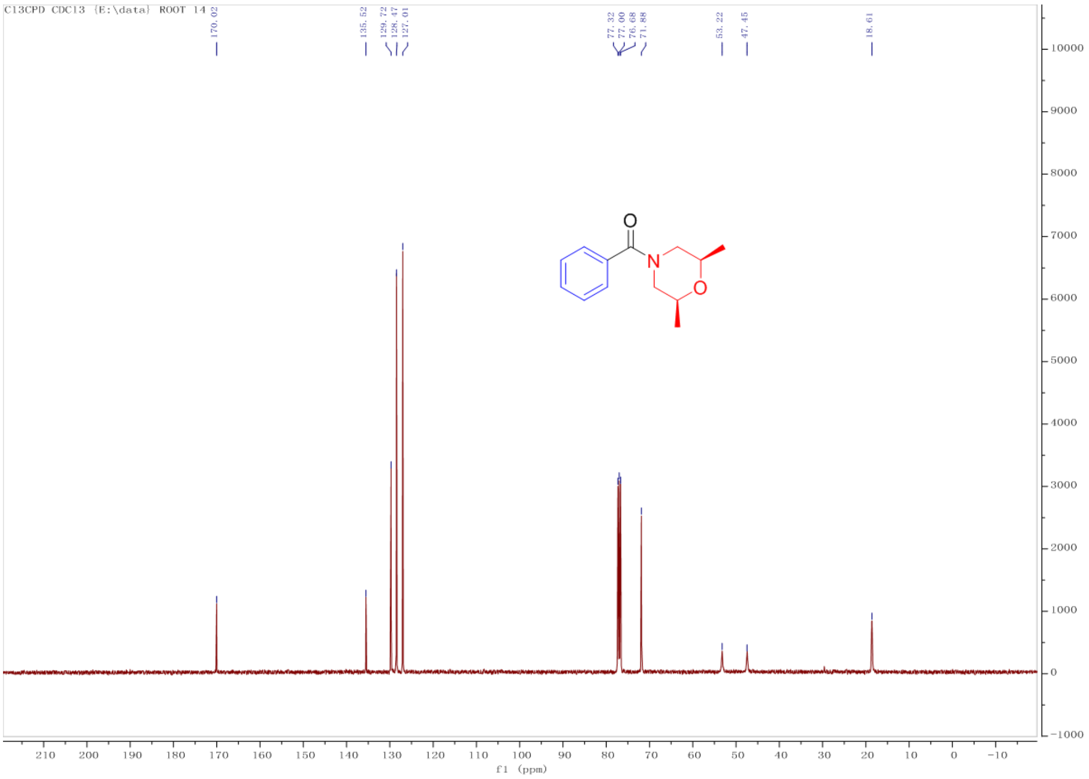
C13CPD CDCl3 {E:\data} ROOT 14
170.02
135.52
129.72
128.47
127.01
77.32
77.00
76.68
71.88
53.22
47.45
18.61
f1 (ppm)

5. Supplementary References

1. Qureshi, Z. S., Revankar, S. A., Khedkar, M. V. & Bhanage, B. M. Aminocarbonylation of aryl iodides with primary and secondary amines in aqueous medium using polymer supported palladium-N-heterocyclic carbene complex as an efficient and heterogeneous recyclable catalyst. *Catal. Today* **198**, 148-153 (2012).
2. Mane, R. S. & Bhanage, B. M. Carbonylative tertiary amide synthesis from aryl iodides and tertiary amines via oxidant-free c-n bond cleavage catalyzed by palladium (II) chloride in polyethylene glycol/water. *Adv. Synth. Catal.* **359**, 2621-2629 (2017).
3. Tambade, P. J., Patil, Y. P. & Bhanage, B. M. Palladium bis(2,2,6,6-tetramethyl-3,5-heptanedionate) catalyzed alkoxycarbonylation and aminocarbonylation reactions. *Appl. Organomet. Chem.* **23**, 235-240 (2009).
4. Bhanage, B., Tambade, P., Patil, Y. & Bhanushali, M. $Pd(OAc)_2$-catalyzed aminocarbonylation of aryl iodides with aromatic or aliphatic amines in water. *Synthesis* **2008**, 2347-2352 (2008).
5. Adamcsik, B. *et al.* Palladium nanoparticles on a pyridinium supported ionic liquid phase: a recyclable and low-leaching palladium catalyst for aminocarbonylation reactions. *RSC Adv.* **10**, 23988-23998 (2020).
6. Prasad, A. V., Biying, A. O., Ling, W. Y., Stubbs, L. P. & Zhu, Y. Synthesis and new application of green and recyclable cyclic poly (l-lactide)-clay hybrid. *J. Polym. Sci. A Polym. Chem.* **51**, 4167-4174 (2013).
7. Chen, B., Li, F., Huang, Z., Lu, T. & Yuan, G. Stability or flexibility: Metal nanoparticles supported over cross-linked functional polymers as catalytic active sites for hydrogenation and carbonylation. *Appl. Catal. A: Gen.* **481**, 54-63 (2014).
8. Zhu, Y. *et al.* Stabilized well-dispersed Pd(0) nanoparticles for aminocarbonylation of aryl halides. *Dalton Trans.* **40**, 9320-9325 (2011).
9. Ai, H. J. *et al.* Iron-Catalyzed Alkoxycarbonylation of Alkyl Bromides via a Two-Electron Transfer Process. *Angew. Chem. Int. Ed.* **61**, e202211939 (2022).
10. Li, Y. & Wu, X.-F. Copper/iron co-catalyzed alkoxycarbonylation of unactivated alkyl bromides. *Commun. Chem.* **1**, 1-8 (2018).
11. Dang, T. T. *et al.* Palladium Nanoparticles Supported on ZIF-8 As an Efficient Heterogeneous Catalyst for Aminocarbonylation. *ACS Catal.* **3**, 1406-1410 (2013).
12. Forni, J. A., Micic, N., Connell, T. U., Weragoda, G. & Polyzos, A. Tandem photoredox catalysis: Enabling carbonylative amidation of aryl and alkylhalides. *Angew. Chem. Int. Ed.* **59**, 18646-18654 (2020).
13. Roice, M., Christensen, S. F. & Meldal, M. Ultramine: A high-capacity polyethylene-imine-based polymer and its application as a scavenger resin. *Chem. Eur. J.* **10**, 4407-4415 (2004).
14. Bantreil, X., Fleith, C., Martinez, J. & Lamaty, F. Copper-catalyzed direct synthesis of benzamides from alcohols and amines. *ChemCatChem* **4**, 1922-1925 (2012).
15. Li, Y., Wang, Y. & Wang, J. Microwave-assisted synthesis of amides from various amines and benzoyl chloride under solvent-free conditions: A rapid and efficient method for selective protection of diverse amines. *Russ. J. Org. Chem* **44**, 358-361 (2008).
16. Durgaiah, C. *et al.* Transamidation of carboxamides with amines over nanosized zeolite beta under solvent-free conditions. *Catal. Commun.* **81**, 29-32 (2016).
17. Wu, J. W., Wu, Y. D., Dai, J. J. & Xu, H. J. Benzoic acid-catalyzed transamidation reactions of carboxamides, phthalimide, ureas and thioamide with amines. *Adv. Synth. Catal.* **356**, 2429-

2436 (2014).

18. Stopka, T. *et al.* Electrophilic activation of amides for the preparation of polysubstituted pyrimidines. *Synthesis* **51**, 194-202 (2019).

19. Khedkar, M. V., Sasaki, T. & Bhanage, B. M. Immobilized palladium metal-containing ionic liquid-catalyzed alkoxycarbonylation, phenoxycarbonylation, and aminocarbonylation reactions. *ACS Catal.* **3**, 287-293 (2013).

20. Liu, S., Wang, H., Dai, X. & Shi, F. Organic ligand-free carbonylation reactions with unsupported bulk Pd as catalyst. *Green Chem.* **20**, 3457-3462 (2018).

21. Lei, Y. *et al.* Palladium supported on triphenylphosphine functionalized porous organic polymer: A highly active and recyclable catalyst for alkoxycarbonylation of aryl iodides. *J. Mol. Catal. A: Chem.* **398**, 164-169 (2015).

22. Wang, Y., Zhi, H. & Luo, J. A facile and efficient protocol for esterification and acetalization in a PEG1000-D (A) IL/toluene thermoregulated catalyst–media combined systems. *J. Mol. Catal. A: Chem.* **379**, 46-52 (2013).

23. Zhao, L. *et al.* Acceptorless dehydrogenative cross-coupling of primary alcohols catalyzed by an N-heterocyclic carbene–nitrogen–phosphine chelated ruthenium (II) complex. *J. Org. Chem.* **87**, 4550-4559 (2022).

24. El Ali, B., El-Ghanam, A. M. & Fettouhi, M. $H_{3+n}PMo_{12-n}V_nO_{40}$-catalyzed selective oxidation of benzoins to benzils or aldehydes and esters by dioxygen. *J. Mol. Catal. A: Chem.* **165**, 283-290 (2001).

25. Hatano, M. *et al.* Metal-free transesterification catalyzed by tetramethylammonium methyl carbonate. *Green Chem.* **20**, 1193-1198 (2018).

26. Zhang, Z. *et al.* Hierarchical Zn/Ni-MOF-2 nanosheet-assembled hollow nanocubes for multicomponent catalytic reactions. *Angew. Chem. Int. Ed.* **53**, 12517-12521 (2014).

27. Shang, R. *et al.* Synthesis of aromatic esters via Pd-catalyzed decarboxylative coupling of potassium oxalate monoesters with aryl bromides and chlorides. *J. Am. Chem. Soc.* **131**, 5738-5739 (2009).

28. Wang, S. *et al.* Enantioselective access to chiral aliphatic amines and alcohols via Ni-catalyzed hydroalkylations. *Nat. Commun.* **12**, 1-9 (2021).

29. Tinnis, F., Volkov, A., Slagbrand, T. & Adolfsson, H. Chemoselective reduction of tertiary amides under thermal control: formation of either aldehydes or amines. *Angew. Chem. Int. Ed.* **55**, 4562-4566 (2016).

30. Chen, W., Li, J., Xie, H. & Wang, J. Rhodium (III)-catalyzed asymmetric addition of inert arene C–H bond to aldehydes to afford enantioenriched phthalides. *Org. Lett.* **22**, 3586-3590 (2020).

31. Weires, N. A., Caspi, D. D. & Garg, N. K. Kinetic modeling of the nickel-catalyzed esterification of amides. *ACS Catal.* **7**, 4381-4385 (2017).
