# Supplementary figures and images for: Constructing multiple active sites in iron oxide catalysts for improving carbonylation reactions
Source: Nat Commun. 2023 Aug 17;14:4973. doi: 10.1038/s41467-023-40640-z (PMC10435489; doi:10.1038/s41467-023-40640-z)

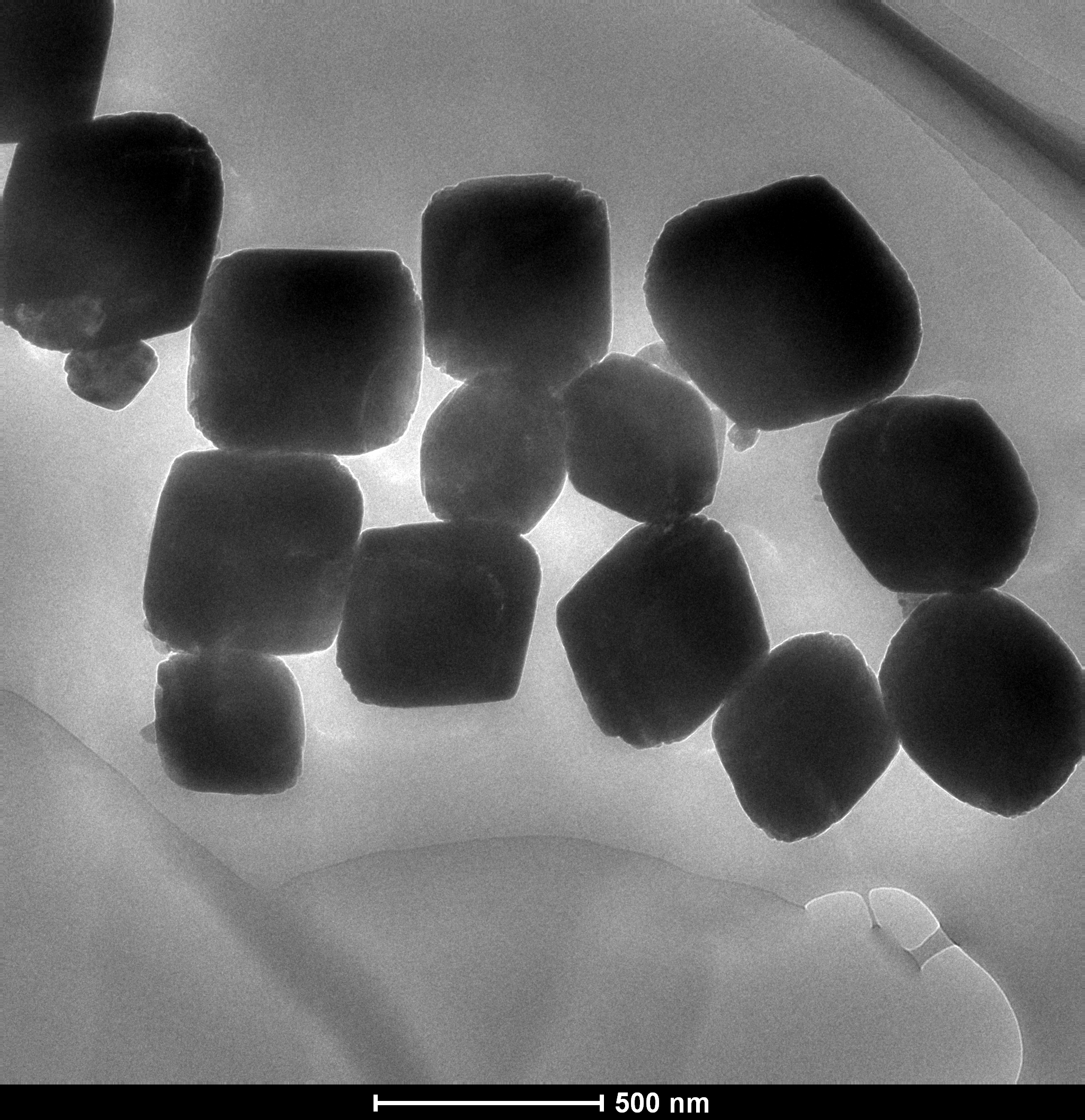

Supplement: Supplementary file 3 — Source Data [file 41467_2023_40640_MOESM3_ESM.zip › TEM/0.5 Fe2O3-Ovac#_0000.tif]

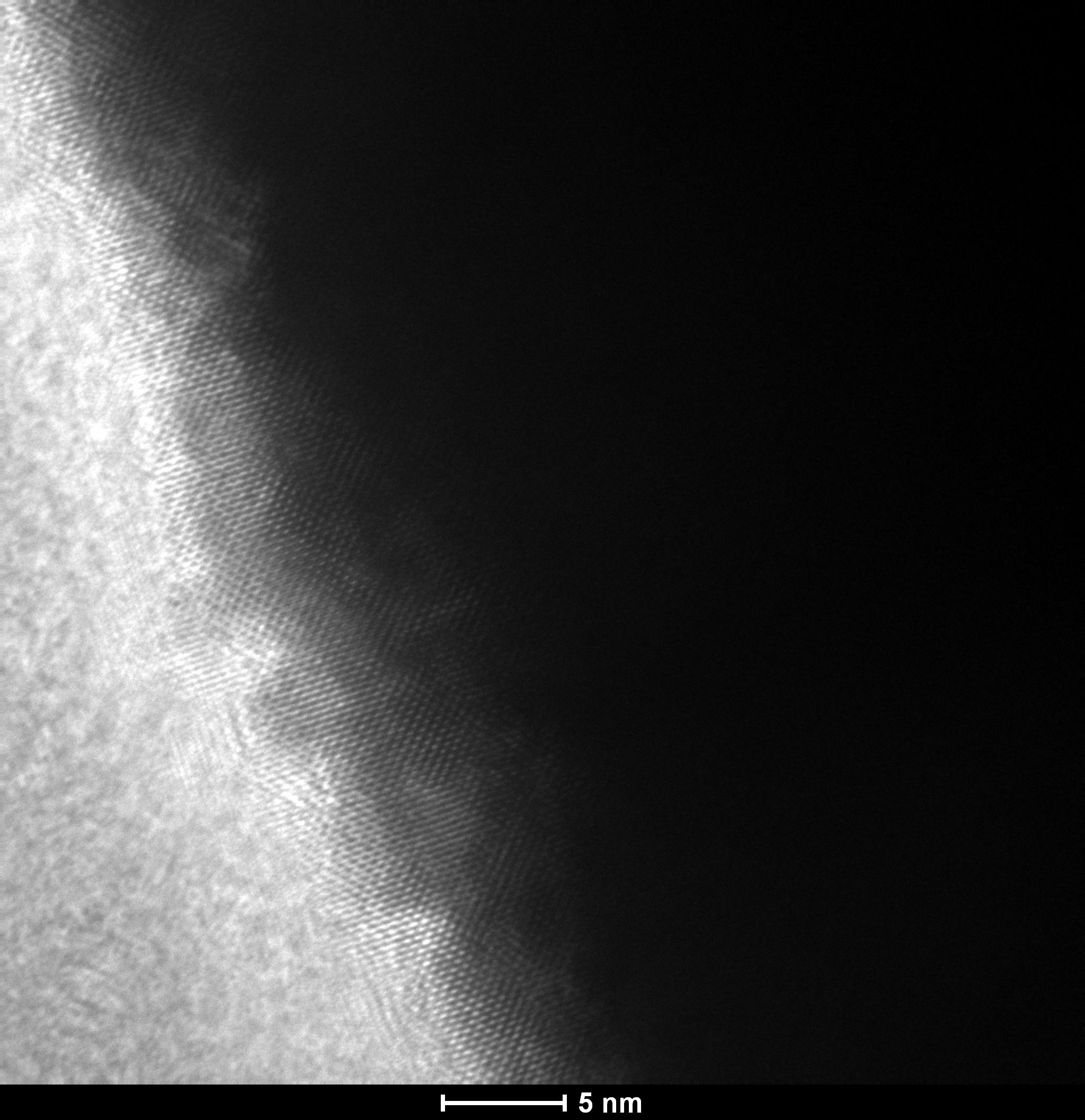

Supplement: Supplementary file 3 — Source Data [file 41467_2023_40640_MOESM3_ESM.zip › TEM/0.5 Fe2O3-Ovac#_0028.tif]

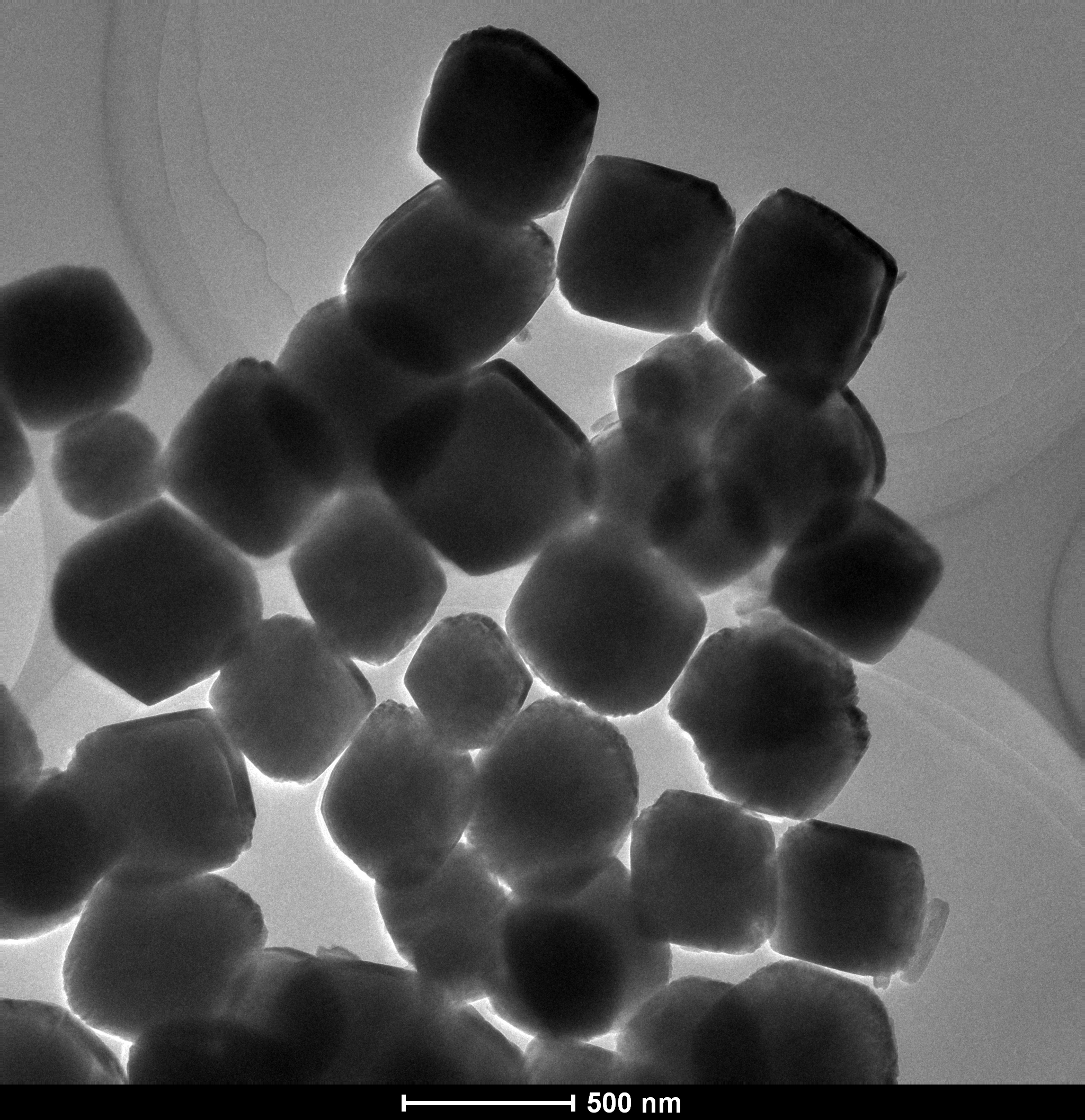

Supplement: Supplementary file 3 — Source Data [file 41467_2023_40640_MOESM3_ESM.zip › TEM/1.0 Fe2O3-Ovac#_0002.tif]

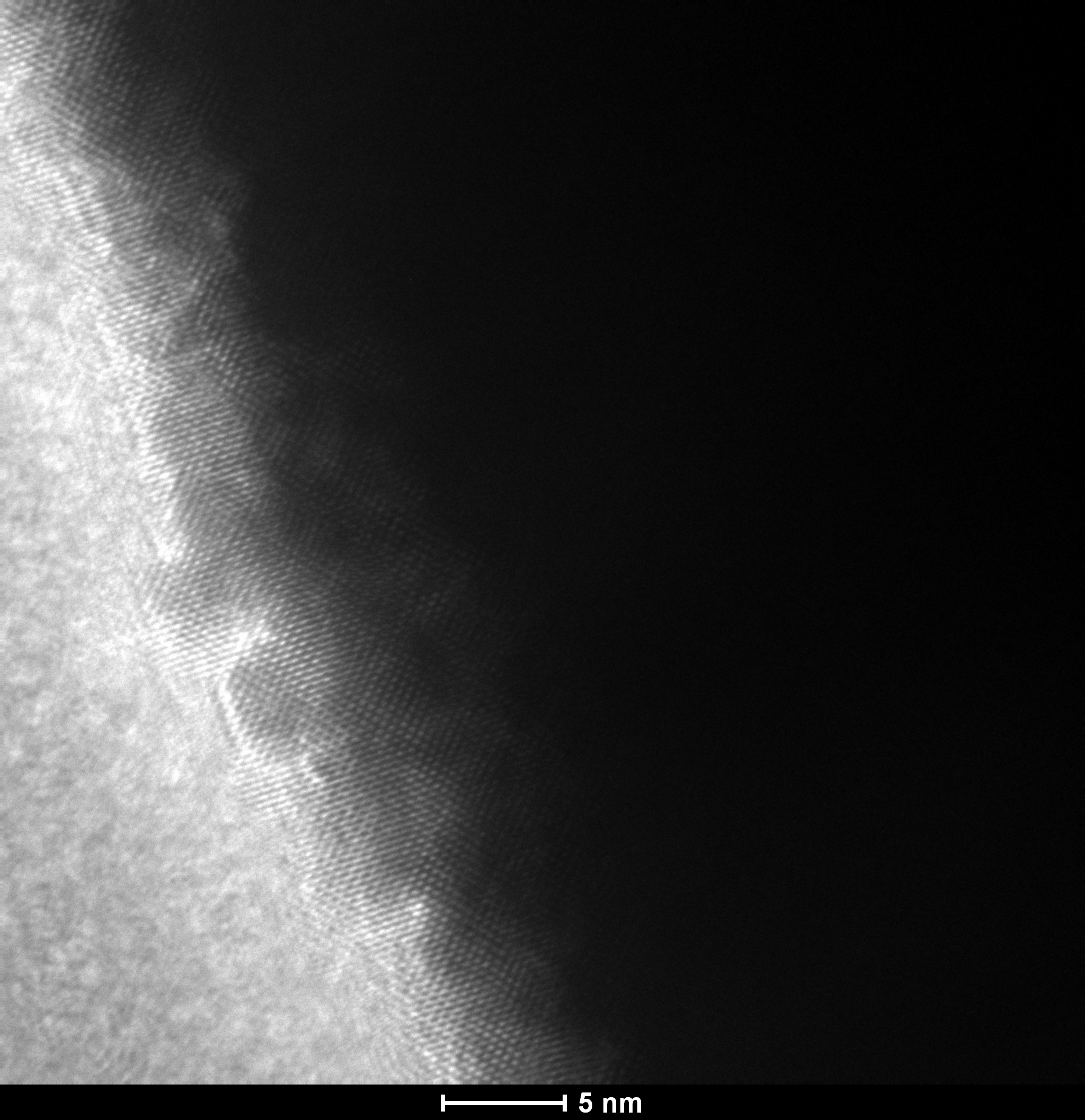

Supplement: Supplementary file 3 — Source Data [file 41467_2023_40640_MOESM3_ESM.zip › TEM/1.0 Fe2O3-Ovac#_0018.tif]

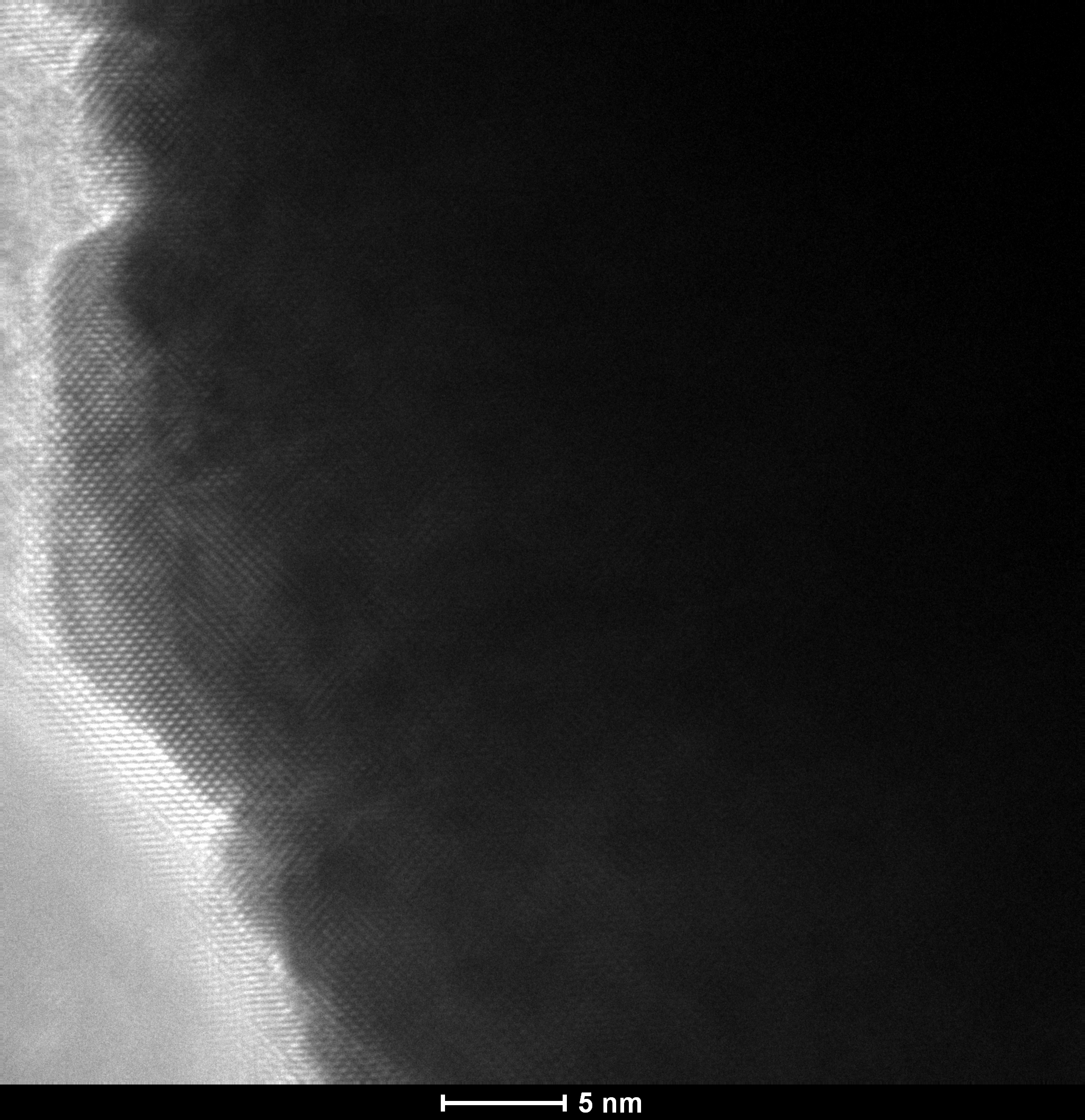

Supplement: Supplementary file 3 — Source Data [file 41467_2023_40640_MOESM3_ESM.zip › TEM/Fe2O3#_0014.tif]

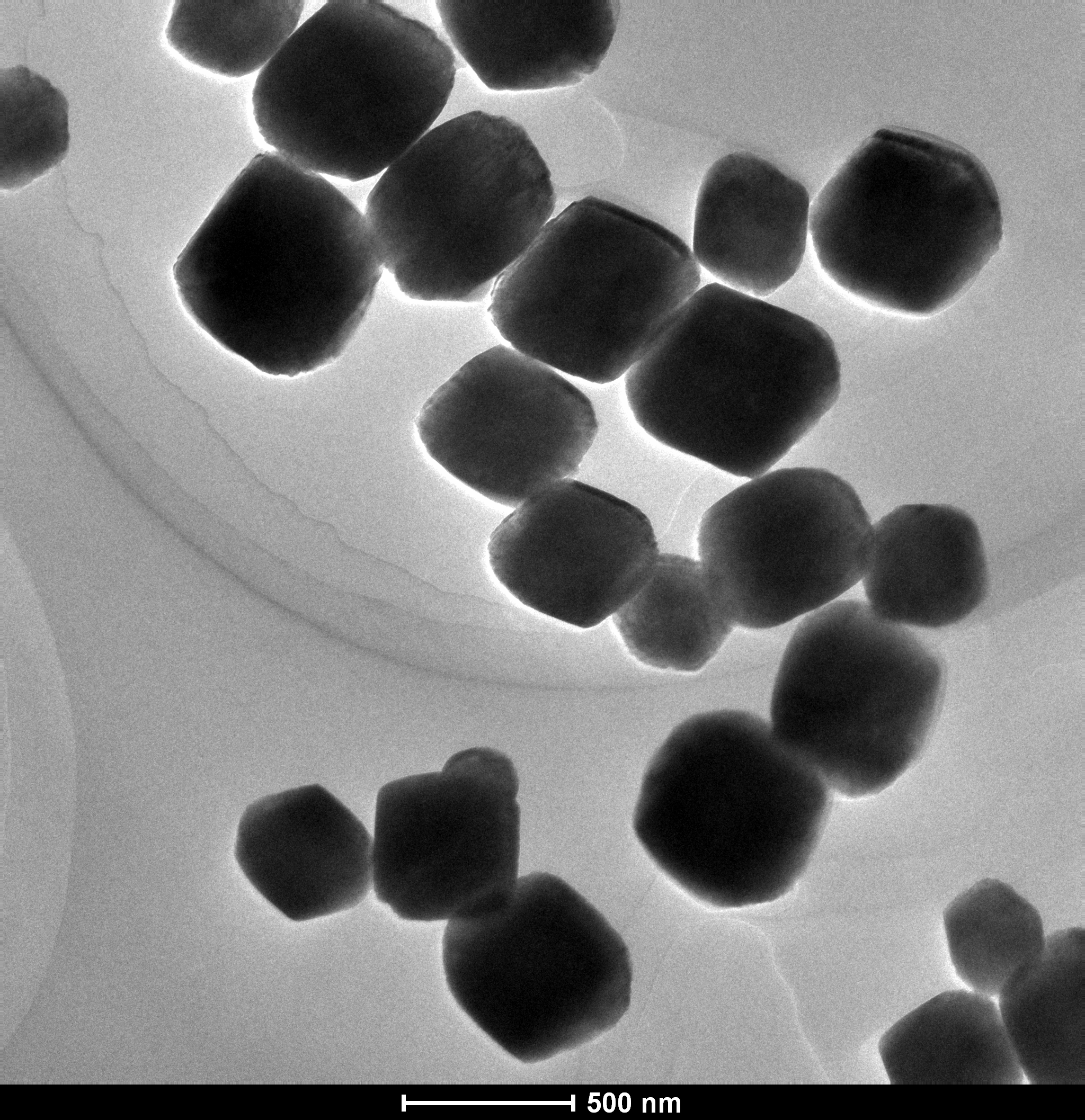

Supplement: Supplementary file 3 — Source Data [file 41467_2023_40640_MOESM3_ESM.zip › TEM/Fe2O3#_0015.tif]

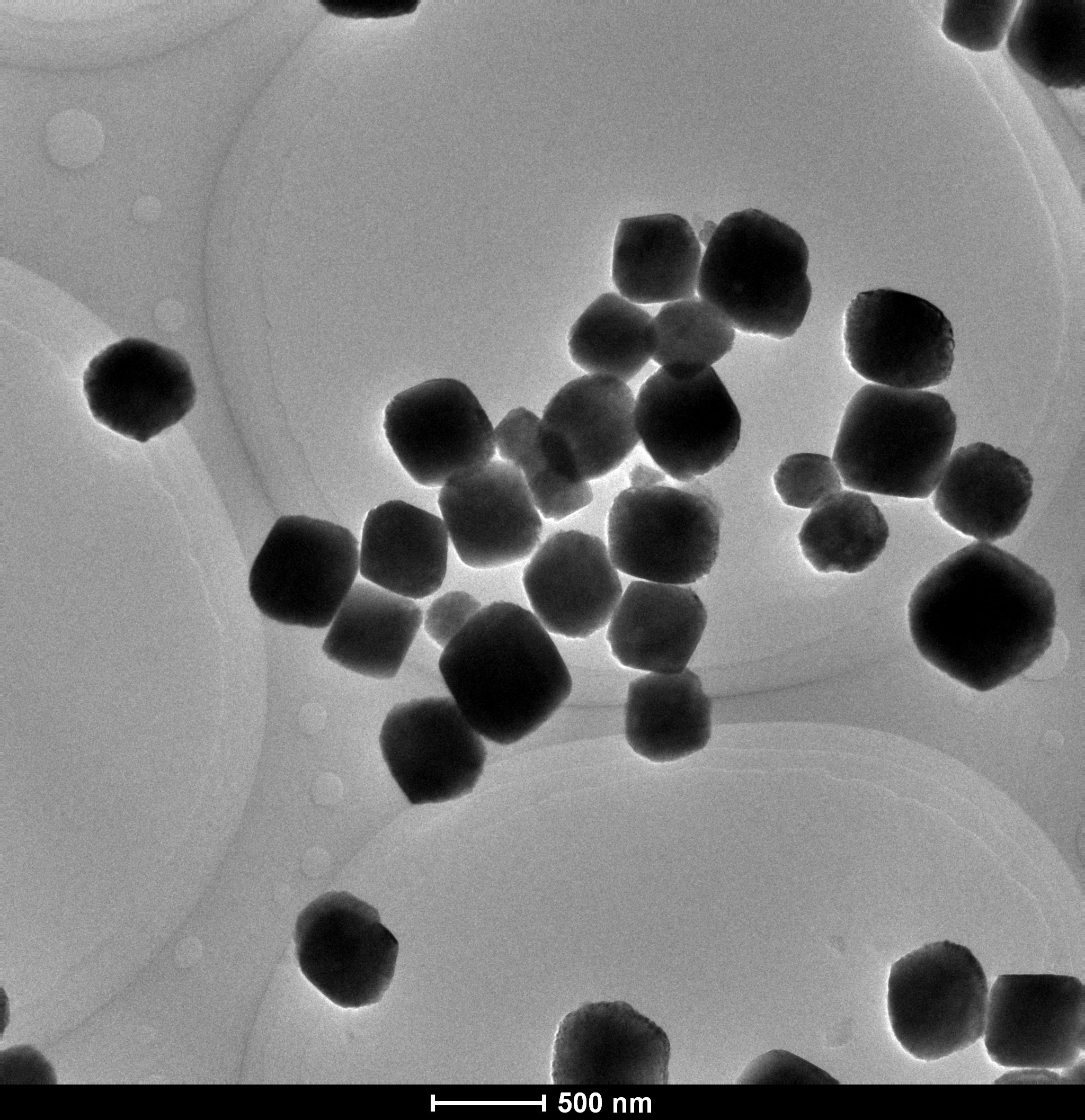

Supplement: Supplementary file 3 — Source Data [file 41467_2023_40640_MOESM3_ESM.zip › TEM/Used_0000.tif]

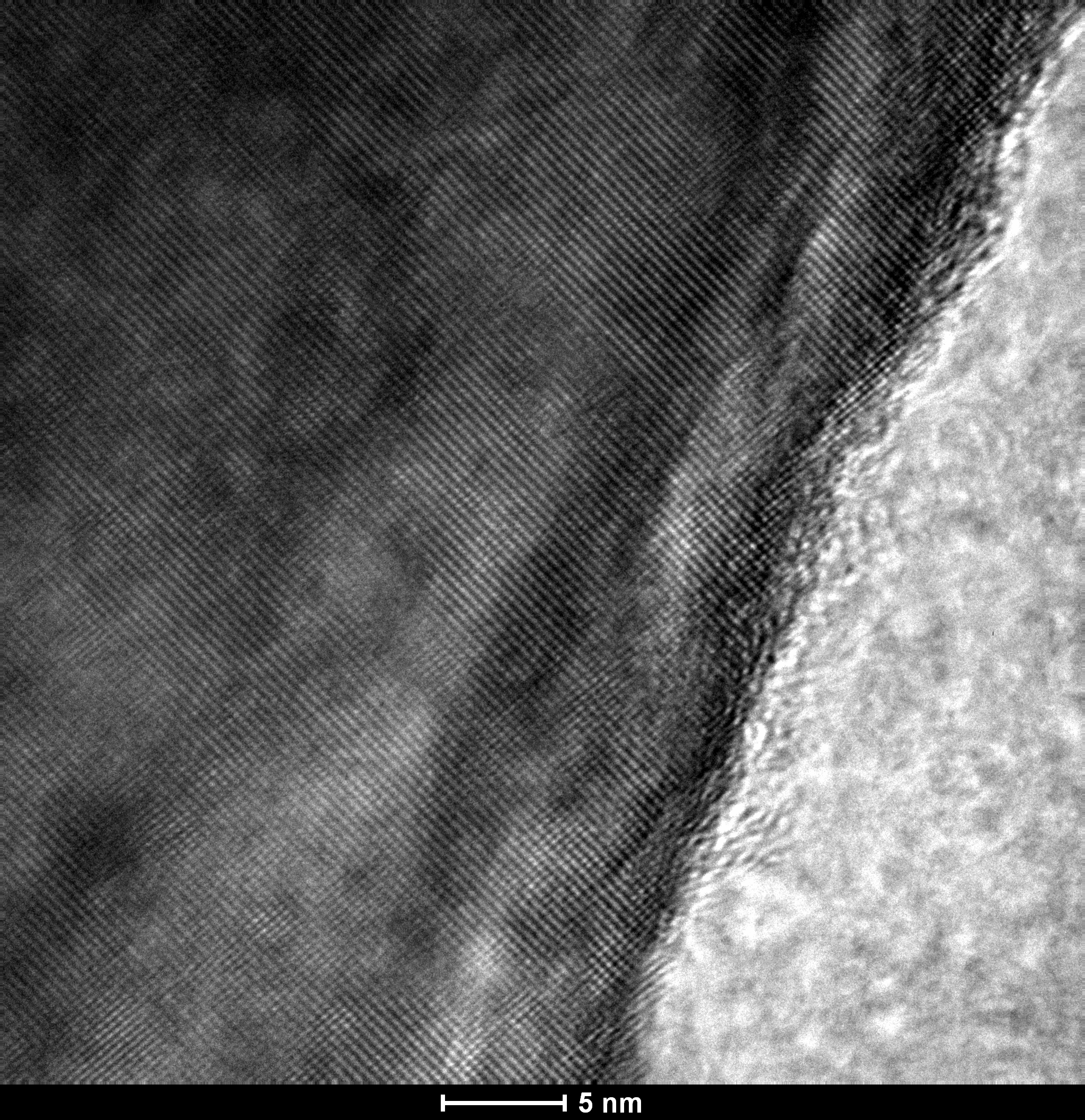

Supplement: Supplementary file 3 — Source Data [file 41467_2023_40640_MOESM3_ESM.zip › TEM/Used_0007.tif]
